# The Smelling Principle of Vetiver Oil, Unveiled by Chemical Synthesis

**DOI:** 10.1002/anie.202014609

**Published:** 2021-02-09

**Authors:** Jie Ouyang, Hanyong Bae, Samuel Jordi, Quang Minh Dao, Sandro Dossenbach, Stefanie Dehn, Julia B. Lingnau, Chandra Kanta De, Philip Kraft, Benjamin List

**Affiliations:** ^1^ Max-Planck-Institut für Kohlenforschung Kaiser-Wilhelm-Platz 1 45470 Mülheim an der Ruhr Germany; ^2^ Givaudan Schweiz AG Fragrances S&T, Ingredients Research Kemptpark 50 8310 Kemptthal Switzerland; ^3^ Department of Chemistry Sungkyunkwan University 2066, Seobu-ro Jangan-gu Suwon 16419 Republic of Korea

**Keywords:** 2-*epi*-ziza-6(13)en-3-one, asymmetric Mukaiyama–Michael addition, enantioselective synthesis, smelling principle, vetiver oil

## Abstract

Vetiver oil, produced on a multiton‐scale from the roots of vetiver grass, is one of the finest and most popular perfumery materials, appearing in over a third of all fragrances. It is a complex mixture of hundreds of molecules and the specific odorant, responsible for its characteristic suave and sweet transparent, woody‐ambery smell, has remained a mystery until today. Herein, we prove by an eleven‐step chemical synthesis, employing a novel asymmetric organocatalytic Mukaiyama–Michael addition, that (+)‐2‐epi‐ziza‐6(13)en‐3‐one is the active smelling principle of vetiver oil. Its olfactory evaluation reveals a remarkable odor threshold of 29 picograms per liter air, responsible for the special sensuous aura it lends to perfumes and the quasi‐pheromone‐like effect it has on perfumers and consumers alike.

## Graphical abstract

Despite the advance of modern analytic techniques, the smelling principle of vetiver oil is still unknown owing to its extremely complex matrix of over 150 compounds. A concise stereoselective total synthesis proves (+)‐2‐*epi*‐ziza‐6(13)en‐3‐one to be the key to the quasi‐pheromone‐like aura of vetiver that magically resembles the effect of Iso E Super.

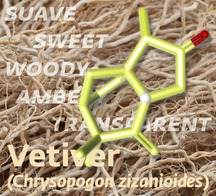

From “Chanel N°5” (1921) by Ernest Beaux and “Vetiver de Guerlain” (1959) by Jean‐Paul Guerlain to the extreme dose of 90 % in the rebellious “Turtle Vetiver” (LesNez, 2019) by Isabelle Doyen, vetiver oil is omnipresent in perfumery.[^1^, ^2^] No other fragrance material appears more often in the name of a perfume: fragrantica.com currently lists 2267 masculine, 1976 feminine, and 2302 unisex perfumes centered on this, in the truest sense, essential oil. It is a chameleon in applications and can, for instance, be used to make a classic cologne more masculine or to ground lush feminine florals with a touch of earthiness.^[3]^ This versatility is due to a characteristic suave and sweet woody–ambery transparency, an aura‐like effect as generally displayed by odorants of extremely low threshold, such as Paradisone [(+)‐(1*R*,2*S*,*Z*)‐methyl dihydrojasmonate]^[4]^ and rotundone.^[5]^ The essential oil of vetiver is obtained in 0.2–0.3 % yield by hydrodistillation of the dried roots of the tufted perennial grass *Chrysopogon zizanioides* (L.) Roberty. Despite a price of 350–500 $/kg, its worldwide consumption is estimated at 300–400 t/year, of which 60 % comes from the Les Cayes commune of Haiti.^[6]^ As one of the most complex essential oils, it contains several hundred sesquiterpene derivatives with a broad structural diversity, many of which are not yet identified. It is this complexity that has made the analysis of vetiver oil immensely challenging, despite the significant advances of modern analytical methods. No satisfactory reconstitution of this key perfumery material is available to the perfumer, and no single molecule conveys the typical vetiver character.^[5]^ In the odor profile of vetiver, a fresh hesperidic, citrusy grapefruit top connects with a dark and distinct suave and sweet transparent woody–ambery base to form a universal skeleton, a perfume within a perfume that can be interpreted in an almost infinite variety. While the citrusy grapefruit character is well understood and originates from α‐vetivone (**1**), β‐vetivone (**2**), and nootkatone (**3**), earthy aspects are due to (−)‐geosmine (**4**), with a little help by *nor*‐acorenone, and the creamy santal character mainly results from (*E*)‐isovalencenol (**5**, Figure 1). However, the typical transparent woody–ambery character of vetiver is still not understood, and its smelling principle remains enigmatic. Khusimone (**6**), present in about 2 % in the essential oil and first proposed as a smelling principle in 1980 by Maurer, is the only component the literature agrees upon to possess this highly desired vetiver character.^[7]^ An exhaustive analysis by Weyerstahl et al. in 2000 seemed to corroborate that finding, since all other vetiver odorants, such as eremophiladienal (**7**), 1,7‐cyclogerma‐1(10)‐4‐dienal (**8**), as well as ziza‐6(13)‐enone (**9**) and 2‐*epi*‐ziza‐6(13)‐enone (**10**), were described by them as khusimone‐like but weaker (Figure 1).^[8]^ However, with a relatively high threshold of 4.7 ng L^−1^ air, khusimone (**6**) cannot be the smelling principle of vetiver oil.

**Figure 1:**
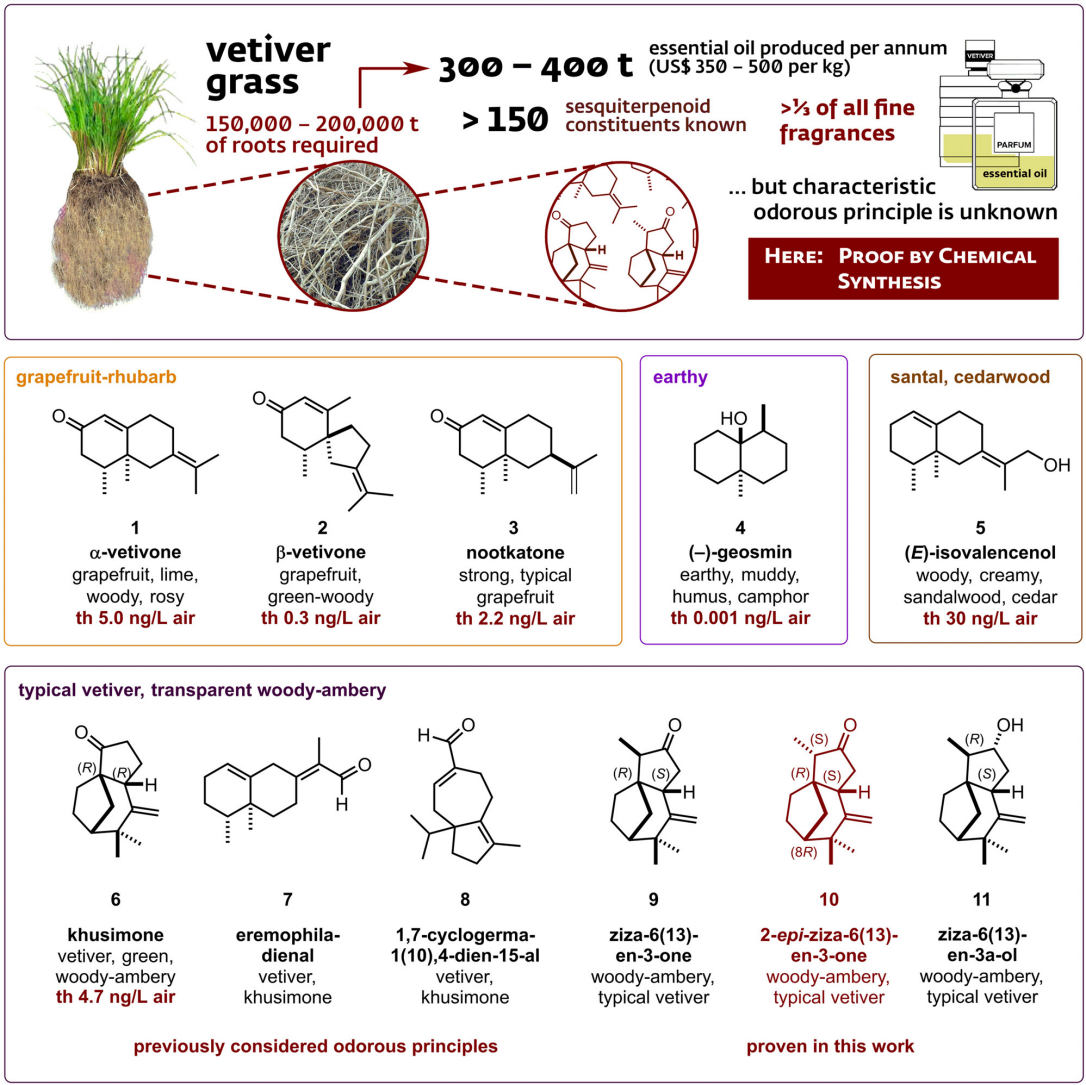
The important olfactory contributions to vetiver oil. α‐Vetivone (**1**), β‐vetivone (**2**), and nootkatone (**3**) are responsible for the grapefruit–rhubarb top note, while (−)‐geosmin (**4**) provides earthy aspects and (*E*)‐isovalencenol a creamy, cedar‐ and sandalwood‐type background. The highly desired transparent woody–amber note has remained a mystery. Previously suggested but not unambiguously confirmed substances include eremophiladienal (**7**) and 1,7‐cyclogerma‐1(10),4‐dien‐15‐al (**8**), khusimone (**6**), ziza‐6(13)‐en‐3α‐ol (**11**), ziza‐6(13)‐enone (**9**), and 2‐*epi*‐ziza‐6(13)‐enone (**10**).

This was also what Belhassen et al. concluded in a recent study despite the fact that khusimone (**6**) does indeed display the typical woody–ambery vetiver profile.^[9]^ Using a combination of GC×GC/MS and GC olfactometry, they discussed ziza‐6(13)en‐3‐one (**9**) and 2‐*epi*‐ziza‐6(13)en‐3‐one (**10**) as potentially important odor vectors since they displayed higher flavor dilution factors than khusimone (**6**). They also attributed a typical vetiver note to ziza‐6(13)‐en‐3α‐ol (**11**),^[10]^ the corresponding (*R*,*R*)‐configured alcohol of compound **9**, but found it weaker. Different to patchouli (Clearwood) or sandalwood oil (Dreamwood),^[5]^ an industrially viable biotechnological process does not exist for vetiver oil, and would first require knowledge about its smelling principles. To make matters worse, there were even speculations about root bacteria being involved in the biogenesis of the components.^[11]^ So, independently from Belhassen et al., the smelling principle of vetiver oil was intensely sought after in the fragrance industry. A large‐scale distillation (800 g) of vetiver oil Haiti (Figure 2) with a 50 cm Sulzer column and then Spaltrohr system followed by separation of the aldehydes and ketones in the most fragrant fraction with the Girard P reagent and subsequent flash chromatographic fractionation indeed suggested the ketone **10** to be the most likely candidate for the principal odorant. However, due to the enormous complexity of the essential oil (Figure 2), of which only 155 components have been identified,[^6a^, ^8^] falsifications by high‐impact trace impurities could never be excluded. In addition, more easily accessible synthetic substructures did not smell of vetiver,^[12]^ and casted some doubt again on substances **9**–**11** being indeed the key vetiver odorant. Since the presence of an extremely intense odoriferous minor constituent is always possible and can be overlooked even by state‐of‐the‐art analytical techniques (Figure 2), the pure substances are required to reach a definitive conclusion about their olfactory importance. As for GC olfactometry, any effort to physically isolate the key odorant from the complex matrix even by preparative GC is prone to contaminations.

**Figure 2:**
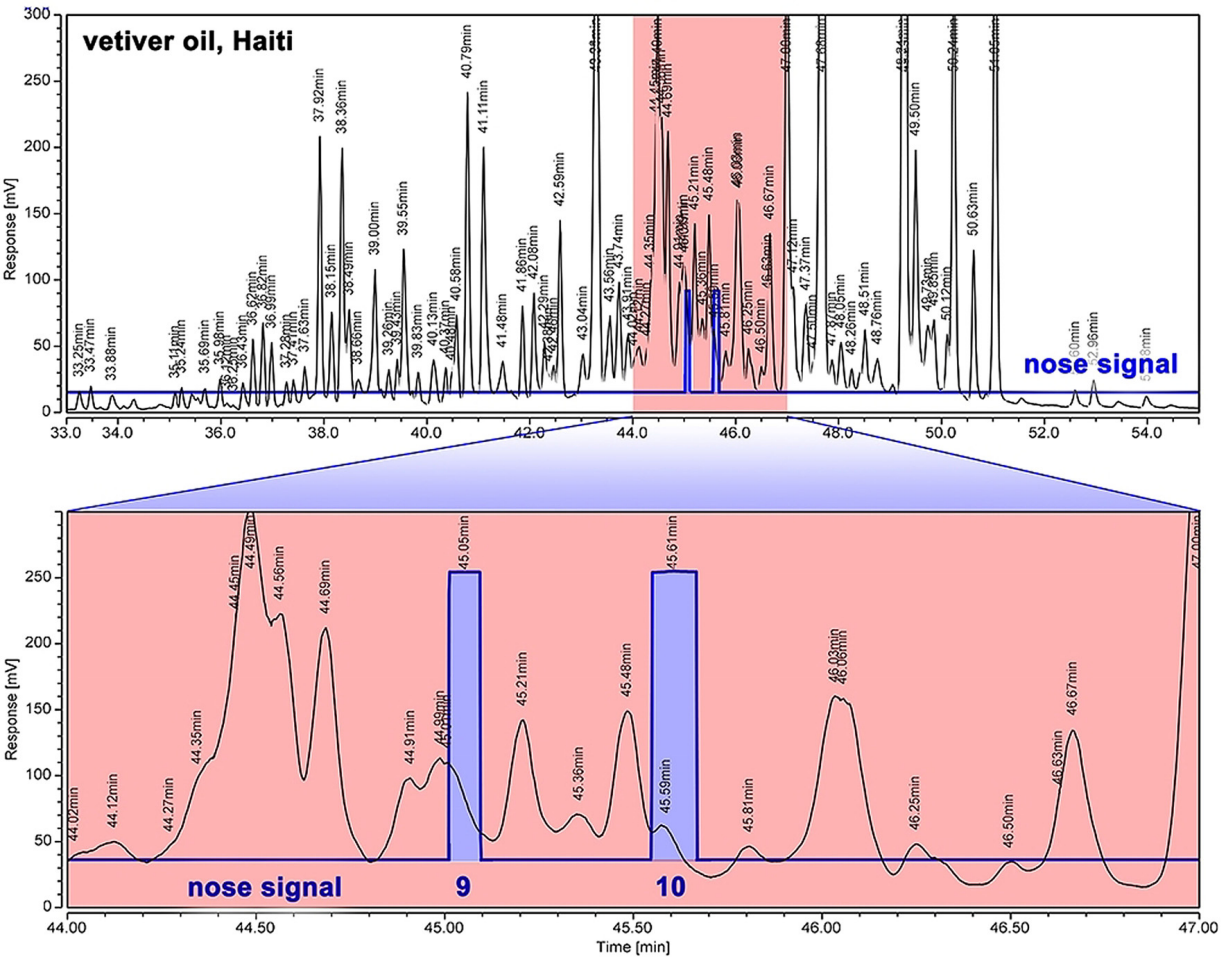
GC–Olfactometry of vetiver oil from Haiti. The overview GC trace (FID, upper part) demonstrates the enormous complexity of this natural essential oil, being overloaded with hundreds of individual peaks. In this jungle of peaks, the blue signal indicates when the typical transparent vetiver character is recognizable at the sniffing port. As was later verified by co‐injection, these indeed correspond to ziza‐6(13)‐enone (**9**) and 2‐*epi*‐ziza‐6(13)‐enone (**10**).

In light of these considerations, it became apparent that only a stereoselective chemical total synthesis could provide clarity. We therefore embarked upon the development of an enantioselective and diastereoselective route toward 2‐*epi*‐ziza‐6(13)en‐3‐one (**10**). In our synthesis design, the emphasis was placed on developing a route that would establish the correct (*R*)‐configuration at C‐8 early on, which is common to all the zizaenone natural products. The synthesis should be more flexible concerning the additional stereogenic centers, potentially enabling access to different zizaenone diastereomers for olfactory evaluation.

As delineated in Figure 3, for the key step in the construction of the unique carbon skeleton of 2‐*epi*‐ziza‐6(13)en‐3‐one (**10**), we designed an intramolecular Pauson–Khand cyclization of 1‐ethylidene‐3‐(2‐methyl‐3‐methylenepent‐4‐yn‐2‐yl)cyclopentane (**20**), which should afford the tricyclic ziza‐6(13)‐en‐3‐one **21**. Seeking to realize this synthetic blueprint, an enantioselective Mukaiyama–Michael reaction of 2‐cyclopentenone **12** with the readily accessible and commercially available silyl ketene acetal (SKA) of methyl isobutyrate appeared to be ideally suited to assemble enantiopure cyclopentane **15**,^[13]^ which in turn appeared to be a convenient precursor of the Pauson–Khand substrate **20**. Surprisingly, however, despite intense recent research on asymmetric, catalytic Mukaiyama–Michael‐type addition reactions, examples with 2‐cyclopentenone as electrophile are extremely rare, and the suggested substrate combination has even been entirely unprecedented.^[14]^

**Figure 3:**
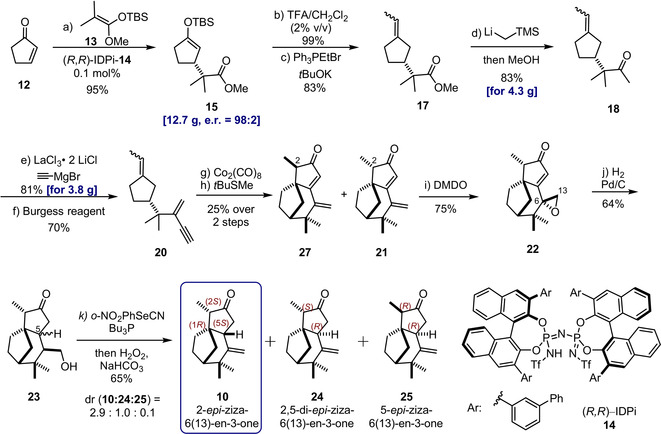
Enantioselective total synthesis of 2‐*epi*‐ziza‐6(13)‐en‐3‐one (**10**). Reagents and conditions: a) Cyclopentenone (**12**, 1 equiv.), SKA (**13**, 1.05 equiv), catalyst **14** (0.1 mol %), −78 °C, toluene, 95 %, er 98:2. b) **15** (1 equiv), TFA (1.05 equiv), DCM, 23 °C, 10 min, 99 %. c) Ethyltriphenylphosphonium bromide (1.2 equiv), potassium *tert*‐butoxide (1.2 equiv), THF, −60 °C, 1 h, then desilylated ketone (1 equiv), −60→23 °C, 83 %, *E*/*Z=*1:1.7. d) **17** (1.0 equiv), (trimethylsilyl)methyl lithium (2.5 equiv), pentane, 0 °C, then MeOH, 2 h, 83 %. e) LaCl_3_⋅2 LiCl (1.06 equiv), THF, 0 °C, then ethynylmagnesium chloride (1.7 equiv), 81 %. f) Burgess reagent (2.3 equiv), hexane/toluene (9:1), 60 °C, 19 h, 70 %. g) Dicobalt octacarbonyl (1.3 equiv), diethyl ether, 23 °C. h) *tert*‐Butyl (methyl)sulfane (3.5 equiv, based on **20**), benzene, 90 °C, 19 h, 25 % over 2 steps, diastereoisomers (dr 1.7:1 at C‐2) were separated by preparative HPLC. i) DMDO (excess), DCM, 0 °C, 75 %, with dr 9:1 at C‐6 in favor of **22**. j) Palladium on activated charcoal (3 equiv), H_2_, ethyl acetate, 23 °C, 64 %, dr 2:1 at C‐5. k) **23** (1 equiv), 1‐nitro‐2‐selenocyanatobenzene (1.28 equiv), tributylphosphine (2.5 equiv), THF, 23 °C, 1 h, NaHCO_3_ (30 equiv), H_2_O_2_ (30 % aq, 30 equiv), 23 °C, 4 h, 65 %, dr(**10**/**24**/**25**) = 2.9:1:0.1, diastereoisomers were separated by preparative HPLC. SKA, silyl ketene acetal; TFA, trifluoroacetic acid; DCM, dichloromethane; THF, tetrahydrofuran; Burgess reagent, methyl *N*‐(triethylammoniumsulfonyl)carbamate; DMDO, dimethyldioxirane; er, enantiomeric ratios were measured by HPLC on a chiral stationary phase; dr, diastereomeric ratios were determined either by NMR or GC.

Based on the concept of asymmetric counteranion‐directed silylium Lewis acid catalysis (*Si*‐ACDC), previously developed in our laboratory,^[15]^ we suspected that our imidodiphosphorimidate (IDPi) catalyst class could enable the desired transformation.^[16]^ Indeed, after some design and experimentation, which will be published separately, we could establish a method to access the desired substituted cyclopentenone **15**. Thus, upon treating 0.1 mol % of imidodiphosphorimidate (IDPi) catalyst **14** with 2‐cyclopentenone and silyl ketene acetal **13**, the desired (*R*)‐configured silyl enol ether **15** was obtained on a multigram scale in 95 % yield with an excellent enantiomeric ratio of 98:2. Protodesilylation with trifluoroacetic acid and Wittig reaction with ethyltriphenylphosphonium bromide provided the olefin **17** in 83 % yield, with an *E*/*Z* ratio of 1:1.7.^[17]^ This material was directly converted into the corresponding methyl ketone **18** in 83 % yield on a multigram scale by adding trimethylsilyl methyllithium, followed by protodesilylation.^[18]^ Subsequent addition of ethynyl magnesium bromide, under conditions established by Knochel et al. to counteract steric hindrance and reactivity issues, led to the corresponding isolated alcohol in 81 % yield.^[19]^ The dehydration of this intermediate turned out to be more challenging than expected and could only be realized by utilizing the mild and efficient Burgess reagent,^[20]^ providing the desired product **20**, the precursor of the central Pauson–Khand reaction, in 70 % yield. As expected, the sterically highly demanding intramolecular Pauson–Khand [2+2+1] cycloaddition became a critical bottleneck of our synthetic sequence. Several well‐established Pauson–Khand protocols failed to furnish even traces of the desired product **21**.^[21]^ After surveying various options, a stepwise strategy was chosen in which the 18‐electron dicobaltatetrahedrane complex was first prepared and isolated by reacting alkyne **20** with dicobalt octacarbonyl. Its subsequent cyclization was promoted by *tert*‐butyl(methyl)sulfide in refluxing benzene to afford the desired product **21** and its separable C‐2‐epimer **27** (cf. Supp. Info.) in 25 % yield. While the overall yield of this transformation remained unsatisfactory, despite our best efforts, the powerful Pauson–Khand cyclization established the entire carbon skeleton of the natural product, inducing all three rings, and the critical quaternary stereogenic center in its correct configuration.

With enone **21** in hand, we felt in a very good position to accomplish the last challenge of our synthesis, consisting of the selective reduction of the cyclopentenone double bond. Even though this transformation seemed simple, it turned out to be the most difficult one of the entire sequence. Under various reduction conditions (e.g. Stryker's Reagent,^[22]^ SmI_2_,^[23]^ Hantzsch ester,^[24]^ Wilkinson's reagent,^[25]^ etc., see Supp. Info.), rather than the desired 1,4‐reduction product, only products from the corresponding 1,6‐reduction of the exocyclic double bond were obtained. Accordingly, an indirect method had to be developed. Indeed, by first epoxidizing the much more reactive exocyclic olefin with freshly prepared dimethyldioxirane (DMDO), epoxide **22** was smoothly obtained in 75 % yield (dr 9:1 at C‐6).^[26]^ The constitution and stereochemistry of this product were confirmed by X‐ray crystallography. Subsequent hydrogenation of the enone double bond in the presence of Pd/C proceeded readily, along with a hydrogenative ring‐opening of the epoxide to provide alcohol **23** in 64 % yield (dr 2:1, at C‐5). We attributed the moderate diastereofacial differentiation of this conjugate reduction to the negligible steric effect of the equatorial methylene group of the epoxide at C‐13. The subsequent dehydration of primary alcohol **23** was realized via a Grieco elimination,^[27]^ resulting in three diastereoisomers in 65 % total yield with a diastereomeric ratio of 2.9:1:0.1 in favor of target **10**. The obtained traces of product **25** presumably result from the isomerization of the minor isomer **24** under the basic elimination conditions.

The olfactory properties and odor thresholds^[28]^ of the four (1*R*)‐configured ziza‐6(13)‐en‐3‐one stereoisomers are summarized in Figure 4. While the ziza‐6(13)‐en‐3‐one (**9**) possesses a clean, fresh, transparent woody–ambery vetiver character with an odor threshold of 0.13 ng L^−1^ air, 2‐*epi*‐ziza‐6(13)‐en‐3‐one (**10**) featured a much more pronounced and typical vetiver character along with an accentuated dry and distinctly transparent woody–ambery note. This odor character is reminiscent of the popular perfumery material Iso E Super with a related transparent woody–ambery note in which arborone (**26**)^[29]^ is claimed to cause a quasi‐pheromone‐like attraction.^[30]^ This odor association was explained by a superposition analysis (75 % steric, 25 % electronic, Discovery Studio^[31]^), a method preferentially used to evaluate the most substantial common three‐dimensional substructure of a set of molecules that bind to the same receptor during drug or odorant discovery.^[32]^ As is depicted in the overlay analysis in Figure 4, 2‐*epi*‐ziza‐6(13)‐en‐3‐one (**10**) does superimpose surprisingly well on arborone (**26**, overlay similarity/**26**=0.87), which implies a substantial similarity of the smelling principles of vetiver oil and Iso E Super. As also evident from Figure 4, the odorless antipode *ent*‐**10** does not superimpose that well, especially concerning the crucial hydrophic *gem*‐dimethyl group of **26**.

**Figure 4:**
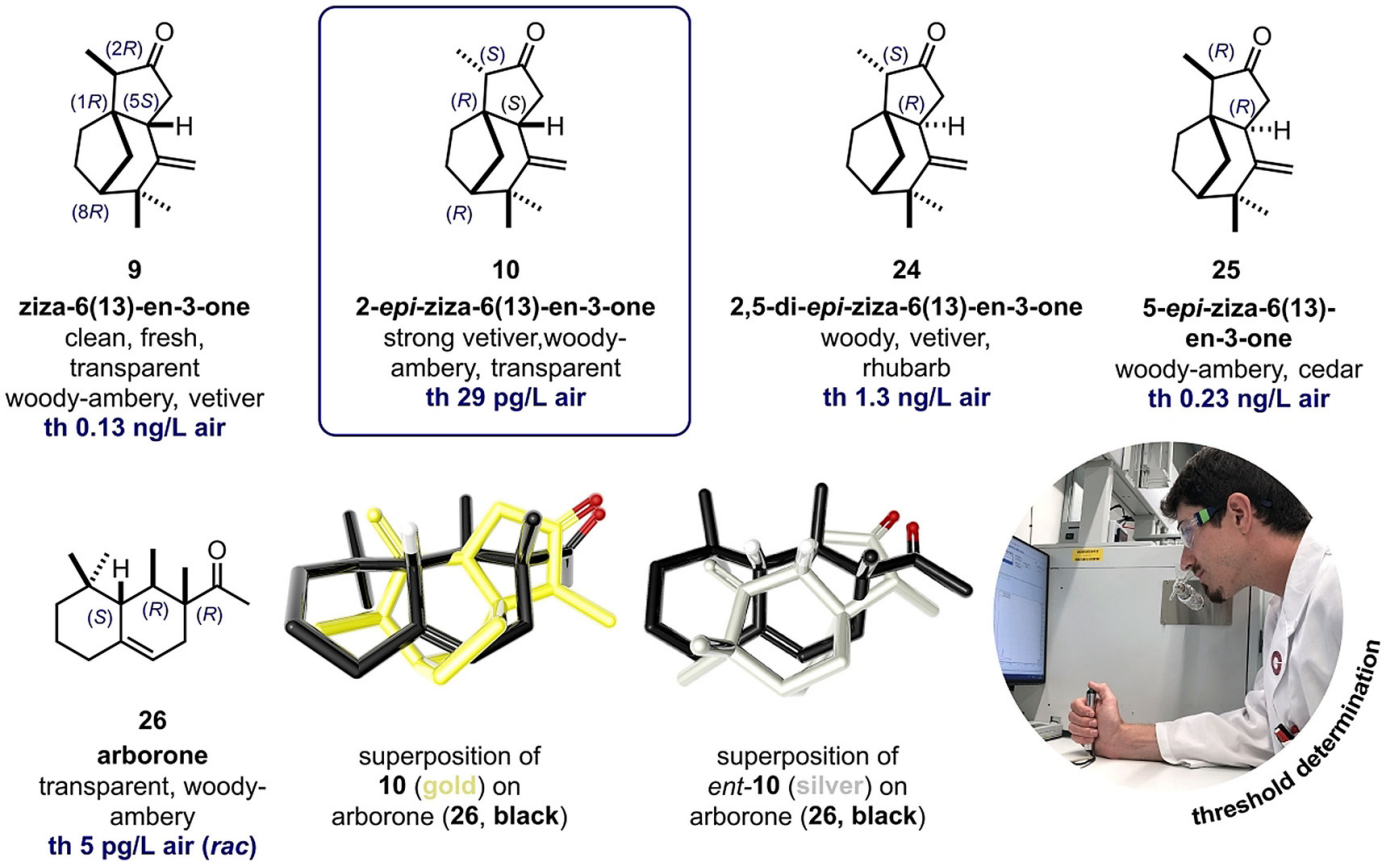
Olfactory properties of the (1*R*)‐configured ziza‐6(13)‐en‐3‐one stereoisomers **9**, **10**, **24**, and **25**; the enantiomeric antipodes *ent*‐**9**, *ent*‐**10**, *ent*‐**24**, and *ent*‐**25** are all odorless. Also shown is arborone (**26**), the smelling principle of the commercial perfumery ingredient Iso E Super, a superposition (75 % electronic, 25 % steric) of 2‐*epi*‐ziza‐6(13)‐en‐3‐one (**10**) on **26** explaining its transparent woody–ambery odor profile in comparison to the odorless enantiomer *ent*‐**10**, and the setup for the determination of the odor thresholds by GC–olfactometry.

Besides, comparing the naturally occurring ziza‐6(13)‐en‐3‐one (**9**) and 2‐*epi*‐ziza‐6(13)‐en‐3‐one (**10**), the synthetic diastereomers 2,5‐di‐*epi*‐ziza‐6(13)‐en‐3‐one (**24**) and 5‐*epi*‐ziza‐6(13)‐en‐3‐one (**25**) also possess woody–ambery odors. However, neither **24**, with more pronounced hesperidic rhubarb facets and a 1.3 ng L^−1^ threshold, nor **25**, with a more cedarwood character and a 0.23 ng L^−1^ threshold in air, are stronger or more characteristic than the natural vetiver odor vector **10**, and besides, are also not detectable in vetiver oil.

In conclusion, the described enantioselective total synthesis of 2‐*epi*‐ziza‐6(13)‐en‐3‐one (**10**) represents a successful departure from the traditional separation strategy to disclose a smelling principle. The eleven‐step synthesis lays the foundation for an unprecedented enantioselective access to zizaenes. Threshold evaluation reveals the enantiopure ketone **10** to be the key contributor to the typical transparent woody–ambery vetiver note, being over 150 times more potent in odor threshold than khusimone (**6**), which was previously deemed the smelling principle of vetiver. Moreover, the excellent stereochemical superposition of 2‐*epi*‐ziza‐6(13)‐en‐3‐one (**10**) on arborone (**26**) could well explain the almost magic attraction that vetiver oil exerts on humans, and why this surprisingly resembles the irresistible pull of Iso E Super—an effect still not well‐understood physiologically but now tangible on the molecular level.

## Conflict of interest

The authors declare the following competing financial interest(s): A patent, WO2017037141 (A1), has been filed by the MPI für Kohlenforschung covering the IDPi catalyst class and their applications in asymmetric synthesis. S.J., S.D., and P.K. are employees of Givaudan S.A., a commercial producer of perfumes and fragrance ingredients.

## Supporting information

As a service to our authors and readers, this journal provides supporting information supplied by the authors. Such materials are peer reviewed and may be re‐organized for online delivery, but are not copy‐edited or typeset. Technical support issues arising from supporting information (other than missing files) should be addressed to the authors.

Supplementary
