# Supplementary material for: The Smelling Principle of Vetiver Oil, Unveiled by Chemical Synthesis
Source: Angew Chem Int Ed Engl. 2021 Feb 9;60(11):5666–72. doi: 10.1002/anie.202014609 (PMC7986879; doi:10.1002/anie.202014609)
Supplement: Supplementary file 1 — Supplementary [file ANIE-60-5666-s001.pdf]

Angewandte Chemie
Eine Zeitschrift der Gesellschaft Deutscher Chemiker

Supporting Information

The Smelling Principle of Vetiver Oil, Unveiled by Chemical Synthesis

Jie Ouyang, Hanyong Bae, Samuel Jordi, Quang Minh Dao, Sandro Dossenbach, Stefanie Dehn, Julia B. Lingnau, Chandra Kanta De, Philip Kraft, and Benjamin List**

anie_202014609_sm_miscellaneous_information.pdf

Contents

General information 3
GC–Olfactometry of vetiver oil from Haiti 4
Synthesis and Characterization of Catalyst 5
(*R*)- 3,3'-Di([1,1'-biphenyl]-3-yl)-[1,1'-binaphthalene]-2,2'-diol 5
(*R*, *R*)-Imidodiphosphorimidate (*R*, *R*-IDPi, **14**) 6
The optimization of Mukaiyama–Michael addition 8
The synthesis of 2-*epi*-ziza-6(13)-en-3-one (10) 9
Synthesis of compound (–)-**15** 9
Synthesis of compound (–)-**16** 10
Synthesis of compound (–)-**17** 11
Synthesis of compound (–)-**18** 12
Synthesis of compound (–)-**19** 13
Synthesis of compound (–)-**20** 14
Synthesis of compound (–)-**21** 15
Synthesis of compound (–)-**22** 17
Synthesis of compound (–)-**23** 18
Synthesis of compound (–)-**10** 19
Elucidation of the absolute configuration of the Mukaiyama–Michael addition product 21
Attempts towards selective 1, 4-reduction of dienone **21** 22
References 23
Single-Crystal X-ray Diffraction Analysis of epoxide **22** 23
NMR Spectra 26
HPLC and GC trace 72
GC odour threshold determination – Chromatography & Olfactometry 76

General information

Unless otherwise stated, all reagents were purchased from commercial suppliers and used without further purification. Aldehydes were distilled and stored under Ar before use. All solvents used in the reactions were distilled from appropriate drying agents before use. Reactions were monitored by thin-layer chromatography (TLC) on silica gel pre-coated plastic sheets (0.2 mm, Macherey-Nagel) or glass plates (SIL G-25 UV254, 0.25 mm, Macherey-Nagel). Visualization was accomplished by irradiation with UV light at 254 nm and *p*-anisaldehyde (PAA) stain. PAA stain: to absolute EtOH (135 mL) was added conc. sulfuric acid (5 mL), glacial acetic acid (1.5 mL) and *p*-anisaldehyde (3.7 mL). Column chromatography was performed on Merck silica gel (60, particle size 0.040–0.063 mm). NMR spectra were recorded on Bruker AV-500, Bruker AV-400, or Bruker AV-300 spectrometer in deuterated solvents. Proton chemical shifts are reported in ppm (δ) relative to tetramethylsilane (TMS) with the solvent resonance employed as the internal standard ($CDCl_3$ δ 7.26 ppm; CD_2Cl_2 δ 5.32 ppm). Data are reported as follows: chemical shift, multiplicity (s = singlet, d = doublet, t = triplet, q = quartet, p = pentet, sext = sextet, h = heptet, m = multiplet, br = broad), coupling constants (Hz) and integration. ^{13}C chemical shifts are reported in ppm from tetramethylsilane (TMS) with the solvent resonance as the internal standard ($CDCl_3$ δ 77.16 ppm; CD_2Cl_2 δ 53.84 ppm). High-resolution mass spectra were determined on a Bruker APEX III FTMS (7 T magnet). All reported yields, unless otherwise specified, refer to spectroscopically and chromatographically pure compounds. Optical rotations were determined with Autopol IV polarimeter (Rudolph Research Analytical) at 589 nm and 25 °C. Data are reported as follows: $[\alpha]_{\lambda}^{temp}$, concentration (*c*; g/100 mL), and solvents. Enantiomeric ratios (e.r.) were determined by GC or HPLC analysis employing a chiral stationary phase column specified in the individual experiment, by comparing the samples with the appropriate racemic mixtures. Diastereomeric ratios (d.r.) were determined by ^{1}H NMR spectra of the crude reaction mixtures.

GC–Olfactometry of vetiver oil from Haiti

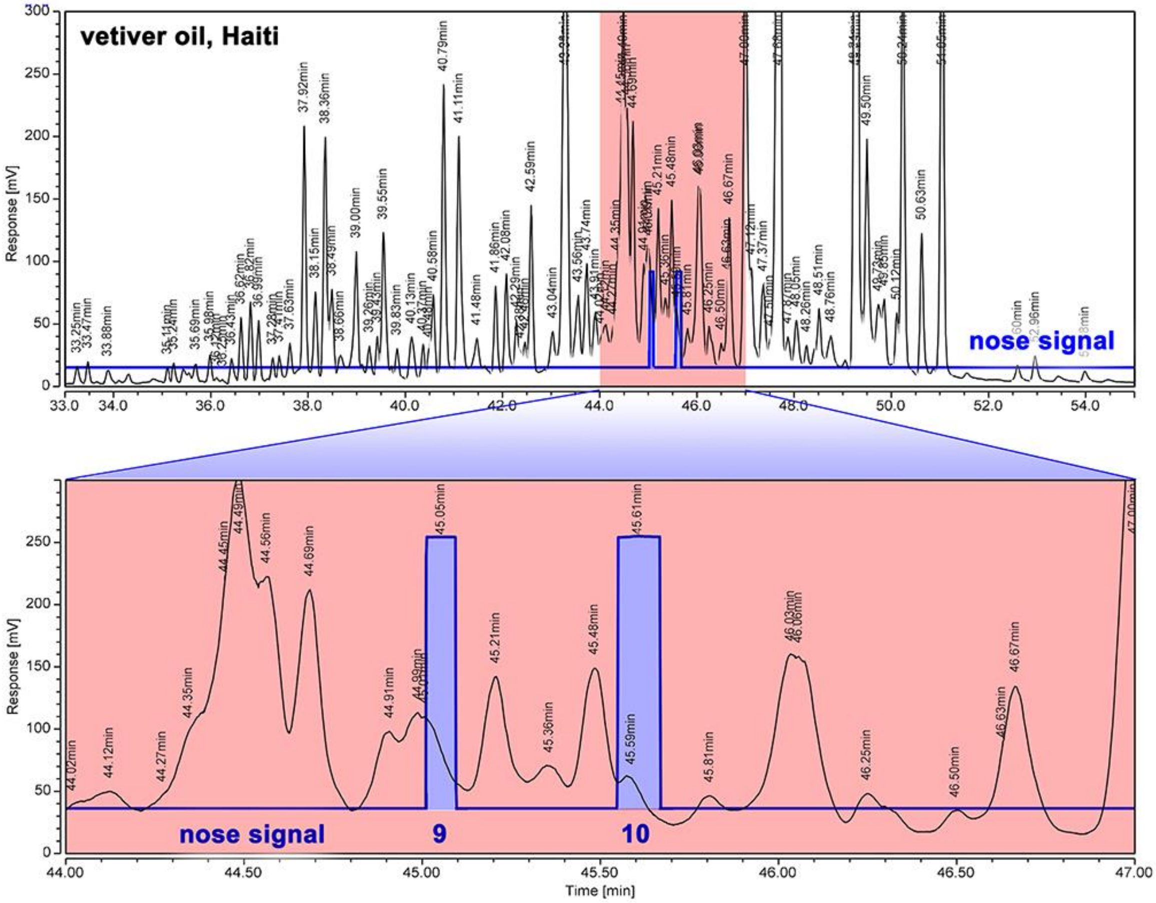

Fig. S1. GC–Olfactometry of vetiver oil from Haiti

GC sniffing assessment was performed on a Trace GC Ultra gas chromatograph (Thermo Scientific) equipped with a CombiPAL autosampler (CTC Analytics), a cool on-column injector, a flame ionization detector (FID), an olfactory detection port (ODP, Givaudan in-house construction) and a DB-5 capillary column (Agilent, 30m length, 0.32mm I.D., 0.5µm film thickness). Working conditions were as follows: 1µl injection volume (1000ng/µl stock solution in MtBE), He as carrier gas, constant flow 2.5ml/min, FID base temperature 270°C, air 350ml/min, H2 35ml/min, N2 make-up 30ml/min with the effluent split 1:1 to the FID and ODP. The temperature was programmed from 40°C with 2-min hold to 250°C at 3°C/min with a 20-min final temperature hold.

Synthesis and Characterization of Catalyst

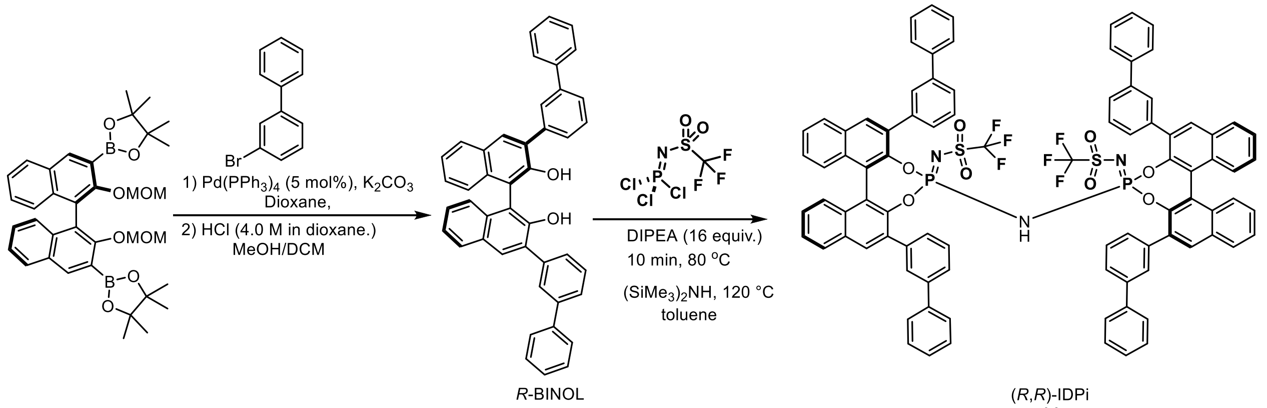

Fig. S2. Preparation of catalyst **14**

(*R*)- 3,3'-Di([1,1'-biphenyl]-3-yl)-[1,1'-binaphthalene]-2,2'-diol

To a flame dried two-neck round-bottom flask with a condenser was added (*R*)-2,2'-(2,2'-bis(methoxymethoxy)-1,1'binaphthyl-3,3'-diyl)bis(4,4,-5,5-tetramethyl-1,3,2-dioxaborolane) (15.7 g, 24 mmol, 1.0 equiv), 3-bromo-1,1'-biphenyl (24 g, 100 mmol, 4 equiv), tetrakis(triphenylphosphine)palladium (1.4 g, 1.2 mmol, 0.05 equiv) and barium hydroxide octahydrate (22 g, 64 mmol, 2.9 equiv.). After degassing the reaction mixture with argon for 20 min, 1, 4-dioxane (150 ml) and degassed H_2O (43 mL) were sequentially added. The mixture was then heated to 120 °C and stirred at that temperature overnight. After cooling the reaction to room temperature, the mixture was filtered through a pad of celite, washing with DCM (3 x 100 mL). The layers were separated, and the aqueous was extracted with DCM (2 x 50 mL). The combined organic phases were washed with brine (200 mL), dried over anhydrous $MgSO_4$, and filtered. The solvent was removed under vacuum to afford a light yellow solid, which was directly used for the next step. Subsequently, the solid was dissolved in DCM (70 mL), MeOH (55 mL) and a solution of HCl (4 M in dioxane, 48 mL) was added to this mixture at room temperature. This mixture was heated to 80 °C and stirred overnight until the starting material was consumed (as monitored by TLC analysis). The reaction was then cooled to room temperature and quenched with water (100 mL). The resulting mixture was extracted with DCM (2 x 100 mL), then the layers were separated. The aqueous was extracted with DCM (100 mL), and the combined organic layers were dried over anhydrous $MgSO_4$. Following filtration of the suspension through celite, the volatiles were removed under reduced pressure. The resulting residue was purified by column chromatography (EtOAc: isohexane = 1:50 to 1: 9) to afford (*R*)-3,3'-di([1,1'-biphenyl]-3-yl)-[1,1'-binaphthalene]-2,2'-diol (13.4 g, 22.8 mmol, 95%) as a light yellow solid.

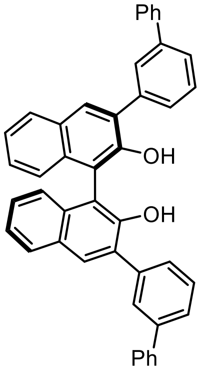

$[\alpha]_D^{25}$ = 32.6 (c = 0.42, CH_2Cl_2)

^{1}H NMR (500 MHz, $CDCl_3$) δ 7.98 (s, 2H), 7.88 (d, J = 1.8 Hz, 2H), 7.83 (d, J = 8.1 Hz, 2H), 7.62 (dt, J = 7.7, 1.5 Hz, 2H), 7.58 – 7.51 (m, 6H), 7.45 (t, J = 7.7 Hz, 2H), 7.34 (dd, J = 8.3, 7.0 Hz, 4H), 7.29 (ddd, J = 8.1, 6.7, 1.3 Hz, 2H), 7.27 – 7.20 (m, 4H), 7.19 – 7.14 (m, 2H), 5.32 (s, 2H).

^{13}C NMR (125 MHz, $CDCl_3$) δ 150.3, 141.7, 141.2, 138.1, 133.2, 131.6, 130.7, 129.6, 129.0, 128.9, 128.7, 128.6, 127.6, 127.5, 127.4, 126.7, 124.5, 124.4, 112.6.

HRMS (ESI^-) (*m*/*z*): $[M–H]^-$ calculated for $C_{44}H_{29}O_2$: 589.217305; found 589.217260.

(*R*, *R*)-Imidodiphosphorimidate (*R*, *R*-IDPi, 14)

To a flame dried Schlenk flask was charged (*R*)- 3,3'-di([1,1'-biphenyl]-3-yl)-[1,1'-binaphthalene]-2,2'-diol (500 mg, 0.85 mmol, 2.1 equiv.) under Ar, then dry toluene (3 mL) was added. The solution was flushed with Ar until the solvent was removed, and this procedure was repeated twice. The solid was dried under high vacuum at 50 °C for 1 h. To this flask was added dry toluene (15 mL) under Ar, and the mixture was heated to 80 °C to dissolve the solid, then *N*, *N*-diisopropylethylamine (1.1 mL, 6.4 mmol, 16 equiv.) was added, followed by the addition of *N*-Tf trichlorophosphazene (240 mg, 0.85 mmol, 2.1 equiv.)[1]. The solution was stirred for 10 min, and then hexamethyldisilazane (84 µL, 0.4 mmol, 1.0 equiv.) was added. The mixture was heated to 120 °C for 19 h. The reaction mixture was cooled to room temperature, and DCM (10 mL) was added to dilute the solution, quenched with HCl (1 M, aq., 15 mL). The layers were separated, and the aqueous phase was further extracted with DCM (2 x 10 mL). The combined organic layers were dried over anhydrous $MgSO_4$, filtered. The solvent was removed under vacuum to afford a yellow mixture, which was purified by column chromatography to furnish a light yellow solid. The resulting solid was dissolved in DCM (10 mL) and to this solution was added HCl (6 M aq., 10 mL). The mixture was vigorously stirred for 15 min, then the organic layer was separated, and the aqueous was extracted with DCM (2 x 5 mL). The combined organic solvent was removed in vacuo, and the resulting solid was placed under the higher vacuum at 25 °C to afford a dry IDPi **14** catalyst (450 mg, 73%).

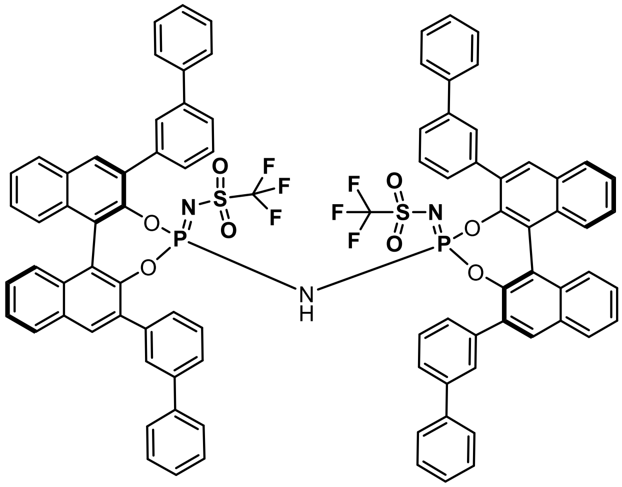

$[\alpha]_D^{25}$ = –269.1 (c = 0.11, CH_2Cl_2)

^{1}H NMR (500 MHz, CD_2Cl_2) δ 8.08 (s, 2H), 7.93 (dd, J = 11.3, 8.2 Hz, 4H), 7.75 (t, J = 7.5 Hz, 2H), 7.67 (s, 2H), 7.57 – 7.44 (m, 8H), 7.43 – 7.37 (m, 4H), 7.35 – 7.28 (m, 10H), 7.27 – 7.20 (m, 8H), 7.19 – 7.10 (m, 8H), 7.02 – 6.84 (m, 6H), 6.30 (s, 2H).

^{13}C NMR (125 MHz, CD_2Cl_2) δ 141.9, 141.1, 140.3, 136.0, 133.4, 132.9, 132.1, 131.9, 131.8, 131.7, 131.7, 131.1, 129.0, 128.9, 128.6, 128.6, 128.4, 128.3, 128.2, 128.1, 127.9, 127.6, 127.3, 127.3, 127.2, 127.2, 126.8, 126.7, 126.6, 126.4, 123.3, 121.8.

^{19}F NMR (470 MHz, CD_2Cl_2) δ. –78.76 (s, 6F).

^{31}P NMR (202 MHz, CD_2Cl_2) δ. –16.92.

HRMS (ESI) (*m/z*): $[M–H]^-$ calcd. for $C_{90}H_{56}F_6N_3O_8P_2S_2$: 1546.289386; found: 1546.289010.

The optimization of Mukaiyama–Michael addition

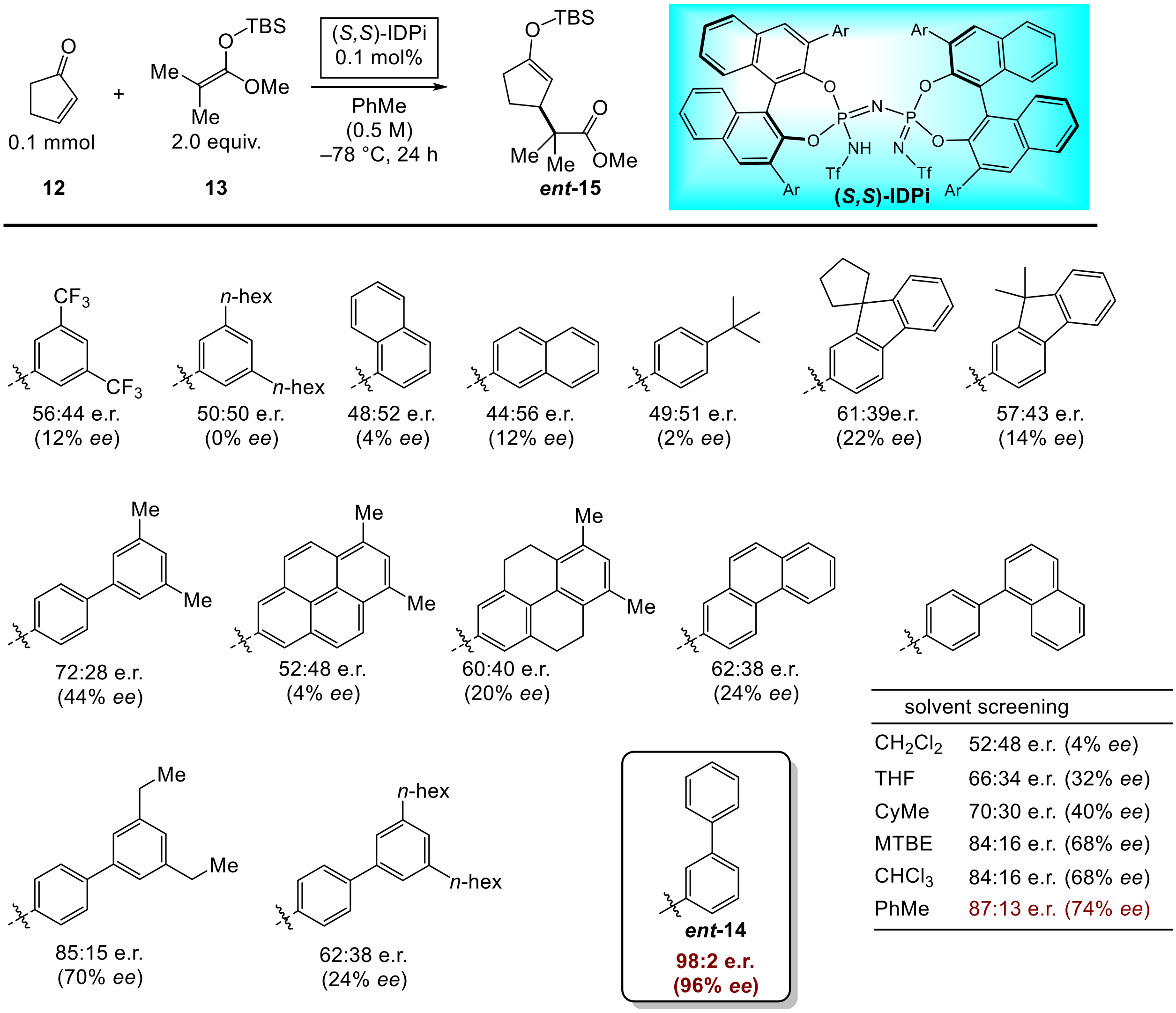

Fig. S3. The identification of the optimal catalyst for the asymmetric Mukaiyama–Michael addition

Based on the concept of asymmetric counteranion-directed silylium Lewis acid catalysis (*Si*-ACDC), a variety of (*S*, *S*)-IDPi catalysts developed in our lab were screened. After surveying a variety of options, a condition with the catalyst *ent*-**14** featuring an *m*-biphenyl as a substitution on 3, 3'- position of BINOL, in toluene, under –78 °C, was able to furnish the desired compound *ent*-**15** with an enantioselective ratio of 98:2.

The synthesis of 2-*epi*-ziza-6(13)-en-3-one (10)

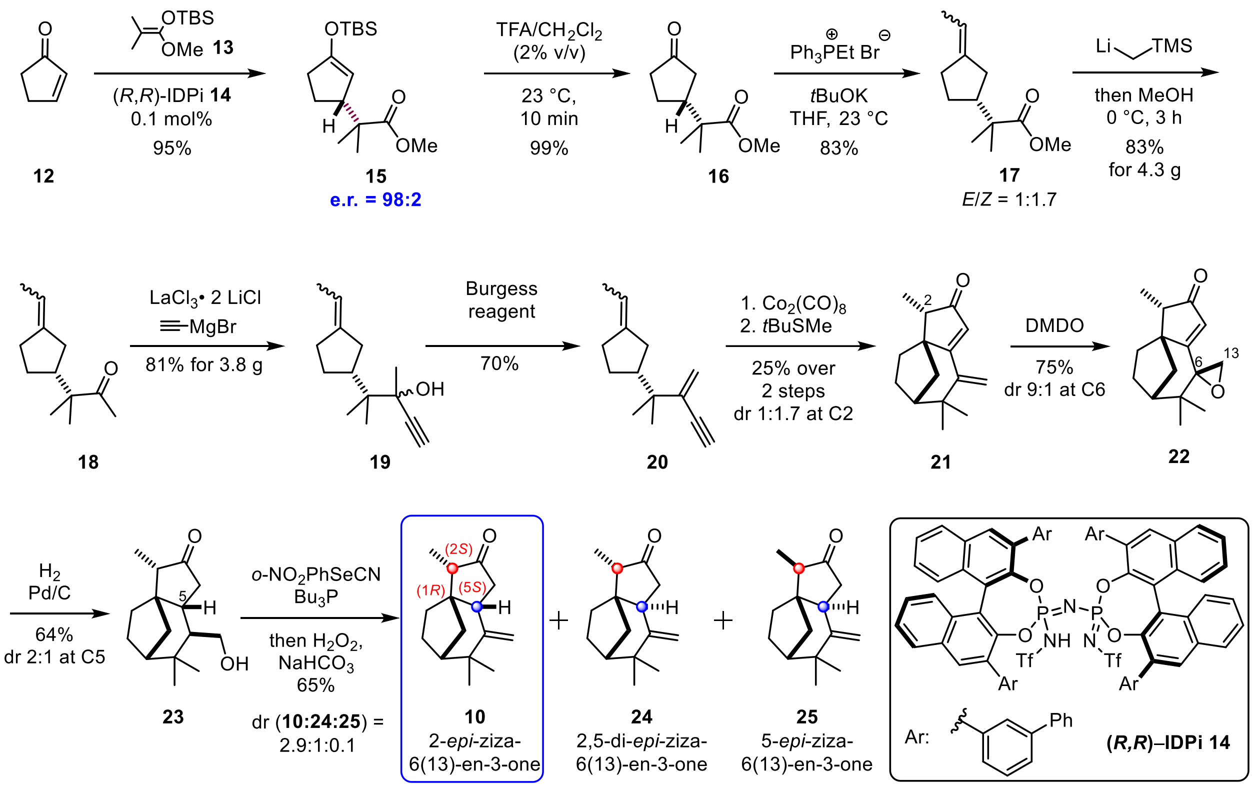

Synthesis of compound (–)-15

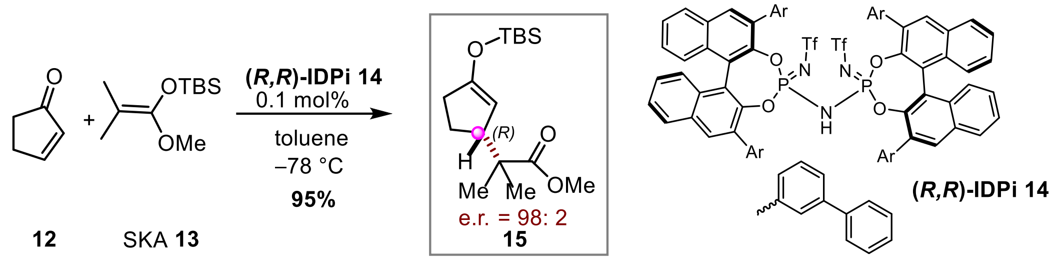

To a flame-dried Schlenk flask was added freshly distilled cyclopentenone **12** (3.7 g, 45 mmol, 1.0 equiv.) under Ar, then followed by the addition of dry toluene (45 mL). The flask was cooled down to -78 °C in a dry ice bath. The catalyst **14** (70 mg, 0.045 mmol, 0.001 equiv.) was added in one portion and then followed by the slow addition of the freshly prepared *tert*-butyl((1-methoxy-2-methylprop-1-en-1-yl)oxy)dimethylsilane **13**[2] (10.3 g, 47.5 mmol, 1.05 equiv.) to this cooled mixture. After the full consumption of cyclopentenone confirmed by TLC, Et_3N (14 µL, 0.002 equiv) was added to quench the reaction, and the resulting mixture was quickly loaded on a pre-cooled silica-gel column (the column was washed with dry ice-cooled pentane) to carry out the chromatography purification (Et_2O: Pentane = 0 to 1:10 gradually) to afford **15** as a colorless oil (12.7 g, 95%).

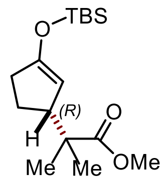

15

[α]$_D^{25}$ = 9.5 (c = 0.42, CH_2Cl_2, 98:2 e.r.)

^{1}H NMR (500 MHz, $CDCl_3$) δ 4.51 (s, 1H), 3.65 (s, 3H), 2.97 (ddd, *J* = 8.3, 4.9, 2.3 Hz, 1H), 2.24–2.15 (m, 2H), 2.04–1.81 (m, 1H), 1.68–1.46 (m, 1H), 1.10 (s, 3H), 1.07 (s, 3H), 0.91 (d, *J*=1.0 Hz, 9H), 0.15 (s, 6H).

^{13}C NMR (126 MHz, $CDCl_3$) δ 178.52, 156.76, 103.32, 51.71, 50.25, 46.00, 33.56, 25.81, 23.52, 22.91, 21.38, 18.25, -4.37, -4.53.

HRMS (CI) (*m/z*): $[M+H]^+$ calcd. for $C_{16}H_{31}O_3Si_1$: 299.203699; found: 299.203700.

E.r. 98:2

The enantiomeric purity was measured by HPLC analysis by comparison with a racemate using the following parameters: AD-3 column: acetonitrile: water = 50:50, flow rate 1 mL/min, t_{major} = 18.8 min, t_{minor} = 20.9 min.

Synthesis of compound (–)-16

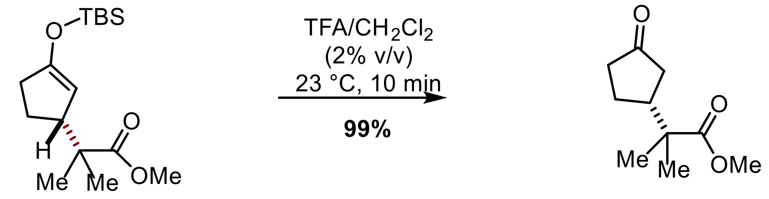

15 16

To a solution of silyl enol ether **15** (13.5 g, 45 mmol) in dry DCM (180 mL) was slowly added trifluoroacetic acid (3.63 mL, 47 mmol, 1.05 equiv.) at 23°C. After 10 min, the starting material was consumed, as confirmation of TLC analysis. To the mixture was added the $NaHCO_3$ (sat. aq., 100 mL), the aqueous layer was separated and extracted with Et_2O (3 x 50 mL). The combined organic layers were dried over anhydrous $MgSO_4$, filtered, and the solvents were removed in *vacuo*. Purification by column chromatography afforded **16** as a colorless oil (8.2 g, 99%).

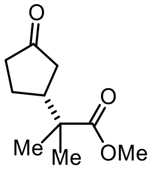

16

[α]$_D^{25}$ = 86 (c = 0.6, Et_2O)

^{1}H NMR (500 MHz, $CDCl_3$) δ 3.68 (s, 3H), 2.50 – 2.39 (m, 1H), 2.33 (ddt, *J* = 18.7, 8.5, 1.8 Hz, 1H), 2.29 – 2.22 (m, 1H), 2.17 (dddd, *J* = 18.7, 11.7, 8.8, 1.3 Hz, 1H), 2.08 – 1.95 (m, 2H), 1.64 (qd, *J* = 11.9, 8.5 Hz, 1H), 1.20 (d, *J* = 4.4 Hz, 6H).

^{13}C NMR (125 MHz, $CDCl_3$) δ 218.4, 177.4, 52.0, 45.2, 44.0, 40.6, 39.0, 24.6, 22.9, 22.8.

HRMS (CI) (*m/z*): $[M+H]^+$ calcd. for $C_{10}H_{17}O_3$: 185.11722; found: 185.11705.

Synthesis of compound (–)-17

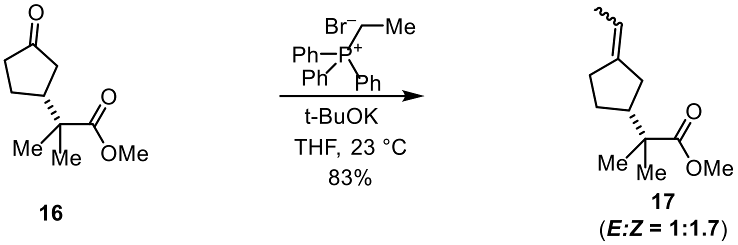

Ethyltriphenylphosphonium bromide (19.2 g, 51.8 mmol, 1.2 equiv.) was dissolved in dry THF (67 mL), and then the flask was cooled down –60 °C (dry ice/*i*PrOH). To this solution, a solution of potassium *tert*-butoxide (5.8 g, 51.8 mmol, 1.2 equiv.) in THF (60 mL) was slowly added under Ar. The resulting dark orange mixture was stirred for 1 h at –60 °C. A solution of methyl 2-methyl-2-(3-oxocyclopentyl) propanoate **16** (7.95 g, 43 mmol, 1 equiv.) in dry THF (20 mL) was then added slowly at –60 °C. The mixture turned to yellow-orange, and the cooling bath was removed after 15 min. The reaction mixture was stirred for 2 h at room temperature until the **16** was consumed (monitored by TLC). The resulting mixture was poured into 100 mL ice-water and was stirred vigorously for 10 min. Then the mixture was extracted with Et_2O (150 ml). The layers were separated, and the aqueous was extracted with Et_2O (3 x 50 ml). The organic layers were combined, dried over anhydrous $MgSO_4$, filtered, and the solvents were removed under reduced pressure. The residue was purified by column chromatography to afford an inseparable *Z/E* mixture **17** (7 g, 83%, *E: Z* = 1:1.7). *Note*: In order to obtain spectrums of pure isomers, a preparative HPLC separation was carried out to separate the mixture.

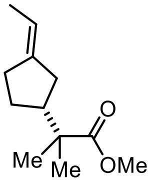

17-*E*

$[\alpha]_D^{25}$ = 32.6 (c = 0.7, $CDCl_3$)

^{1}H NMR (500 MHz, $CDCl_3$) δ 5.27 (tdp, *J* = 6.6, 4.2, 2.1 Hz, 1H), 3.65 (s, 3H), 2.41 – 2.27 (m, 1H), 2.25 – 2.18 (m, 1H), 2.16 – 1.95 (m, 3H), 1.72 (dddd, *J* = 9.7, 7.9, 4.7, 1.7 Hz, 1H), 1.54 (ddd, *J* = 6.7, 2.8, 1.5 Hz, 3H), 1.45 – 1.31 (m, 1H), 1.15 (d, *J* = 3.7 Hz, 6H).

^{13}C NMR (125 MHz, $CDCl_3$) δ 178.4, 142.4, 114.9, 51.7, 48.2, 43.9, 35.0, 29.9, 28.3, 27.3, 22.9, 14.5.

HRMS (ESI) (*m/z*): $[M+Na]^+$ calcd. for $C_{12}H_{20}O_2Na_1$: 219.135549; found: 219.135760.

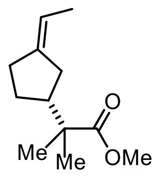

17-*Z*

[α]D[25] = 21 (c = 0.7, Et_2O)

^{1}H NMR (400 MHz, $CDCl_3$) δ 5.25 (qm, *J* = 6.9 Hz, 1H), 3.66 (s, 3H), 2.37 – 2.11 (m, 4H), 1.95 – 1.85 (m, 1H), 1.69 – 1.62 (m, 1H), 1.58 – 1.52 (m, 3H), 1.38 – 1.21 (m, 1H), 1.19 (s, 3H), 1.18 (s, 3H).

^{13}C NMR (100 MHz, $CDCl_3$) δ 178.4, 142.4, 114.9, 51.7, 48.3, 44.1, 33.2, 30.3, 27.6, 23.0, 22.7, 14.7.

HRMS (ESI) (*m/z*): $[M+Na]^+$ calcd. for $C_{12}H_{20}O_2Na_1$: 219.135549; found: 219.135410.

Synthesis of compound (–)-18

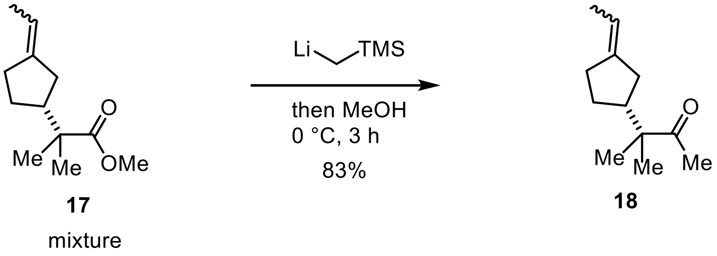

To a flame-dried flask was added (*E/Z*)-methyl 2-(3-ethylidenecyclopentyl)-2-methylpropanoate **17** (4.3 g, 21.9 mmol), and then dry pentane (107 mL) was added under Ar. The solution was cooled to 0 °C. To this mixture was added (trimethylsilyl)methyl lithium (55 mL, 1 M in pentane, 55 mmol, 2.5 equiv.) dropwise, and the temperature was maintained below 10 °C. After **17** was fully consumed (monitored by TLC), methanol (20 mL) was added dropwise to the milky mixture (caution: exothermic reaction). The mixture was stirred for another 2 h and diluted with Et_2O (100 mL) and H_2O (100 mL). The layers were separated, and the aqueous layer was extracted with Et_2O (2 x 80 mL), and all organic phases were combined, dried over anhydrous $MgSO_4$, and filtered. The solvent was removed in *vacuo*, and the residue was purified by chromatography to afford a colorless oil **18** (3.3 g, 83%, *E: Z* = 1: 1.7). *NOTE*: The pure **18**–***Z*** and **18**–*E* isomers were synthesized from an HPLC-separated Wittig product **17–*Z*** and **17–*E***, respectively.

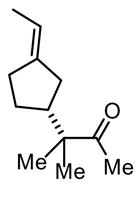

18-*E*

[α]D[25] = 60 (c = 1, $CDCl_3$)

^{1}H NMR (500 MHz, $CDCl_3$) δ 5.27 (ddt, *J* = 6.5, 4.3, 2.9 Hz, 1H), 2.38 – 2.28 (m, 1H), 2.20 – 2.03 (m, 6H), 2.04 – 1.90 (m, 1H), 1.73 – 1.63 (m, 1H), 1.54 (ddq, *J* = 6.8, 3.0, 1.3 Hz, 3H), 1.41 – 1.24 (m, 1H), 1.08 (s, 3H), 1.06 (s, 3H).

^{13}C NMR (125 MHz, $CDCl_3$) δ 213.9, 142.1, 115.1, 49.2, 46.8, 34.8, 28.2, 27.0, 25.6, 21.5, 21.4, 14.5.

HRMS (CI) (*m/z*): $[M]^+$ calcd. for $C_{12}H_{20}O_1$: 180.150865; found: 180.150770.

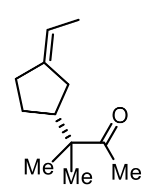

18-*Z*

$[\alpha]_D^{25}$ = 42 (c = 0.7, CH_2Cl_2)

^{1}H NMR (500 MHz, CD_2Cl_2) δ 5.26 (qp, *J* = 6.7, 2.3 Hz, 1H), 2.31 (ddt, *J* = 15.6, 7.6, 1.5 Hz, 1H), 2.26 – 2.16 (m, 3H), 2.11 (s, 3H), 1.83 (ddtd, *J* = 15.4, 13.2, 4.2, 2.0 Hz, 1H), 1.65 – 1.59 (m, 1H), 1.55 (ddt, *J* = 6.8, 2.8, 1.4 Hz, 3H), 1.38 – 1.20 (m, 1H), 1.08 (d, *J* = 11.2 Hz, 6H).

^{13}C NMR (125 MHz, CD_2Cl_2) δ 213.7, 142.7, 115.0, 49.5, 47.1, 34.6, 33.4, 30.2, 27.6, 25.6, 22.8, 21.8, 21.3, 14.8, 14.2.

HRMS (ESI) (*m/z*): $[M+Na]^+$ calcd. for $C_{12}H_{20}O_1Na_1$: 203.140634; found: 203.140560.

Synthesis of compound (–)-19

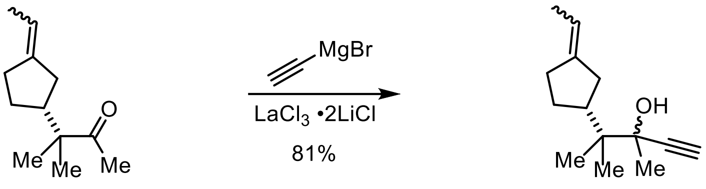

18
mixture

19

To a flame-dried flask was added (*Z/E*)-3-(3-ethylidenecyclopentyl)-3-methylbutan-2-one (3.8 g, 21 mmol) **18** and lanthanum(III) chloride bis(lithium chloride) (37 mL, 0.6 M in THF, 22 mmol, 1.06 equiv.), respectively. The mixture was stirred for 1 h at room temperature and subsequently cooled to 0 °C, and followed by the addition of ethynylmagnesium chloride (70 mL, 0.5 M in THF, 35 mmol, 1.7 equiv.) via syringe at this temperature. After the ketone **18** was fully consumed (monitored by TLC), this mixture was diluted with Et_2O (100 mL), followed by the addition of NH_4Cl (10 mL, sat. aq.), HCl (10 mL, 1M), H_2O (100 mL). The phases were separated, and the aqueous was extracted with Et_2O (2 x 80 mL). All organic phases were combined, dried over anhydrous $MgSO_4$, filtered. The solvent was removed in *vacuo*, and the residue was purified by chromatography to afford a colorless oil **19** (3.5 g, 81%, *E*: *Z* = 1: 1.7). *NOTE*: In order to obtain good analytical data, the **19-*Z*** and **19-*E*** isomers were synthesized from pure **18-*E*** and **18-*Z***, respectively.

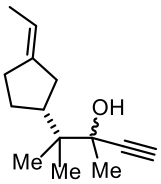

19-*E*

$[\alpha]_D^{25}$ = 61 (c = 1, $CDCl_3$)

^{1}H NMR (500 MHz, $CDCl_3$) δ 5.28 – 5.16 (m, 1H), 2.38 (s, 1H), 2.35 – 2.19 (m, 2H), 2.10 – 1.97 (m, 3H), 1.89 – 1.77 (m, 2H), 1.48 (d, J = 6.5 Hz, 3H), 1.42 (s, 3H), 1.39 – 1.31 (m, 1H), 0.95 (s, 1.5 H), 0.94 (s, 1.5H), 0.92 (s, 1.5 H), 0.91 (s, 1.5 H). (dr 1: 1, mixture).

^{13}C NMR (125 MHz, $CDCl_3$) δ 143.19, 143.17, 114.33, 114.32, 88.39, 88.35, 75.07, 75.01, 72.69, 72.62, 45.97, 45.90, 42.02, 36.71, 36.69, 28.80, 28.77, 28.15, 25.91, 25.85, 20.60, 20.02, 14.48. (dr 1: 1, mixture)

HRMS (ESI) (*m/z*): $[M–H]^-$ calcd. for $C_{14}H_{21}O_1$: 205.159790; found: 205.159760.

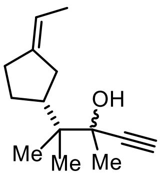

19-*Z*

$[\alpha]_D^{25}$ = 50 (c = 0.7, CH_2Cl_2)

^{1}H NMR (500 MHz, CD_2Cl_2) δ 5.28 – 5.18 (m, 1H), 2.501 (s, 0.38 H), 2.496 (s, 0.46H), 2.45 – 2.33 (m, 1H), 2.31 – 2.24 (m, 1H), 2.22 – 2.11 (m, 2H), 2.0 – 1.98 (m, 2H), 1.86 – 1.80 (m, 1H), 1.61 – 1.51 (m, 3H), 1.48 (s, 3H), 1.40 – 1.31 (m, 1H), 1.02 (s, 1.6H), 1.01 (s, 1.4H), 1.00 (s, 1.8H), 0.99 (s, 1.3H). (dr = 1.2 : 1, mixture). **^{13}C NMR** (125 MHz, CD_2Cl_2) δ 143.18, 143.14, 113.84, 88.31, 88.29, 74.75, 74.71, 72.24, 72.20, 45.92, 45.86, 32.84, 32.82, 31.71, 31.68, 29.08, 29.05, 25.72, 25.67, 22.32, 20.22, 19.52, 14.31.

HRMS (ESI) (m/z): $[M+Na]^+$ calcd. for $C_{14}H_{22}O_1Na_1$: 229.156284; found: 229.156130.

Synthesis of compound (–)-20

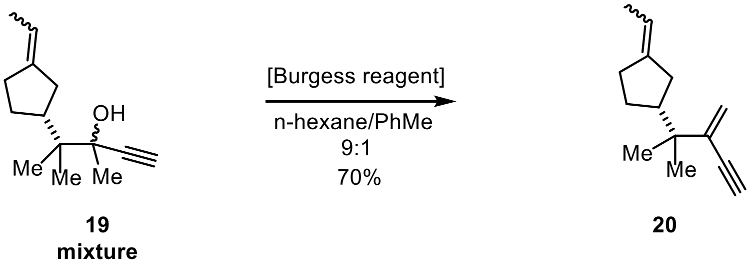

To a flamed-dried flask was added (*Z/E*)-4-(3-ethylidenecyclopentyl)-3, 4-dimethylpent-1-yn-3-ol **19** (1.7 g, 8.2 mmol), followed by addition of dry hexane (46 mL) and dry toluene (5 mL) under Ar. Subsequently, Burgess reagent (4.5 g, 19 mmol, 2.3 equiv.) was added in one portion at room temperature. The reaction mixture was heated to 60 °C and stirred for 19 h. After the alcohol **19** was fully consumed (monitored by TLC and GC), the reaction mixture was cooled to room

temperature, and then was loaded on a pre-cooled silica-gel column to conduct the chromatography to afford a colorless oil **20** (1.1 g, 70%, *E: Z* = 1: 1.7). In order to obtain an optimal analytics, the **20–*Z*** and **20–*E*** isomers were synthesized from **19–*E*** and **19–*Z***, respectively.

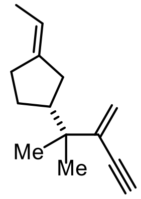

20-*E*

$[\alpha]_D^{25}$ = 59 (c = 1, $CDCl_3$)

^{1}H NMR (500 MHz, CD_2Cl_2) δ 5.49 (d, *J* = 1.1 Hz, 1H), 5.36 (d, *J* = 1.4 Hz, 1H), 5.34 – 5.23 (m, 1H), 2.97 (s, 1H), 2.40 – 2.31 (m, 1H), 2.28 – 2.09 (m, 3H), 2.08 – 1.93 (m, 1H), 1.75 (dddt, *J* = 12.1, 7.9, 5.9, 1.9 Hz, 1H), 1.61 – 1.56 (m, 3H), 1.45 – 1.29 (m, 1H), 1.13 (s, 3H), 1.11 (s, 3H).

^{13}C NMR (125 MHz, CD_2Cl_2) δ 143.5, 140.4, 120.9, 114.5, 84.2, 77.9, 48.4, 40.2, 35.3, 28.8, 27.4, 24.0, 23.7, 14.5.

HRMS (EI) (*m/z*): $[M]^+$ calcd. for $C_{14}H_{20}$: 188.156500; found: 188.156489.

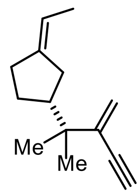

20-*Z*

$[\alpha]_D^{25}$ = 30 (c = 0.7, CH_2Cl_2)

^{1}H NMR (500 MHz, CD_2Cl_2) δ 5.46 (d, *J* = 1.3 Hz, 1H), 5.34 (d, *J* = 1.3 Hz, 1H), 5.27 – 5.19 (m, 1H), 2.94 (s, 1H), 2.33 – 2.12 (m, 4H), 1.92 – 1.75 (m, 1H), 1.65 (dddd, *J* = 13.8, 7.5, 3.5, 1.8 Hz, 1H), 1.55 (ddq, *J* = 6.9, 3.1, 1.5 Hz, 3H), 1.39 – 1.20 (m, 1H), 1.10 (s, 3H), 1.09 (s, 3H).

^{13}C NMR (125 MHz, CD_2Cl_2) δ 143.6, 140.5, 121.0, 114.6, 84.1, 77.9, 48.3, 33.7, 30.5, 27.8, 24.1, 23.5, 22.8, 14.3.

HRMS (EI) (*m/z*): $[M]^+$ calcd. for $C_{14}H_{20}$: 188.155950; found: 188.155950.

Synthesis of compound (–)-21

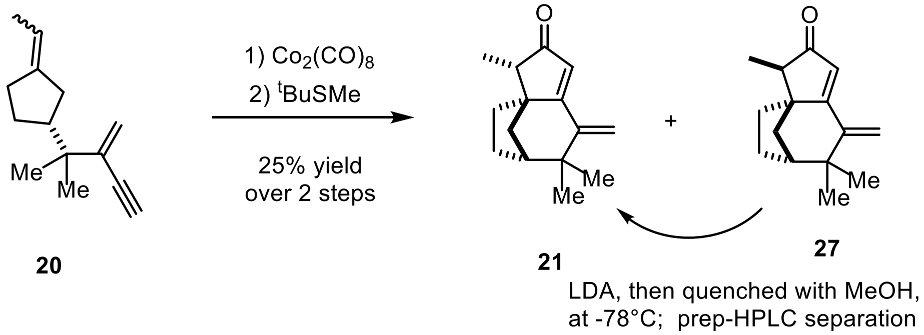

To a flame-dried flask was added (*E/Z*)-1-ethylidene-3-(2-methyl-3-methylenepent-4-yn-2-yl) cyclopentane **20** (0.6 g, 3.3 mmol), diethyl ether (45 mL) and dicobalt octacarbonyl (1.5 g, 90 % moistened with hexane, 3.9 mmol) under Air. (*Caution*: CO gas emission) The resulting black

solution was stirred for 2 h, and after the **20** was fully consumed (monitored by TLC and GC), the solvent was carefully removed *in vacuo*. The residue was purified by column chromatography with pentane to afford a solid black Co-complex, which was used for the next step. To a flamed dried flask was charged the black solid (from the previous step), dry benzene (45 mL) and *tert*-butyl (methyl)sulfane (1.4 mL, 11.4 mmol, 3.5 equiv. based on the **20**). The reaction mixture was heated to 90 °C for 19 h under Air. (***NOTE***: do not seal the reaction flask or preform reaction under Ar, otherwise, it will form an unknown side product). Then the reaction mixture was cooled to room temperature and was loaded on an ice pre-cooled silica-gel column to carry out the chromatography with pentane to wash all benzene and Co-complex. Subsequently, the column was eluted with Et_2O/Pentane (1:4 to 1:3) to afford **21** and **27** as a mixture (180 mg, 25% for two steps, dr 1:1.7, the desired isomer is the minor one). The resulting diastereoisomers were separated by preparative HPLC. (Column: chiralpak IA, 20 µm, 48 mm i.D.; pressure: 100 bar; solvent: *n*-pentane/MTBE 55:45; UV, 254 nm; t_{27} = 15.54 to 22.55 min, t_{21} = 25.53 to 34.17 min; 120 mg/35 min).

The following procedure is used to convert the HPLC-separated undesired **27** to the desired diastereomer **21**. To a flame-dried flask was charged diisopropylamine (0.5 mL, 3.5 mmol, 1.2 equiv.) and dry THF(10 mL) under Ar, and the flask was cooled in an ice-water bath for 10 min. Then *n*-BuLi (1.4 mL, 2.5 M in hexane, 3.5 mmol, 1.2 equiv.) was added to this solution at 5 °C, and the mixture was stirred for 10 min, then it was allowed to warm to room temperature for 30 min. A second dried flask was charged with **27** (635 mg, 2.9 mmol) and dry THF (10 mL) at –78 °C under Ar, followed by the addition of the freshly prepared LDA solution. The mixture was stirred for 10 min and quenched with MeOH (0.5 mL) dropwise. The flask was warmed up to room temperature, diluted with Et_2O (100 mL), and H_2O (80 mL). The organic phase was separated, and the aqueous was extracted with Et_2O (2 x 50 mL). The combined organic phases were dried over anhydrous MgSO4, and the filtrate was concentrated under vacuum to afford a light yellow oil (607 mg, 96%, dr 2: 1 in favor of **21**). The mixture was submitted to the previous prep-HPLC separation.

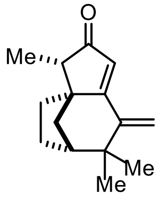

21

[α]$_D^{25}$ = 332 (c = 0.5, CD_2Cl_2)

^{1}H NMR (500 MHz, $CDCl_3$) δ 5.94 (s, 1H), 5.38 (s, 1H), 5.19 (s, 1H), 2.45 (q, *J* = 7.6 Hz, 1H), 1.95 – 1.89 (m, 2H), 1.82 – 1.68 (m, 4H), 1.63 (s, 1H), 1.20 (s, 3H), 1.08 (d, *J* = 7.6 Hz, 3H), 1.07 (s, 3H).

^{13}C NMR (125 MHz, $CDCl_3$) δ 211.4, 182.9, 150.0, 122.2, 114.6, 55.1, 47.6, 47.1, 41.9, 39.8, 29.9, 29.4, 26.1, 25.0, 12.1.

HRMS (ESI) (*m/z*): $[M+Na]^+$ calcd. for $C_{15}H_{20}O_1Na_1$: 239.140634; found: 239.140620.

Er > 99:1

The enantiomeric purity was measured by chiral GC (Column: 25 m, HYDRODEX BTBDAC G 681; i.D. 0.25 mm; Temperature: 150 °C, 35 min, 8 °C/min gradient increment to 220 °C, 3 min; gas: 0.5 bar H_2), $t_{\mathbf{21}minor}$ = 28.91 min, $t_{\mathbf{21}major}$ = 29.83 min.

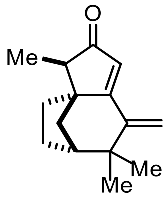

27

[α]$_D^{25}$ = 429 (c = 0.6, $CDCl_3$)

^{1}H NMR (500 MHz, $CDCl_3$) δ 5.88 (s, 1H), 5.32 (s, 1H), 5.16 (s, 1H), 2.33 (q, *J* = 7.6 Hz, 1H), 1.96 (t, *J* = 5.6 Hz, 1H), 1.88 – 1.82 (m, 1H), 1.78 – 1.67 (m, 3H), 1.63 (dd, *J* = 11.7, 4.8 Hz, 1H), 1.59 – 1.52 (m, 1H), 1.20 (s, 3H), 1.12 (d, *J* = 7.6 Hz, 3H), 1.02 (s, 3H).

^{13}C NMR (125 MHz, $CDCl_3$) δ 211.6, 183.4, 150.4, 122.4, 114.0, 55.6, 47.7, 47.4, 42.0, 35.8, 34.5, 29.4, 25.9, 24.9, 11.6.

Er > 99:1

The enantiomeric purity was measured by chiral GC (Column: 25 m, HYDRODEX BTBDAC G 681; i.D. 0.25 mm; Temperature: 150 °C, 35 min, 8 °C/min gradient increment to 220 °C, 3 min; gas: 0.5 bar H_2), $t_{\mathbf{27}minor}$ = 26.35 min, $t_{\mathbf{27}major}$ = 27.01 min.

HRMS (ESI) (*m/z*): $[M+Na]^+$ calcd. for $C_{15}H_{20}O_1Na_1$: 239.140634; found: 239.140440.

Synthesis of compound (–)-22

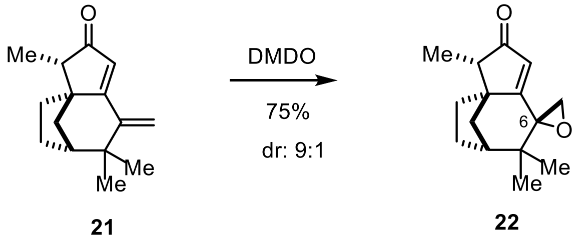

Fresh dimethyldioxirane was prepared from Oxone (25 g), $NaHCO_3$ (24 g) in H_2O (20 mL), and acetone (30 mL) followed by the procedure of Douglass F. Taber et al.[3] To a solution of **21** (415 mg, 1.9 mmol) in dry DCM (20 mL) at 0 °C was added slowly fresh dimethyldioxirane solution in acetone (20 mL). After **21** was fully consumed (monitored by TLC), the reaction mixture was bubbled with Ar for 10 min and concentrated carefully in *vacuo.* The residue was purified by column chromatography to afford white oil **22**, which could be solidified in the freeze (330 mg, 75%, dr 9:1 at C6 determined by GC).

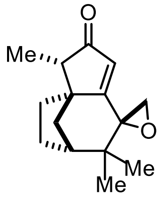

22

[α]$_D$^{25} = 74 (c = 1, Et_2O)

^{1}H NMR (500 MHz, $CDCl_3$) δ 5.76 (s, 1H), 2.93 (d, *J* = 4.1 Hz, 1H), 2.81 (d, *J* = 4.1 Hz, 1H), 2.40 (q, *J* = 7.6 Hz, 1H), 2.18 – 2.13 (m, 1H), 1.98 – 1.96 (m, 1H), 1.93 – 1.75 (m, 5H), 1.11 (d, *J* = 7.6 Hz, 3H), 1.03 (d, *J* = 2.4 Hz, 3H), 0.88 (s, 3H).

^{13}C NMR (125 MHz, CD_2Cl_2) δ 211.3, 181.9, 124.9, 59.0, 56.1, 47.9, 47.2, 45.2, 41.1, 38.4, 28.8, 25.8, 24.8, 20.7, 12.8.

HRMS (ESI) (*m/z*): [M+Na]$^+$ calcd. for C_{15} H_{20} O_2 Na_1: 255.135549; found: 255.135350.

Synthesis of compound (–)-23

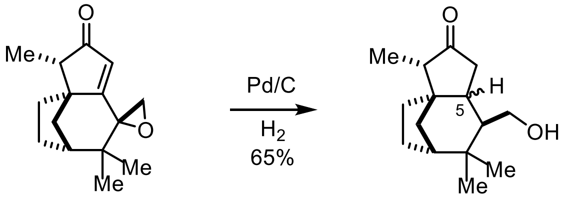

To a solution of **22** (100 mg, 0.4 mmol) in dry ethyl acetate (35 mL) at room temperature was added palladium on activated charcoal (1.3 g, 10% Pd, 3 equiv.). The flask was sealed with a rubber septum and was placed under vacuum via a needle, then back refilled with hydrogen gas. The procedure was repeated three times. After 30 min, the epoxide was fully consumed (monitored by TLC), and the reaction mixture was through a short pad of celite, the filtrate was concentrated under reduced pressure, and the sample was submitted to a GC analysis. Subsequently, the residue was purified by column chromatography on silica gel to afford an inseparable diastereomeric mixture **23** (66 mg, 65%, dr 2:1 at C5 determined by NMR). (*Note*: the Pd on carbon from the different companies could primarily affect the yield and diastereoselectivity of hydrogenation reaction. In this paper, the Pd on active carbon type 487 from *Alfa Aesar* was used.)

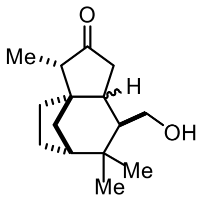

23

$[\alpha]_D^{25}$ = 73 (c = 0.47, $CDCl_3$)

^{1}H NMR (500 MHz, $CDCl_3$) δ 3.72 (ddd, *J* = 10.8, 4.8, 2.7 Hz, 1H), 3.67 (dd, *J* = 10.8, 5.6 Hz, 0.5 H), 3.54 – 3.45 (m, 1.5H), 2.45 (dd, *J* = 18.1, 9.2 Hz, 0.5 H), 2.36 (dddd, *J* = 13.8, 12.5, 10.6, 1.4 Hz, 1H), 2.29 – 2.14 (m, 2.5H), 2.07 – 1.96 (m, 0.5H), 1.93 – 1.82 (m, 2H), 1.75 – 1.55 (m, 6.5 H), 1.46 (dd, *J* = 11.4, 4.8 Hz, 1H), 1.33 – 1.24 (m, 3H), 1.23 – 1.17 (m, 2H), 1.02 (s, 1.5H), 0.97 – 0.94 (m, 4H), 0.89 – 0.88 (m, 4H), 0.86 (s, 2H), 0.84 (s, 1.5H).

^{13}C NMR (125 MHz, CD_2Cl_2) δ 219.4, 63.8, 51.2, 49.9, 47.9, 44.8, 41.0, 38.2, 36.9, 33.2, 27.7, 25.8, 22.8, 22.4, 7.7 (major isomer).

^{13}C NMR (125 MHz, CD_2Cl_2) δ 219.8, 64.4, 51.4, 49.3, 48.7, 46.9, 44.6, 42.3, 37.2, 36.8, 35.9, 33.8, 24.4, 23.1, 11.5(minor isomer).

HRMS (CI) (*m/z*): $[M+H]^+$ calcd. for $C_{15}H_{20}O_2$: 237.184905; found: 237.184910.

Synthesis of compound (–)-10

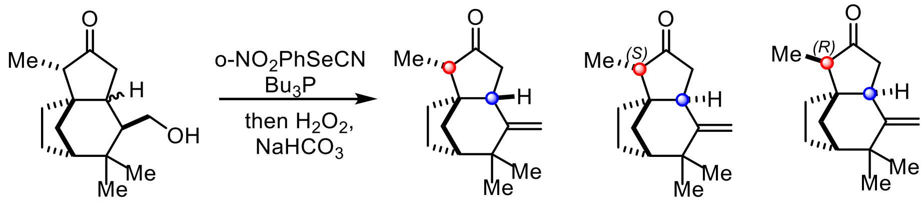

23 10 24 25

To a solution of **23** (170 mg, 0.72 mmol) in dry THF (6 mL) was added 1-nitro-2-selenocyanatobenzene (209 mg, 1.28 equiv.) under Ar. At room temperature, tributylphosphine (404 mg, 90% purity, 2.5 equiv.) was added dropwise over 3 min via a syringe. The mixture was stirred for 1 h. Then solid $NaHCO_3$ (1.8 g, 22 mmol, 30 equiv.) was added, followed by dropwise addition of H_2O_2 (30% aq., 0.7 mL, 30 equiv.) over 5 min at room temp. After stirring for 4 h at room temperature, the reaction mixture was loaded directly on a pre-cooled column with silica gel to carry out the chromatography (elute: pentane, then Et_2O: pentane 1:100 to 1:20) to afford three diastereoisomers (102 mg, 65%, GC purity: **10,** 77.22%; **24**, 25.29%; **25**, 2.48%). These three isomers were subjected to a prep-HPLC for separation. (Column: Chiralpak IA, 250 mm Chiral Tech, 20 mm i.D.; pressure: 6.1 Mpa; solvent: *n*-pentane/MTBE 97:3; UV, 204 nm; 20 mL/1 min; t_{10} = 23.5 to 27.2 min, t_{25} = 30.5 to 33.8 min, t_{24} = 33.8 to 40.2 min).

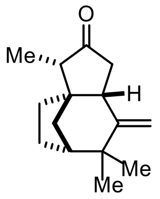

10

[α]$_D^{25}$ = 132 (c = 0.33, $CDCl_3$)

^{1}H NMR (500 MHz, $CDCl_3$) δ 4.85 (dd, *J* = 2.0, 0.9 Hz, 1H), 4.57 (d, *J* = 2.1 Hz, 1H), 2.79 (ddq, *J* = 11.2, 7.3, 2.0 Hz, 1H), 2.41 – 2.35 (m, 1H), 2.29 (ddd, *J* = 18.5, 7.4, 1.3 Hz, 1H), 2.13 (dd, *J* = 18.5, 12.1 Hz, 1H), 1.89 (dt, *J* = 11.4, 2.2 Hz, 1H), 1.84 (t, *J* = 5.3 Hz, 1H), 1.64 (dd, *J* = 11.4, 4.7 Hz, 1H), 1.61 – 1.44 (m, 3H), 1.17 (s, 3H), 1.11 (s, 3H), 0.96 (d, *J* = 7.0 Hz, 3H).

^{13}C NMR (125 MHz, $CDCl_3$) δ 218.8, 154.6, 106.4, 52.4, 51.9, 48.3, 46.9, 40.9, 38.9, 38.2, 28.5, 26.4, 26.1, 21.6, 8.1.

HRMS (ESI) (*m/z*): [M+Na]$^+$ calcd. for $C_{15}H_{22}ONa_1$: 241.156284; found: 241.156020.

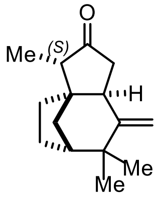

24

[α]$_D^{25}$ = –64 (c = 0.65, $CDCl_3$)

^{1}H NMR (500 MHz, $CDCl_3$) δ 5.00 (d, *J* = 1.5 Hz, 1H), 4.90 (dt, *J* = 1.5, 0.8 Hz, 1H), 2.80 (ddt, *J* = 11.7, 9.8, 2.0, 0.8 Hz, 1H), 2.46 (ddd, *J* = 19.0, 11.7, 0.8 Hz, 1H), 2.41 (dd, *J* = 19.0, 9.8 Hz, 1H), 2.08 (qd, *J* = 7.8, 0.8 Hz, 1H), 2.00 (dt, *J* = 12.3, 2.1 Hz, 1H), 1.79 (ddd, *J* = 7.0, 5.3, 0.8 Hz, 2H), 1.69 (m, 1H), 1.40 (m, 2H), 1.18 (ddd, *J* = 12.3, 5.3, 2.0 Hz, 1H), 1.16 (s, 3H), 1.1(s, 3H), 1.04 (d, *J* = 7.8 Hz, 3H).

^{13}C NMR (125 MHz, $CDCl_3$) δ 221.6, 152.6, 113.4, 51.8, 51.3, 48.5, 46.9, 40.4, 36.6, 31.4, 30.8, 28.1, 24.3, 14.1, 12.8.

HRMS (CI) (*m/z*): [M]$^+$ calcd. for $C_{15}H_{22}O$: 218.166515; found: 218.166400.

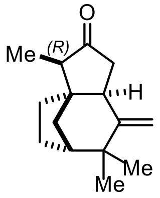

25

[α]$_D^{25}$ = –25 (c = 0.15, CH_2Cl_2)

^{1}H NMR (500 MHz, $CDCl_3$) δ 5.01 (d, *J* = 1.4 Hz, 1H), 4.91 (dt, *J* = 1.5, 0.8 Hz, 1H), 2.59 (dddd, *J* = 12.6, 8.9, 2.0, 0.8 Hz, 1H), 2.47 (ddd, *J* = 19.0, 8.9, 1.2 Hz, 1H), 2.28 (dd, *J* = 19.0, 12.6 Hz, 1H), 2.19 (qd, *J* = 7.0, 1.2 Hz, 1H), 1.81 (m, 1H), 1.71 – 1.63 (m, 3H), 1.57 (dd, *J* = 12.2, 2.3 Hz, 1H), 1.36 (m, 1H), 1.21 (ddd, *J* = 12.2, 5.4, 2.0 Hz, 1H), 1.12 (s, 6H), 0.97 (d, *J* = 7.0 Hz, 3H).

^{13}C NMR (125 MHz, $CDCl_3$) δ 219.3, 152.9, 113.2, 51.7, 51.1, 51.0, 46.7, 44.6, 40.6, 32.5, 31.5, 29.7, 28.1, 24.6, 7.3.

HRMS (ESI) (*m/z*): [M+Na]$^+$ calcd. for $C_{15}H_{22}O_1$: 218.166515; found: 218.166690.

Elucidation of the absolute configuration of the Mukaiyama–Michael addition product

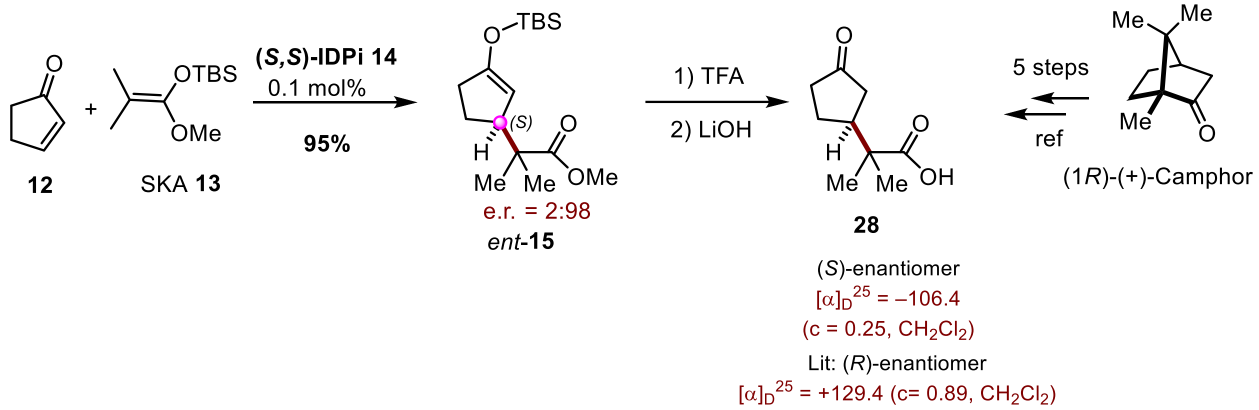

Fig. S4. The elucidation of the absolute configuration of Mukaiyama–Michael adduct

The absolute configuration of the stereogenic center generated during the Mukaiyama–Michael addition was elucidated by comparison of the optical value of **28** with the literature report, in which the *R*- carboxylic acid was derived from (1*R*)-(+)-Camphor.[4] Employing the (*S*, *S*)-IDPi **14**, the *ent*-**15** was obtained with an enantiomeric ratio of 2:98 in the opposite configuration. Correspondingly, the catalyst (*R*, *R*)-IDPi **14** was engaged for this transformation.

Hydrolysis procedure: to a flask was added the desilicated ketone ester *ent*-**16** (280 mg, 1.5 mmol, 96% ee), THF (5 mL), and MeOH (5 mL). To this mixture was added LiOH (363 mg, 15 mmol, 10 equiv.) and H_2O (0.4 mL). The resulting mixture was heated to 70 °C until the starting material was fully consumed (monitored by TLC). Et_2O (50 mL) and H_2O (50 mL) were added to this mixture, and then the organic layer was separated, dried over anhydrous $MgSO_4$. After the filtration, the filtrate was concentrated in *vacuo*. The residue was purified by column chromatography to afford enough materials **28** to determine the absolute configuration.

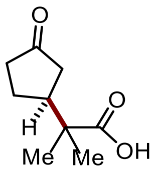

28

$[\alpha]_D^{25}$ = –106.4 (c = 0.25, CH_2Cl_2)

^{1}H NMR (500 MHz, $CDCl_3$) δ 11.01 (s, 1H), 2.47 (tdd, *J* = 11.9, 7.6, 6.1 Hz, 1H), 2.41–2.27 (m, 2H), 2.24–2.13 (m, 1H), 2.12–2.03 (m, 2H), 1.68 (qd, J = 11.9, 8.4 Hz, 1H), 1.23 (s, 3H), 1.22 (s, 3H).

^{13}C NMR (125 MHz, $CDCl_3$) δ) δ 218.52, 183.37, 44.89, 43.81, 40.52, 39.01, 24.56, 22.66, 22.54.

HRMS (*m/z*): $[M – H]^–$ calcd. for $C_9H_{13}O_3$: 169.0870; found: 169.0870.

Attempts towards selective 1, 4-reduction of dienone 21

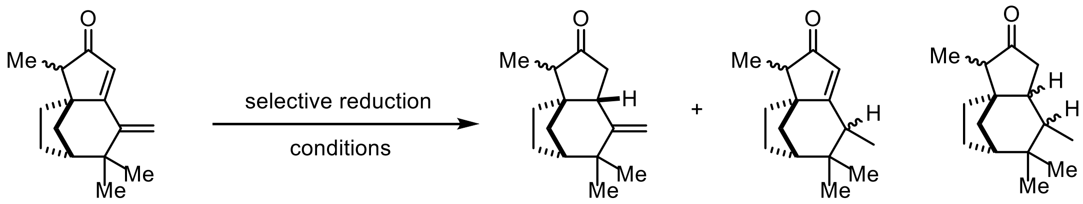

21+27 mixture **10** **29** **30**

Entry	condition	result
1	Stryker's reagent, toluene, rt	full conv., **29:** 48%, **10:** ND
2	$PhSiH_3$, $Pd(PPh_3)_4$, $ZnCl_2$, $CHCl_3$, H_2O, open flask, rt	no full conv., **29:** 18%, **10:** ND
3	Et_3SiH, $(PPh_3)_3RhCl$, toluene, K_2CO_3, MeOH, rt	full conv., **29:** 5%, **10:** ND
4	Hantzsch ester, *S*-TRIP, Bu_2O, 60 °C	no reaction
5	Zn, KOH, MeOH, H_2O, reflux	recovered 27% sm, **10**: ND
6	Et_3SiH, $TiCl_4$, CH_2Cl_2, 0 °C	full conv.,**29:** 23%, **10**: ND
7	$[IrCp^*Cl_2]_2$, KOAc, *i*PrOH	recovered 81% sm, **10**: ND
8	L-Selectride, THF, 0 °C	decomposed
9	CuI, $LiAlH_4$, THF, 0 °C to rt	full conv.,**29:** 15%, **10**: ND
10[a]	$B(C_6F_5)_3$, Et_3SiH, HFIP, 40 °C	**21:29**=1:1.6, **10**: ND
11	SmI_2, LiCl, HMPA, THF, *t*BuOH	full conv.,**29:** ND, **10**: ND, **30:** >50%

A mixture of **21** and **27** was used for screenning unless otherwise indicated. a) enantiopure **21** was used. Conv. = conversion, ND = not determined. THF, tetrahydrofuran, HFIP, hexafluoro-2-propanol, HMPA, Hexamethylphosphoramide.

Table S1. Selected conditions for 1,4-reduction of dienone **21**

Although a variety of selective 1, 4-reduction conditions were tested, none of them could deliver the desired 1, 4-reducted product. The corresponding 1, 6-reduction product from the dienone **21** was obtained dominantly, suggesting that the exocyclic double react preferentially. Therefore, an indirect way was performed.

References

[1] P. S. Kaib, L. Schreyer, S. Lee, R. Properzi, B. List, *Angew. Chem. Int. Ed.* **2016**, *55*, 13200–13203.
[2] T. Gatzenmeier, P. S. J. Kaib, J. B. Lingnau, R. Goddard, B. List, *Angew. Chem. Int. Ed.* **2018**, *57*, 2464–2468.
[3] F. T. Douglass, W. D. Peter, A. Rasha, *Org. Synth.* **2013**, *90*, 350–357.
[4] G. M. A., E. Teso Vilar, A. García Fraile, S. de la Moya Cerero, Martínez, P. Ruiz, L. R. Subramanian, *Tetrahedron: Asymmetry* **1996**, *7*, 2177–2180.

Single-Crystal X-ray Diffraction Analysis of epoxide 22 (CCDC # 2041474)

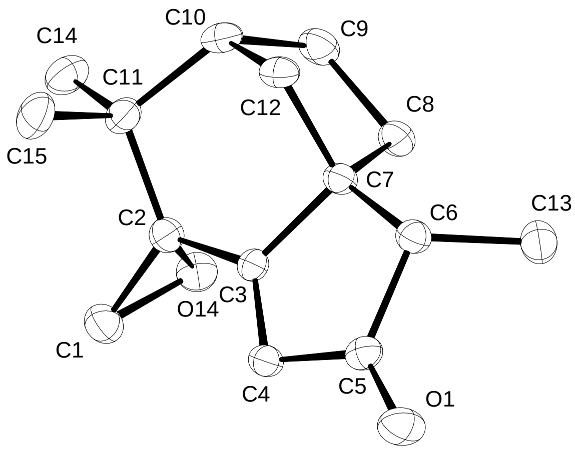

Fig S5. The molecular structure of **22**

Table S2. Crystal data and structure refinement.

Identification code	12776	
Empirical formula	$C_{15} H_{20} O_2$	
Color	colourless	
Formula weight	232.31 g · mol^{-1}	
Temperature	100(2) K	
Wavelength	1.54178 Å	
Crystal system	ORTHORHOMBIC	
Space group	**$P2_12_12_1$, (no. 19)**	
Unit cell dimensions	a = 7.9073(2) Å	α= 90°.

	b = 11.5422(3) Å	β= 90°.
	c = 13.6489(4) Å	γ = 90°.
Volume	1245.70(6) $Å^3$	
Z	4	
Density (calculated)	1.239 Mg · m^{-3}	
Absorption coefficient	0.632 mm^{-1}	
F(000)	504 e	
Crystal size	0.200 x 0.171 x 0.161 mm^3	
θ range for data collection	5.018 to 72.295°.	
Index ranges	-9 ≤ h ≤ 9, -14≤ k ≤ 14, -16 ≤ l ≤ 16	
Reflections collected	45419	
Independent reflections	2442 [R_{int} = 0.0382]	
Reflections with I>2σ(I)	2321	
Completeness to θ = 67.679°	100.0 %	
Absorption correction	Gaussian	
Max. and min. transmission	0.94 and 0.92	
Refinement method	Full-matrix least-squares on F^2	
Data / restraints / parameters	2442 / 0 / 157	
Goodness-of-fit on F^2	1.125	
Final R indices [I>2σ(I)]	R_1 = 0.0309	wR^2 = 0.0721
R indices (all data)	R_1 = 0.0358	wR^2 = 0.0752
Absolute structure parameter	0.05(4)	
Largest diff. peak and hole	0.2 and -0.2 e · $Å^{-3}$	

Table S3. Bond lengths [Å] and angles [°].

O(1)-C(5)	1.220(2)	O(14)-C(1)	1.447(2)
O(14)-C(2)	1.460(2)	C(1)-C(2)	1.464(3)
C(2)-C(3)	1.496(2)	C(2)-C(11)	1.537(2)
C(3)-C(4)	1.337(2)	C(3)-C(7)	1.513(2)
C(4)-C(5)	1.466(3)	C(5)-C(6)	1.531(2)
C(6)-C(7)	1.546(2)	C(6)-C(13)	1.523(2)
C(7)-C(8)	1.548(2)	C(7)-C(12)	1.541(2)
C(8)-C(9)	1.549(3)	C(9)-C(10)	1.544(3)
C(10)-C(11)	1.551(3)	C(10)-C(12)	1.534(3)
C(11)-C(14)	1.532(2)	C(11)-C(15)	1.540(2)

C(1)-O(14)-C(2)	60.47(11)	O(14)-C(1)-C(2)	60.22(11)
O(14)-C(2)-C(1)	59.32(11)	O(14)-C(2)-C(3)	112.32(13)
O(14)-C(2)-C(11)	116.60(14)	C(1)-C(2)-C(3)	119.30(15)
C(1)-C(2)-C(11)	123.17(16)	C(3)-C(2)-C(11)	113.67(14)
C(2)-C(3)-C(7)	117.73(14)	C(4)-C(3)-C(2)	128.49(16)
C(4)-C(3)-C(7)	113.77(15)	C(3)-C(4)-C(5)	109.41(15)
O(1)-C(5)-C(4)	126.71(16)	O(1)-C(5)-C(6)	124.77(17)
C(4)-C(5)-C(6)	108.52(14)	C(5)-C(6)-C(7)	104.56(14)
C(13)-C(6)-C(5)	110.88(14)	C(13)-C(6)-C(7)	117.97(15)
C(3)-C(7)-C(6)	103.66(13)	C(3)-C(7)-C(8)	109.33(14)
C(3)-C(7)-C(12)	107.89(14)	C(6)-C(7)-C(8)	118.21(14)
C(12)-C(7)-C(6)	116.43(15)	C(12)-C(7)-C(8)	101.02(14)
C(7)-C(8)-C(9)	105.28(14)	C(10)-C(9)-C(8)	105.71(14)
C(9)-C(10)-C(11)	112.54(15)	C(12)-C(10)-C(9)	101.13(15)
C(12)-C(10)-C(11)	112.15(14)	C(2)-C(11)-C(10)	109.12(14)
C(2)-C(11)-C(15)	107.09(14)	C(14)-C(11)-C(2)	112.05(15)
C(14)-C(11)-C(10)	109.77(14)	C(14)-C(11)-C(15)	108.28(15)
C(15)-C(11)-C(10)	110.50(15)	C(10)-C(12)-C(7)	101.14(14)

NMR Spectra

^{1}H NMR (500 MHz, CD_2Cl_2) and ^{13}C NMR (125 MHz, CD_2Cl_2) of diol

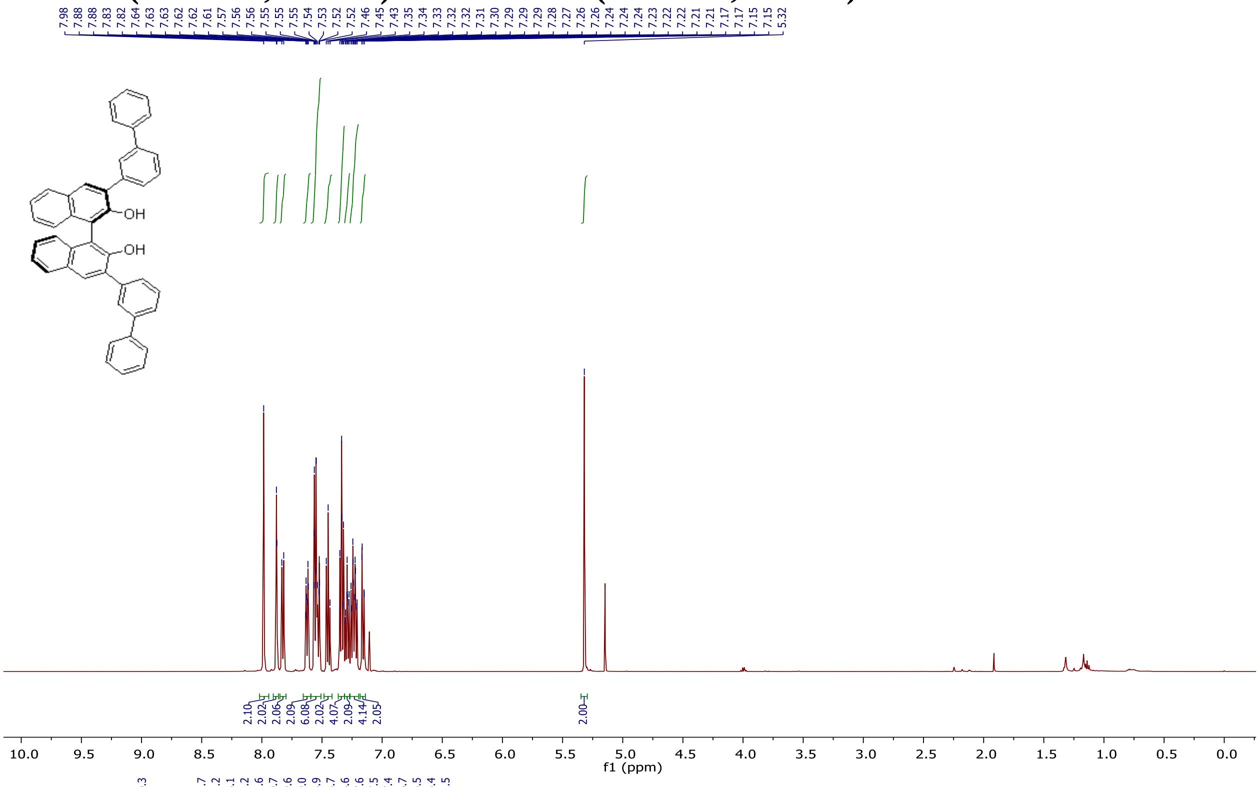

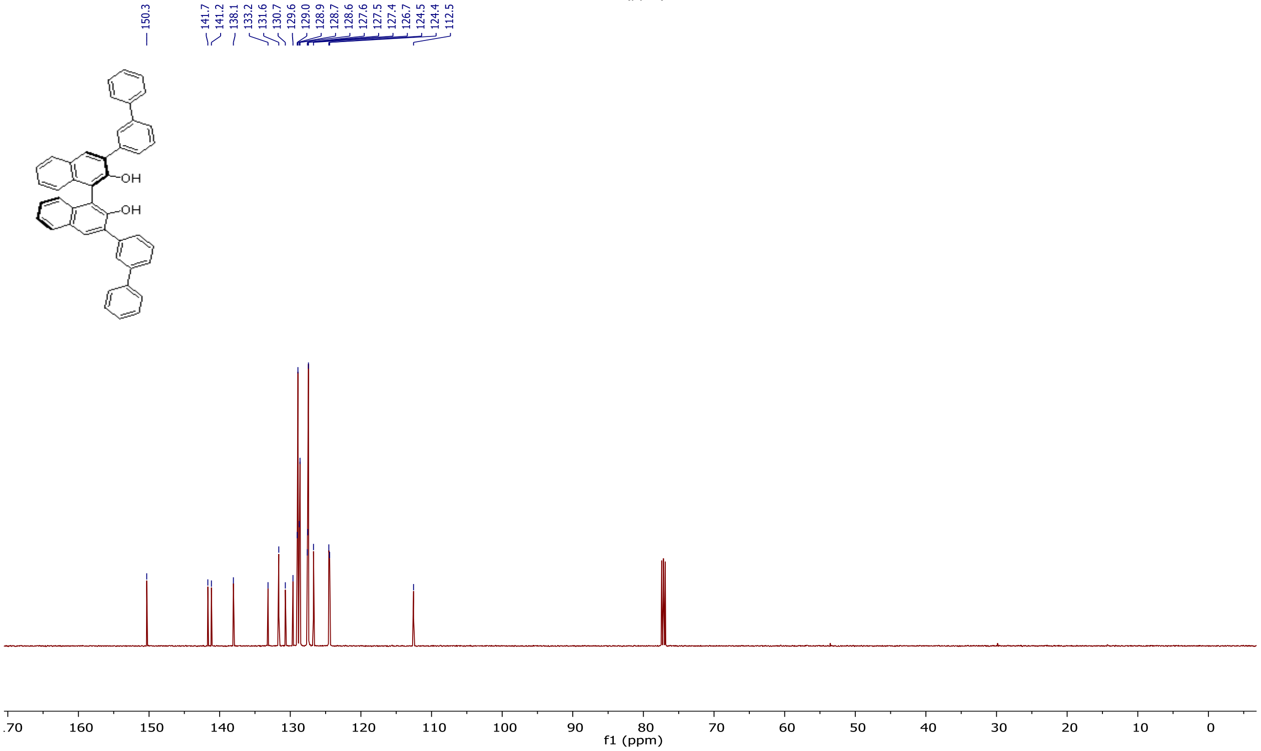

^{31}P NMR (203 MHz, CD_2Cl_2), ^{1}H NMR (500 MHz, CD_2Cl_2) of IDPi-14

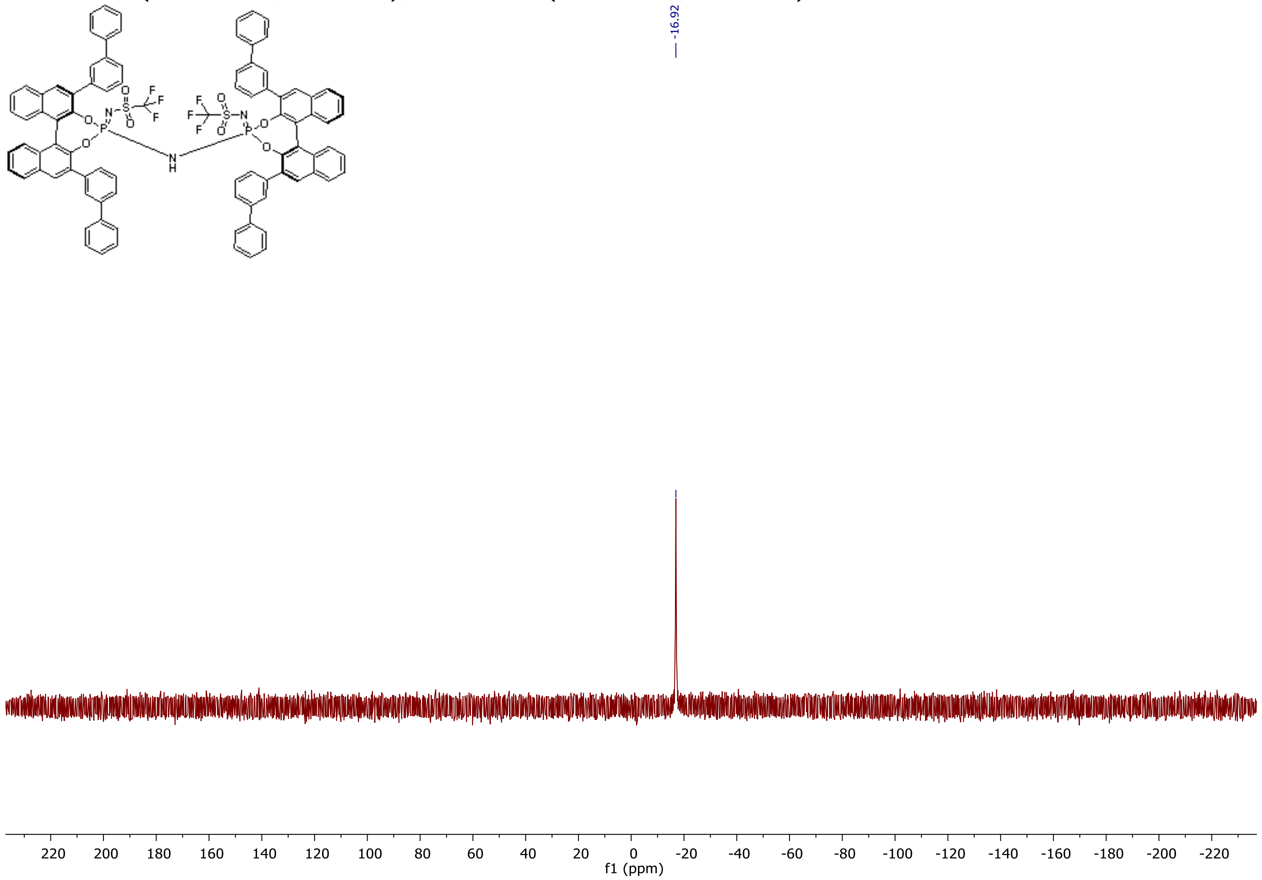

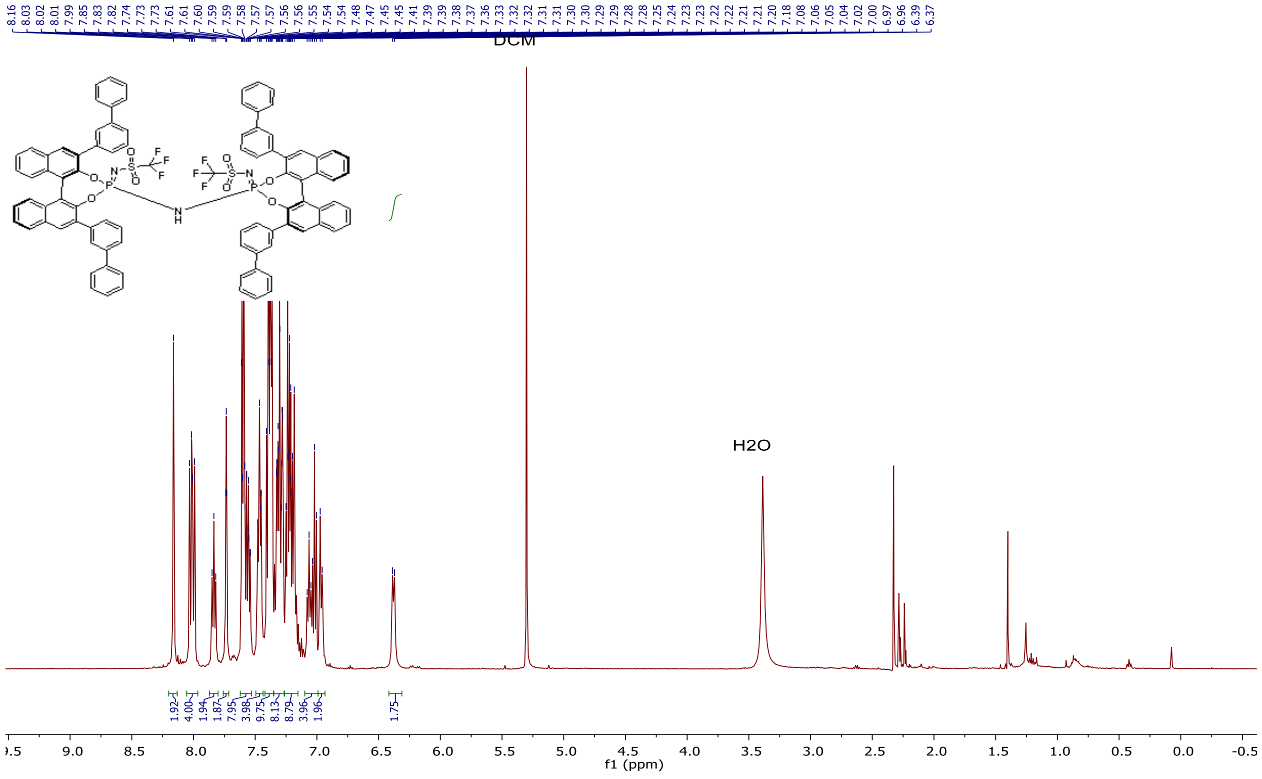

^{13}C NMR (125 MHz, CD_2Cl_2), ^{19}F NMR (470 MHz, CD_2Cl_2) of IDPi-14

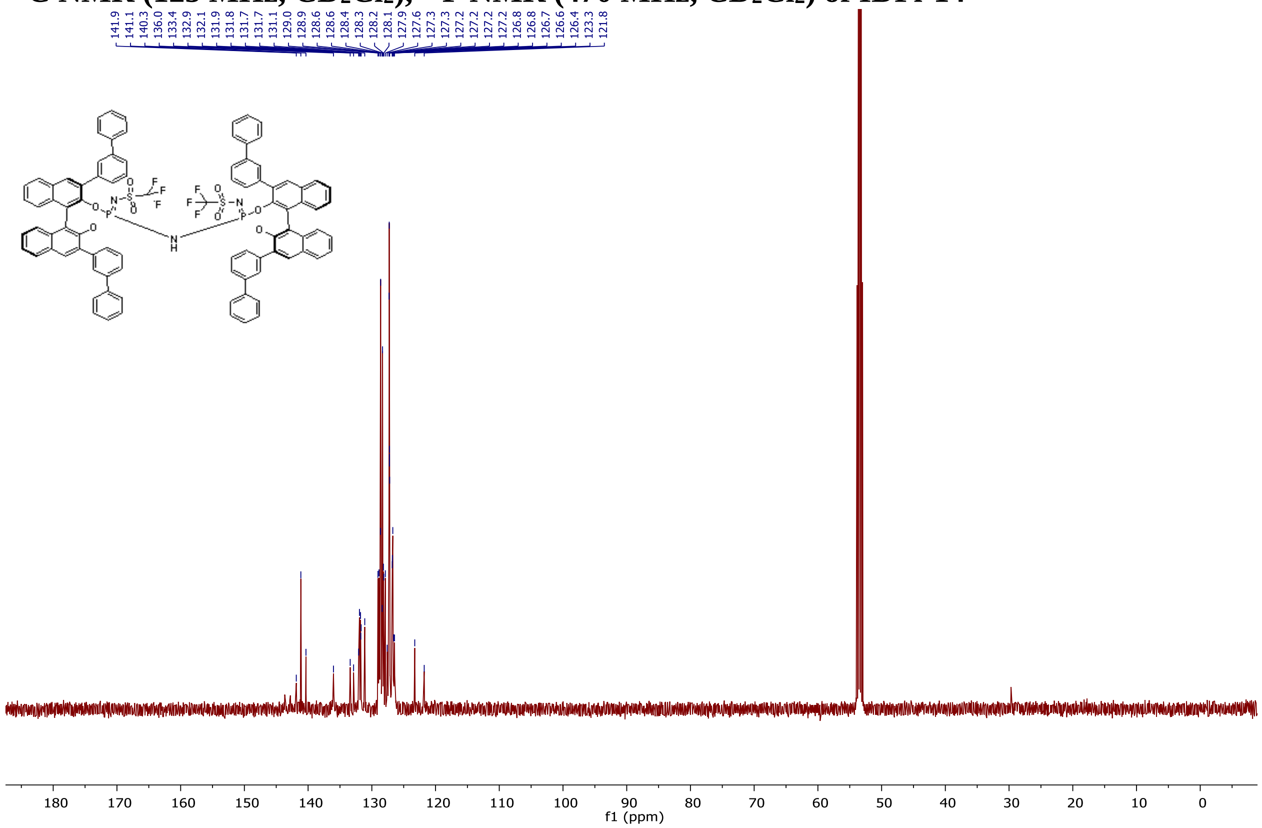

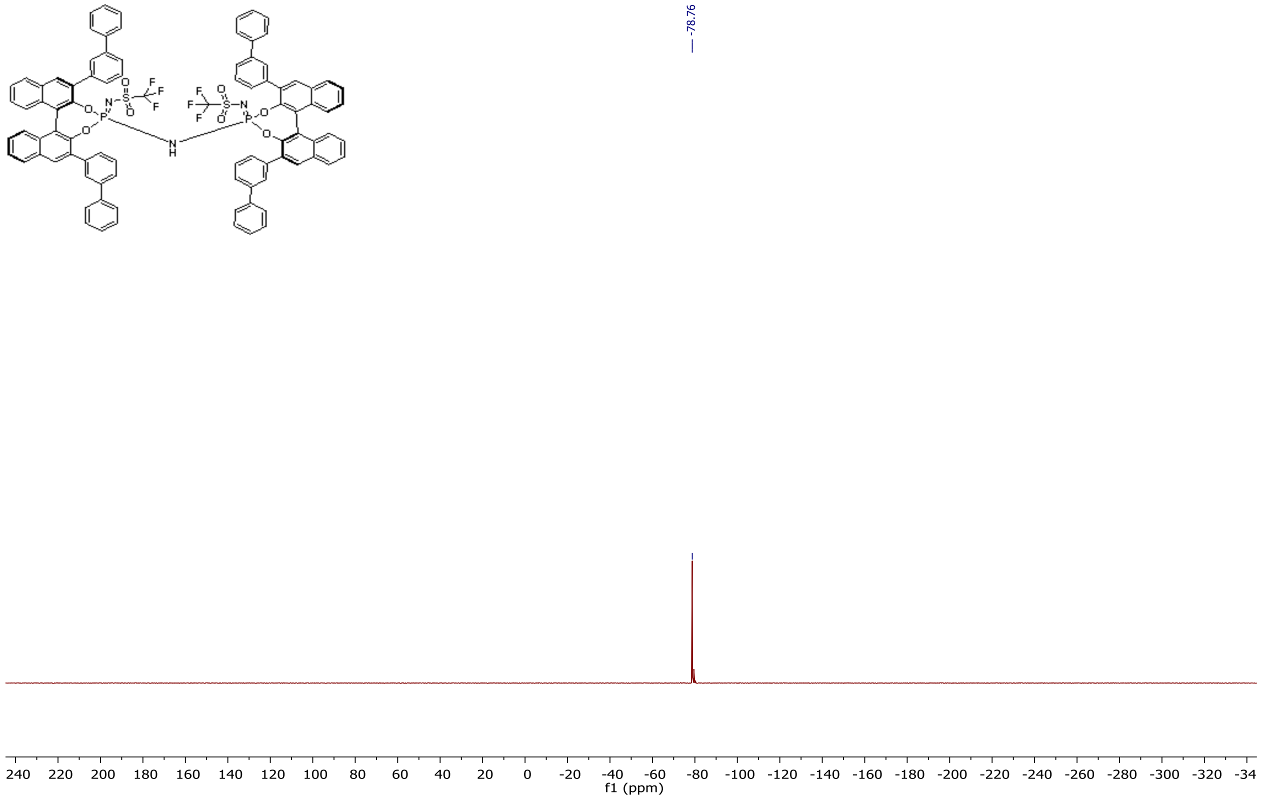

¹H NMR (500 MHz, $CDCl_3$) and ¹³C NMR (125 MHz, $CDCl_3$) of 15

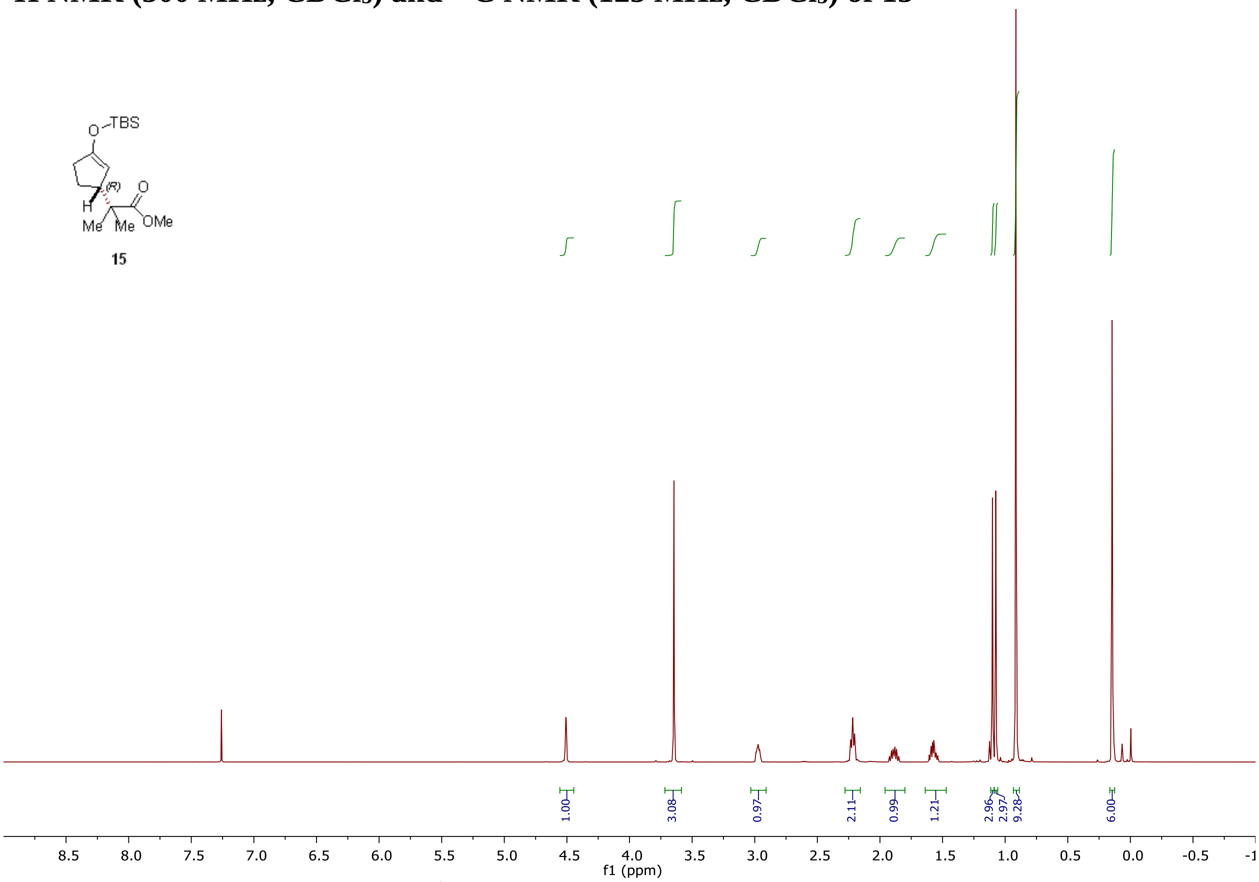

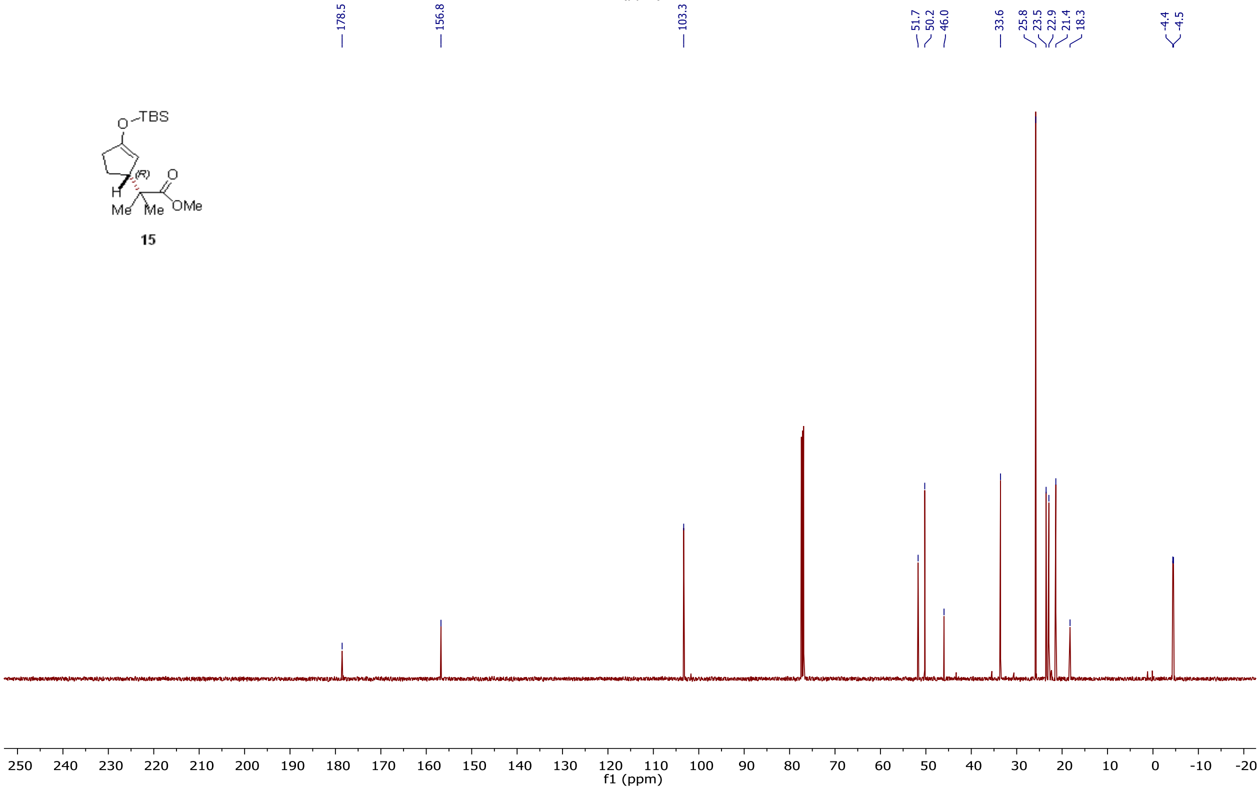

^{1}H NMR (500 MHz, $CDCl_3$) and ^{13}C NMR (125 MHz, $CDCl_3$) of 16

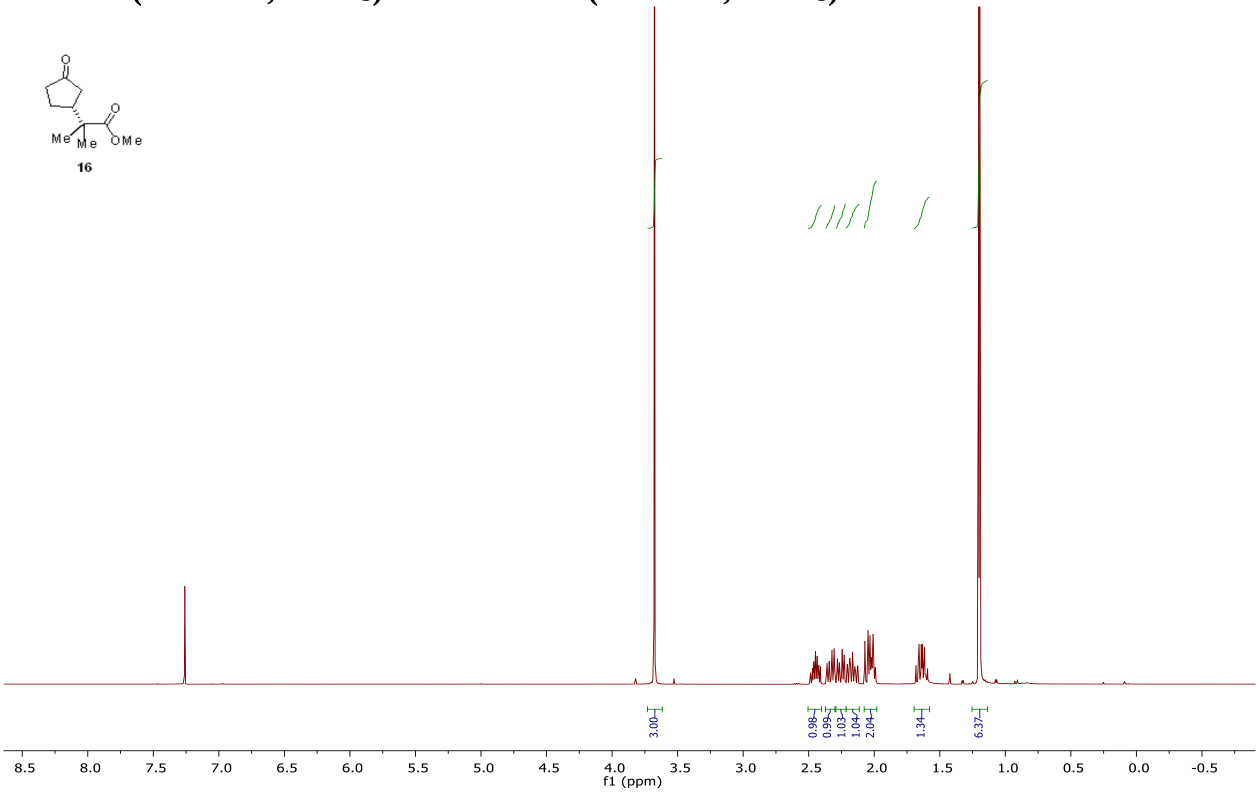

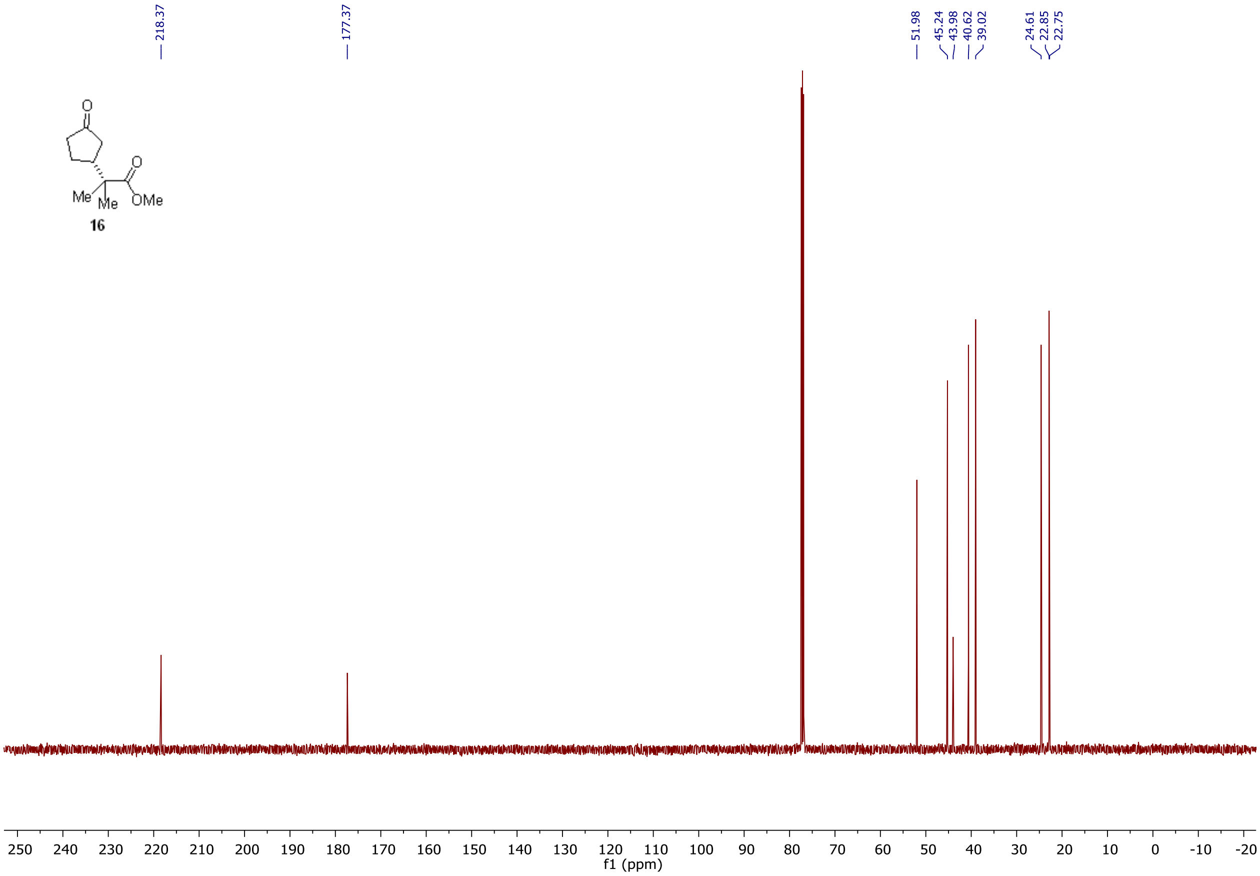

^{1}H NMR (500 MHz, $CDCl_3$) and ^{13}C NMR (125 MHz, $CDCl_3$) of 17-*E*

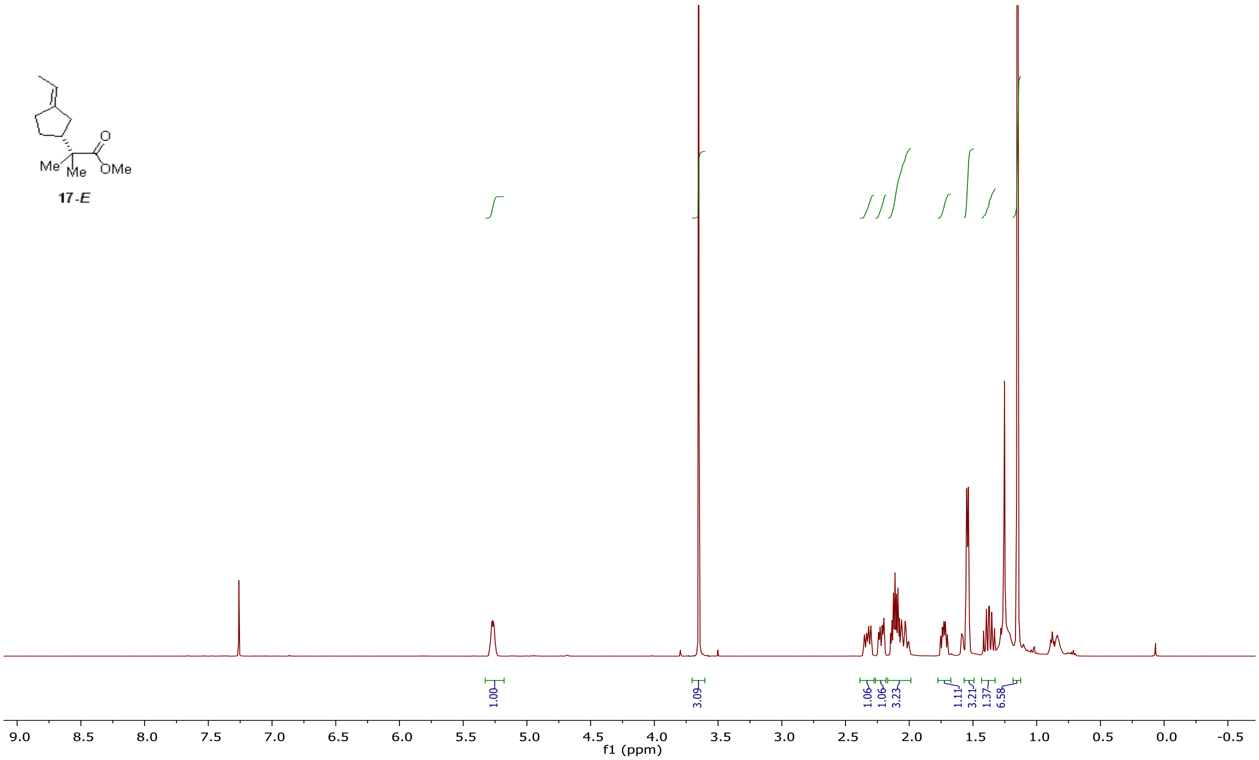

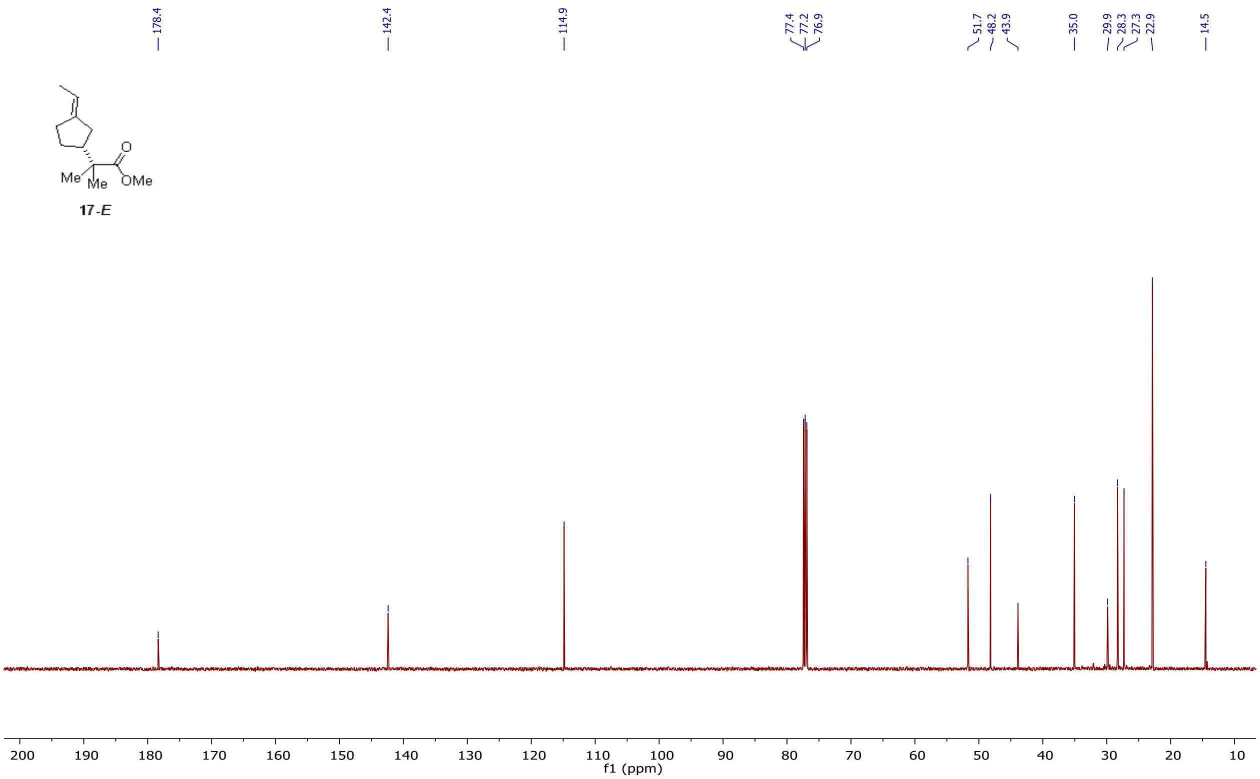

^{1}H NMR (500 MHz, $CDCl_3$) and ^{13}C NMR (125 MHz, $CDCl_3$) of 17-*Z*

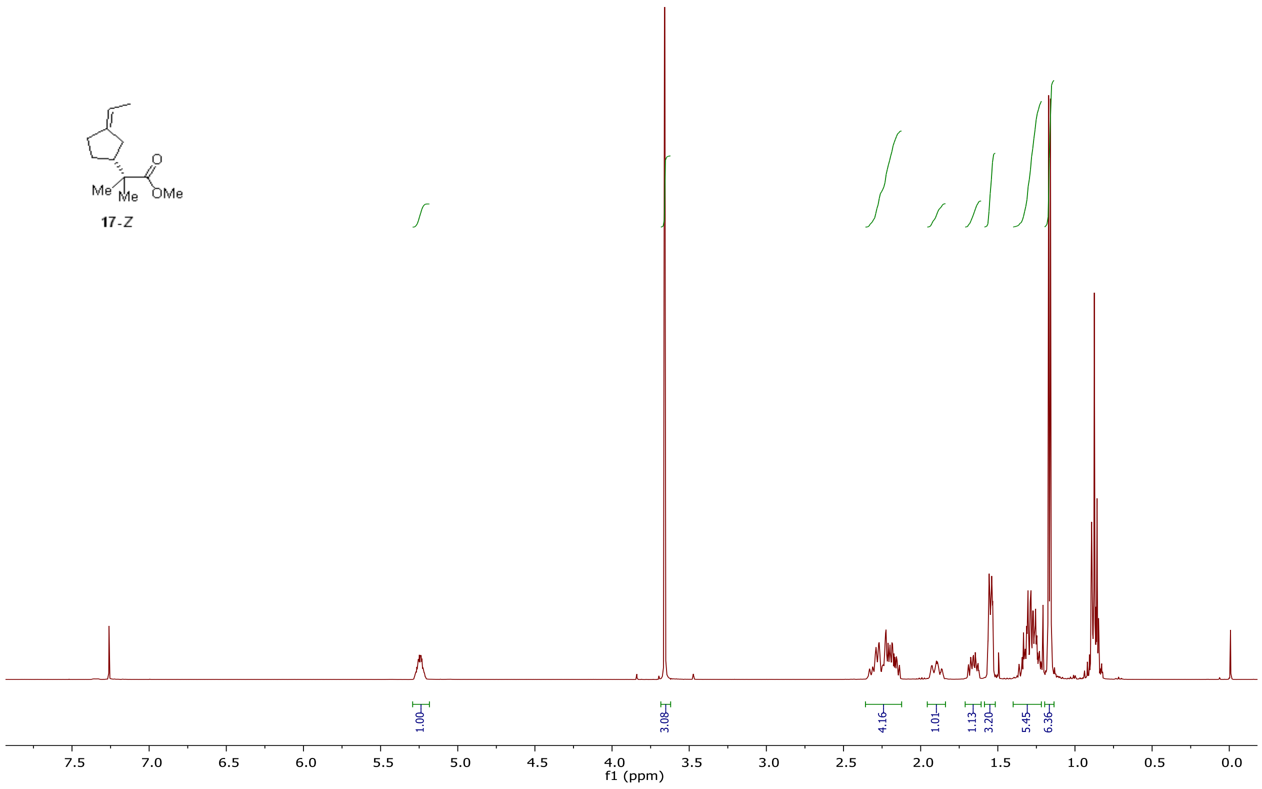

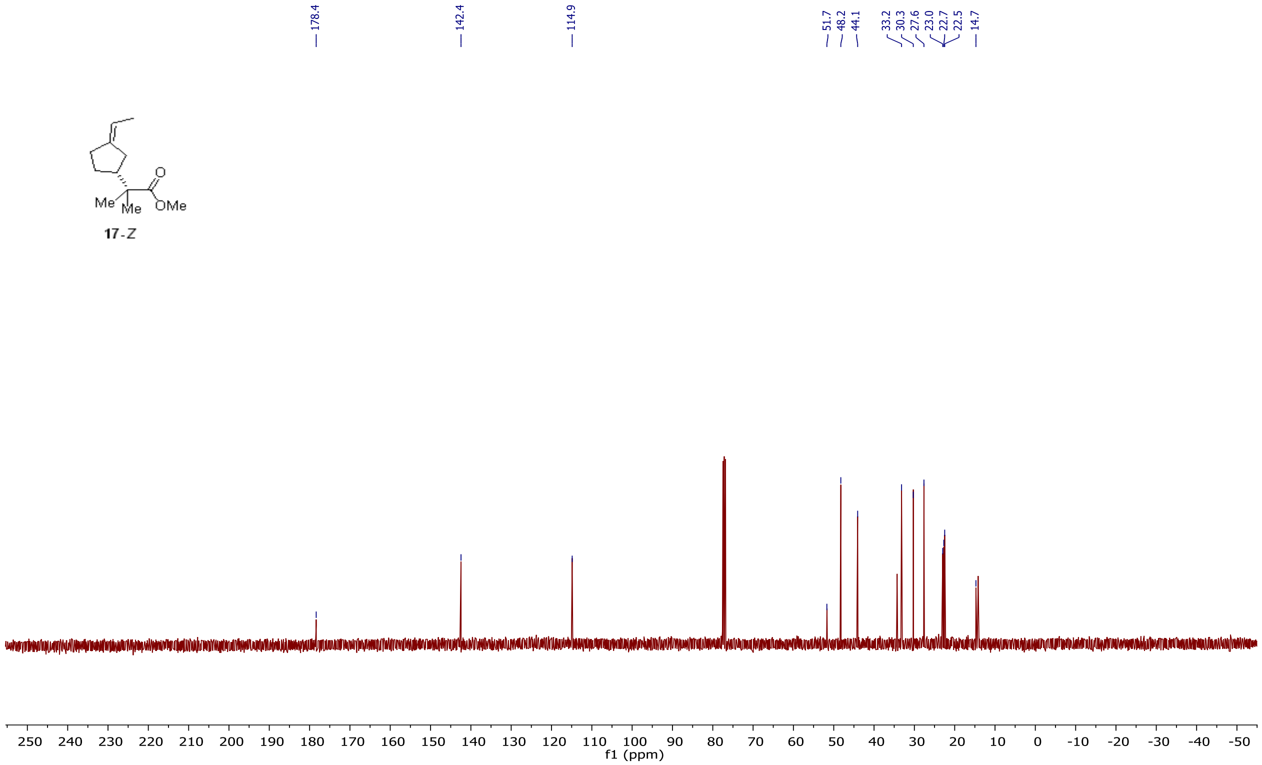

^{1}H NMR (500 MHz, CDCl$_3$) and ^{13}C NMR (125 MHz, CDCl$_3$) of 18-*E*

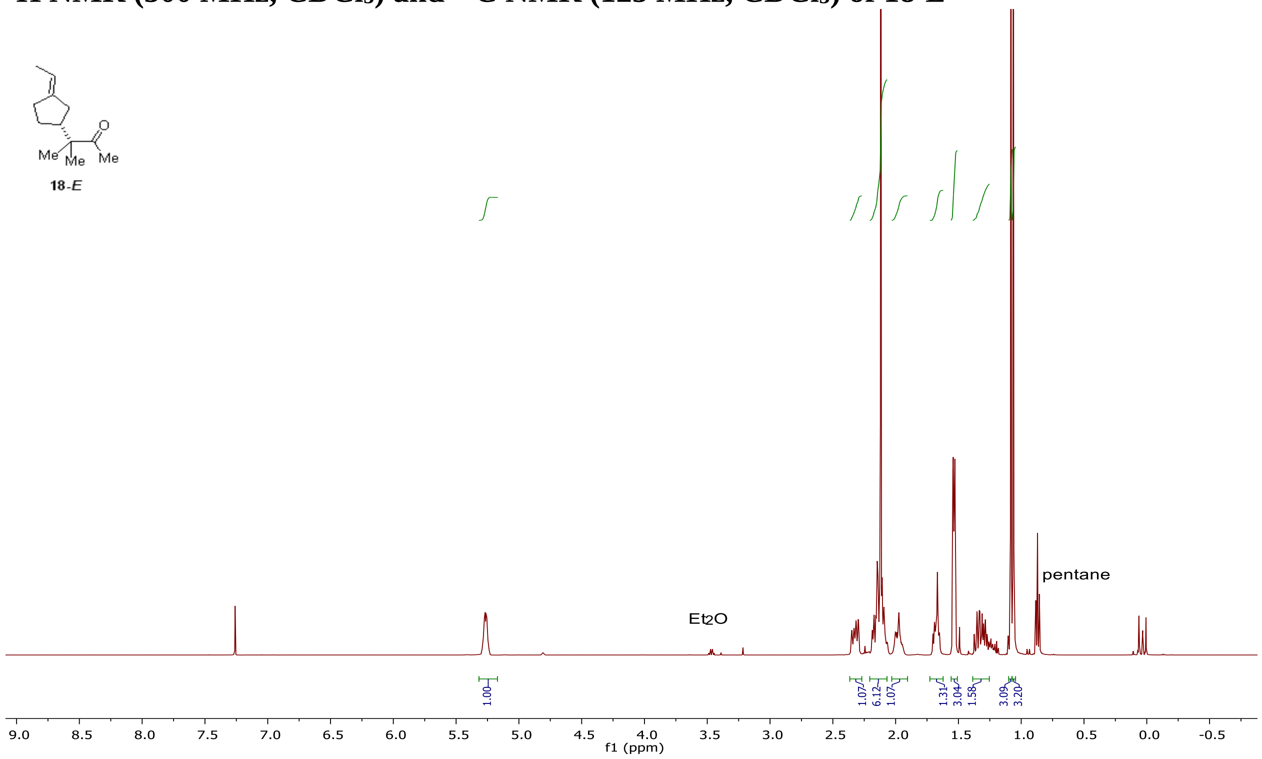

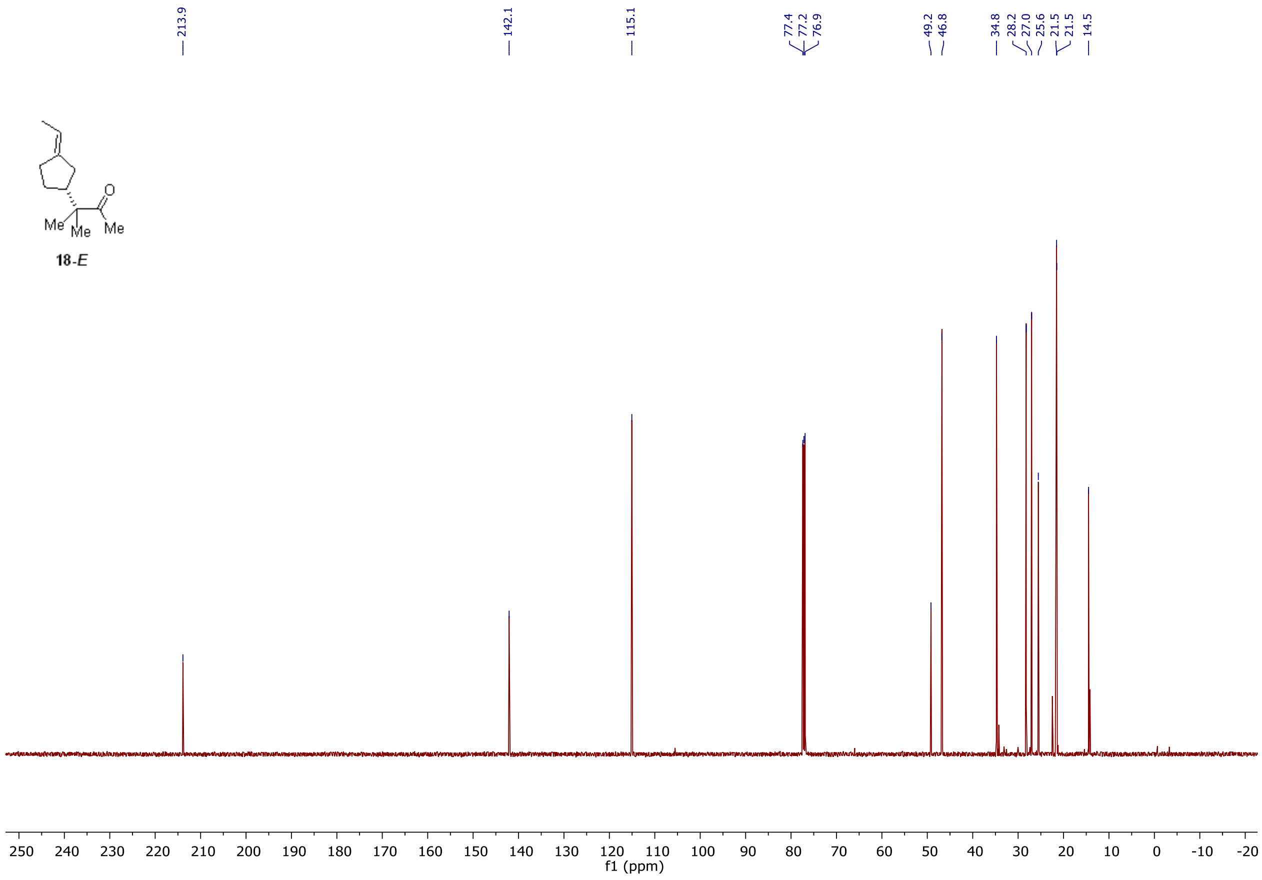

^{1}H NMR (500 MHz, CD_2Cl_2) and ^{13}C NMR (125 MHz, CD_2Cl_2) of 18-*Z*

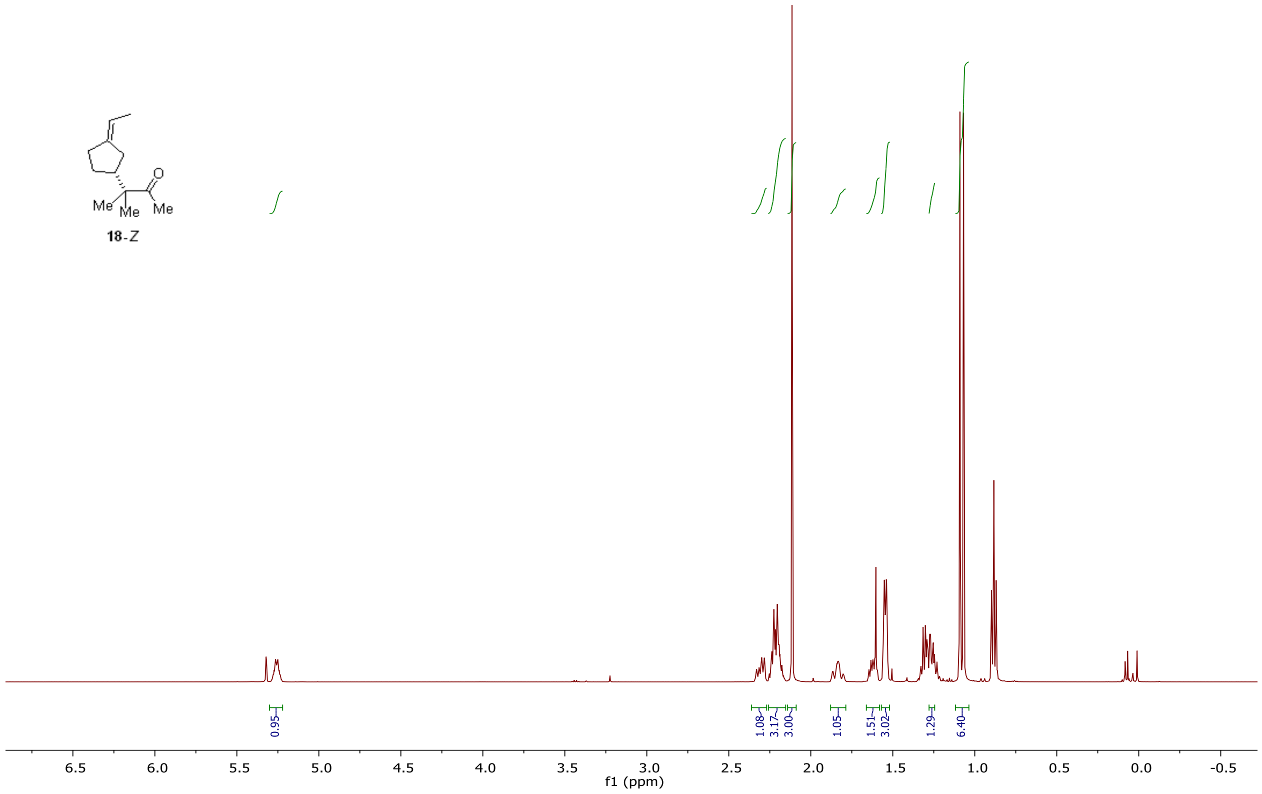

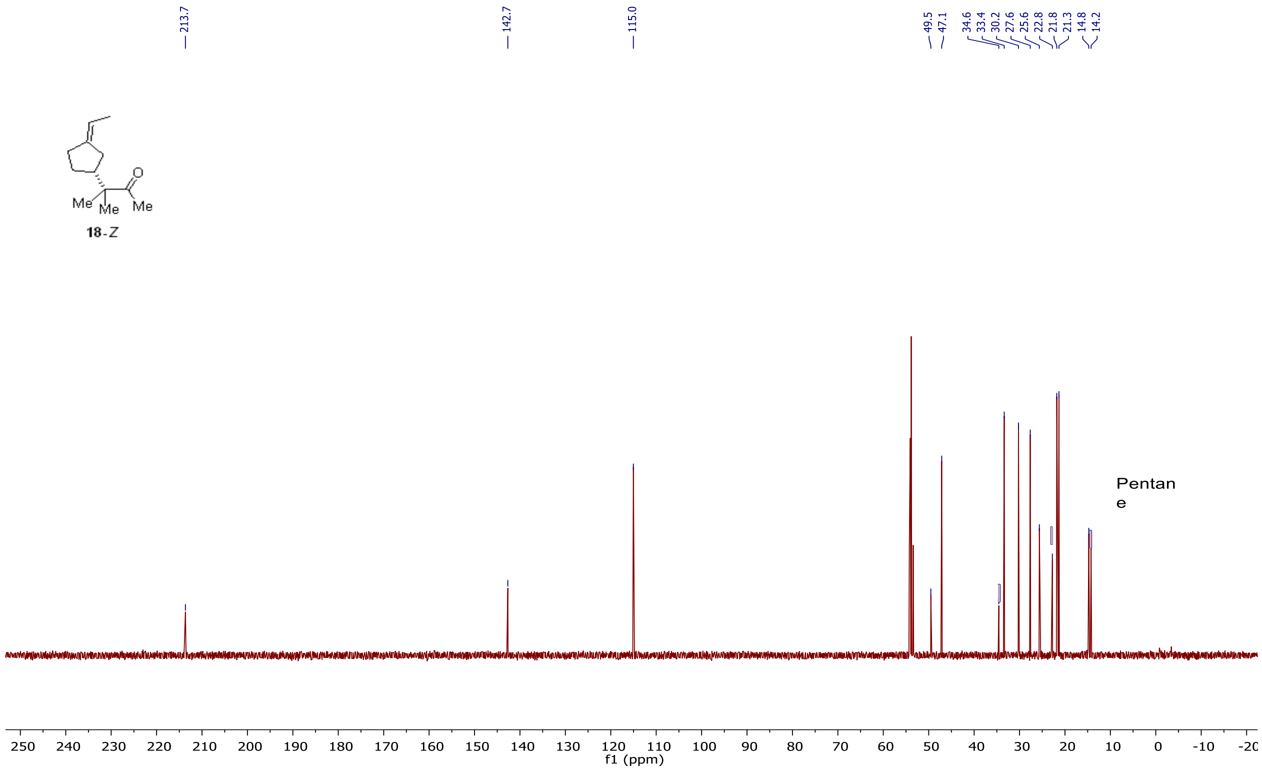

^{1}H NMR (500 MHz, $CDCl_3$) and ^{13}C NMR (125 MHz, $CDCl_3$) of 19-*E*

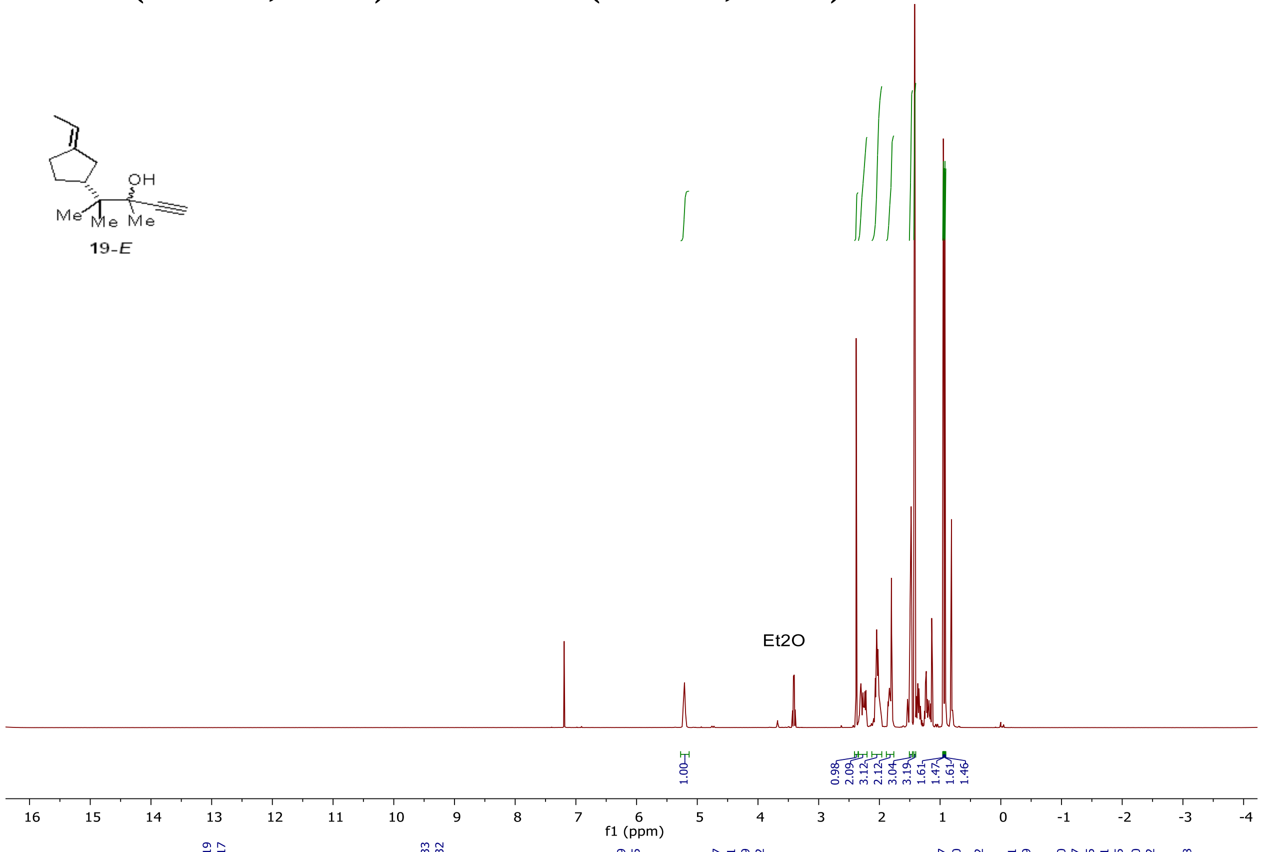

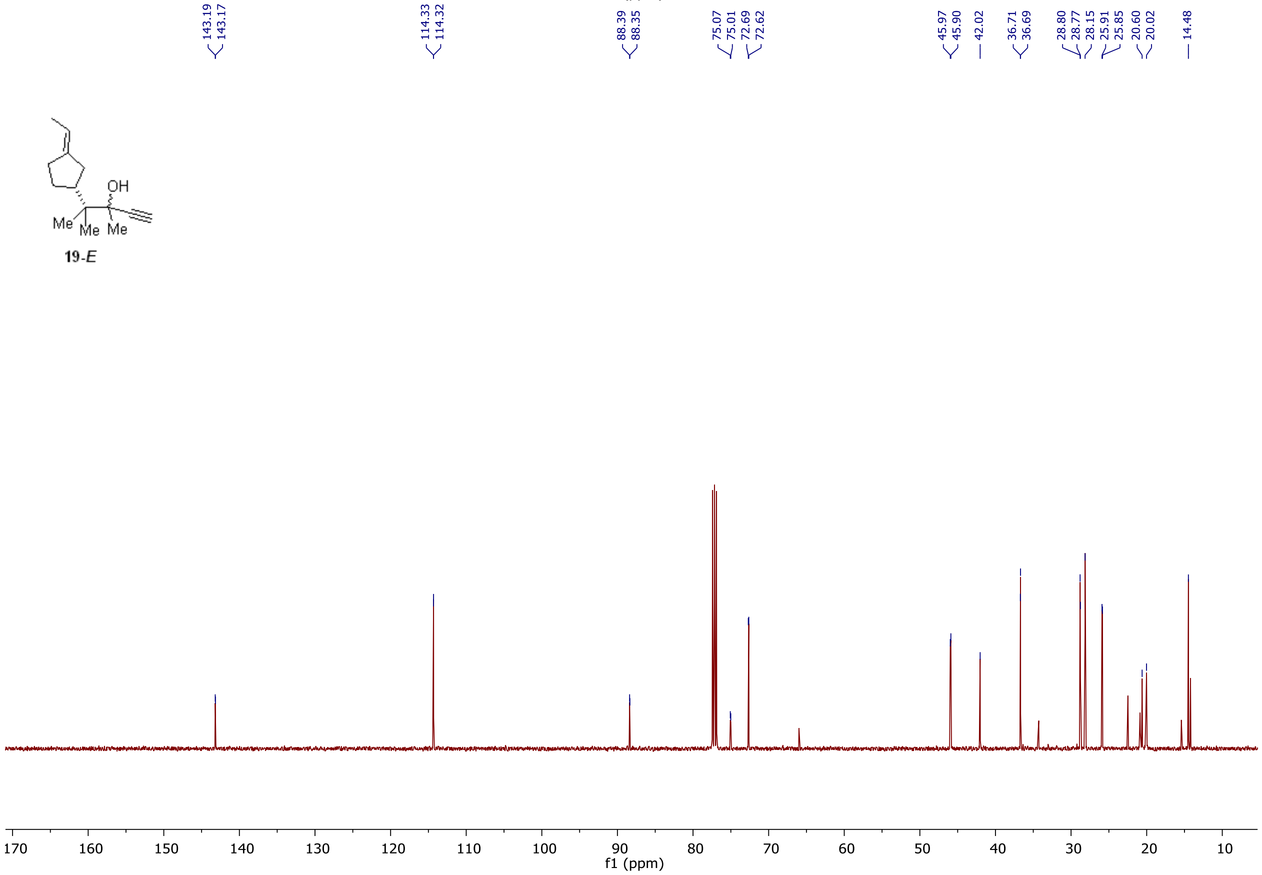

^{1}H NMR (500 MHz, CD_2Cl_2) and ^{13}C NMR (125 MHz, CD_2Cl_2) of 19-*Z*

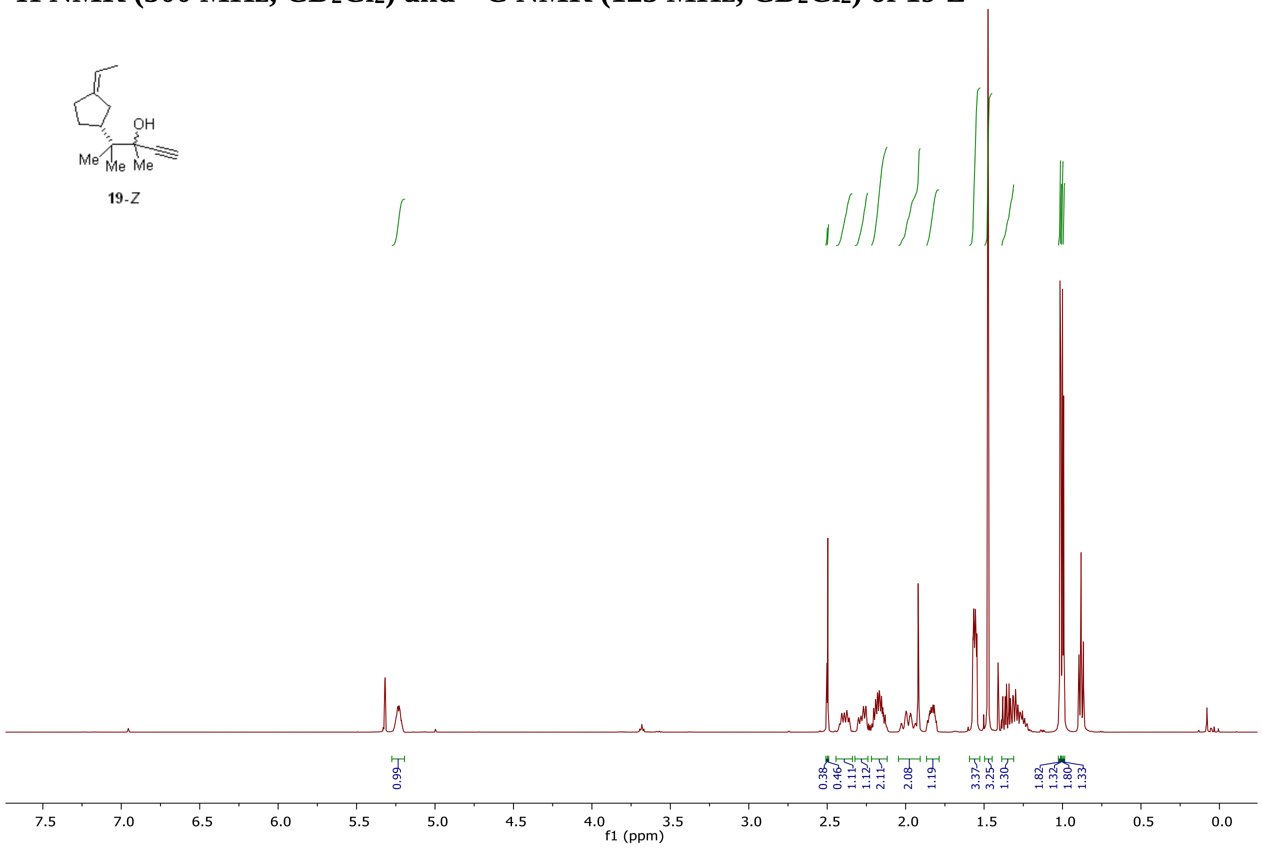

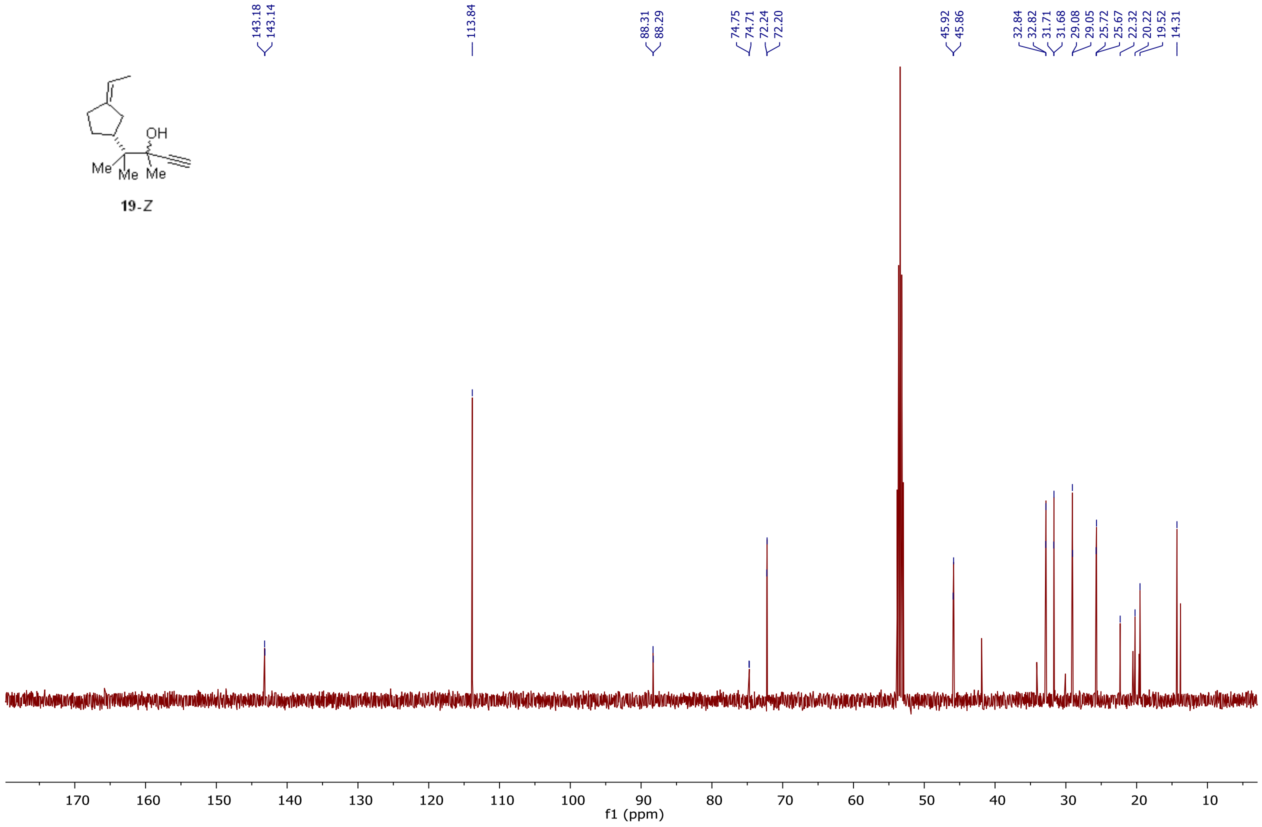

^{1}H NMR (500 MHz, CD_2Cl_2) and ^{13}C NMR (125 MHz, CD_2Cl_2) of 20-*E*

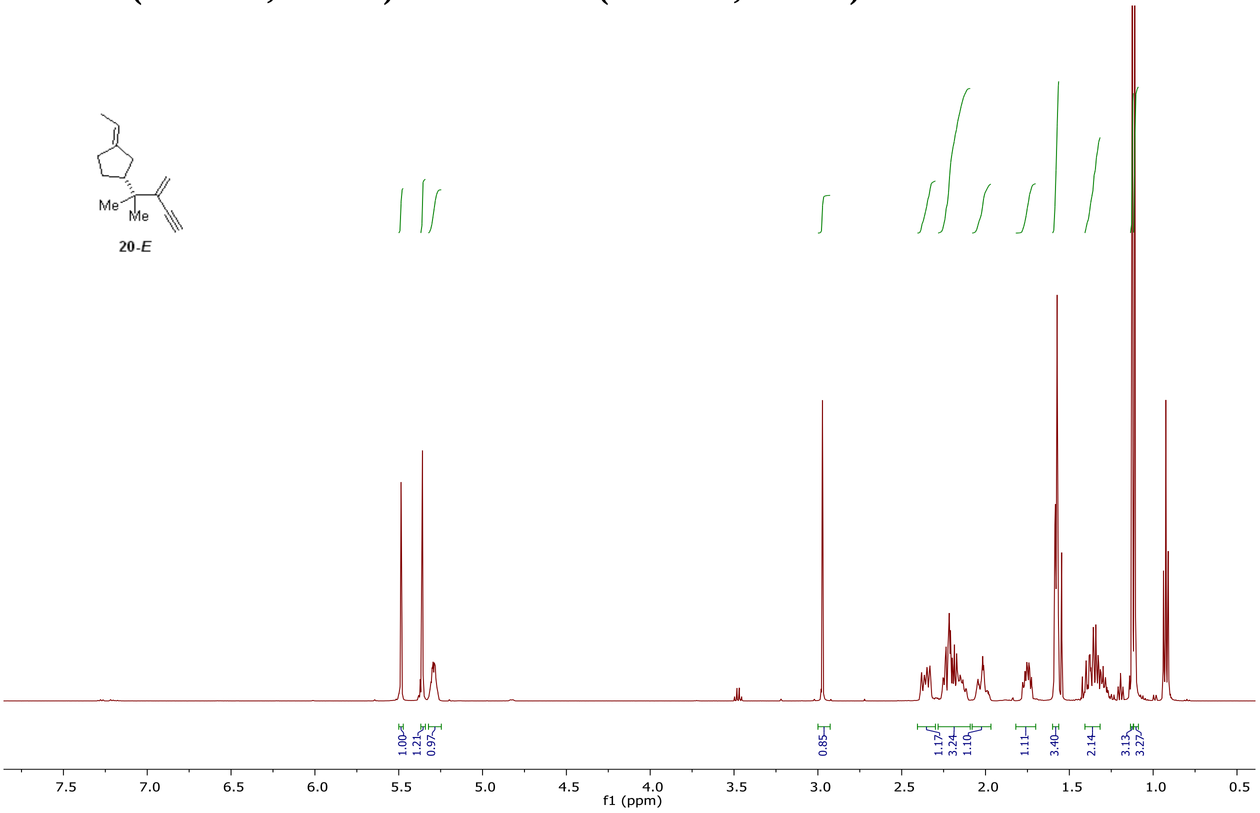

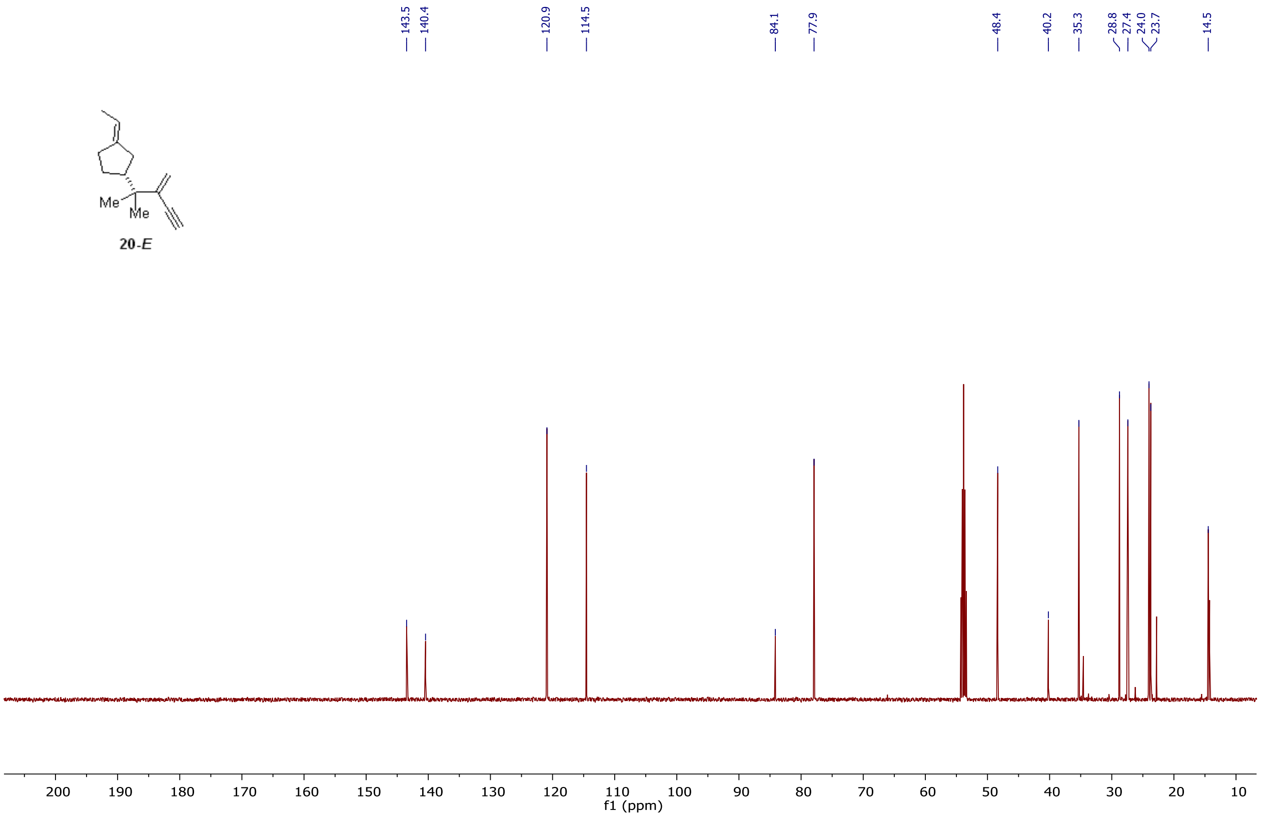

^{1}H NMR (500 MHz, CD_2Cl_2) and ^{13}C NMR (125 MHz, CD_2Cl_2) of 20-*E*

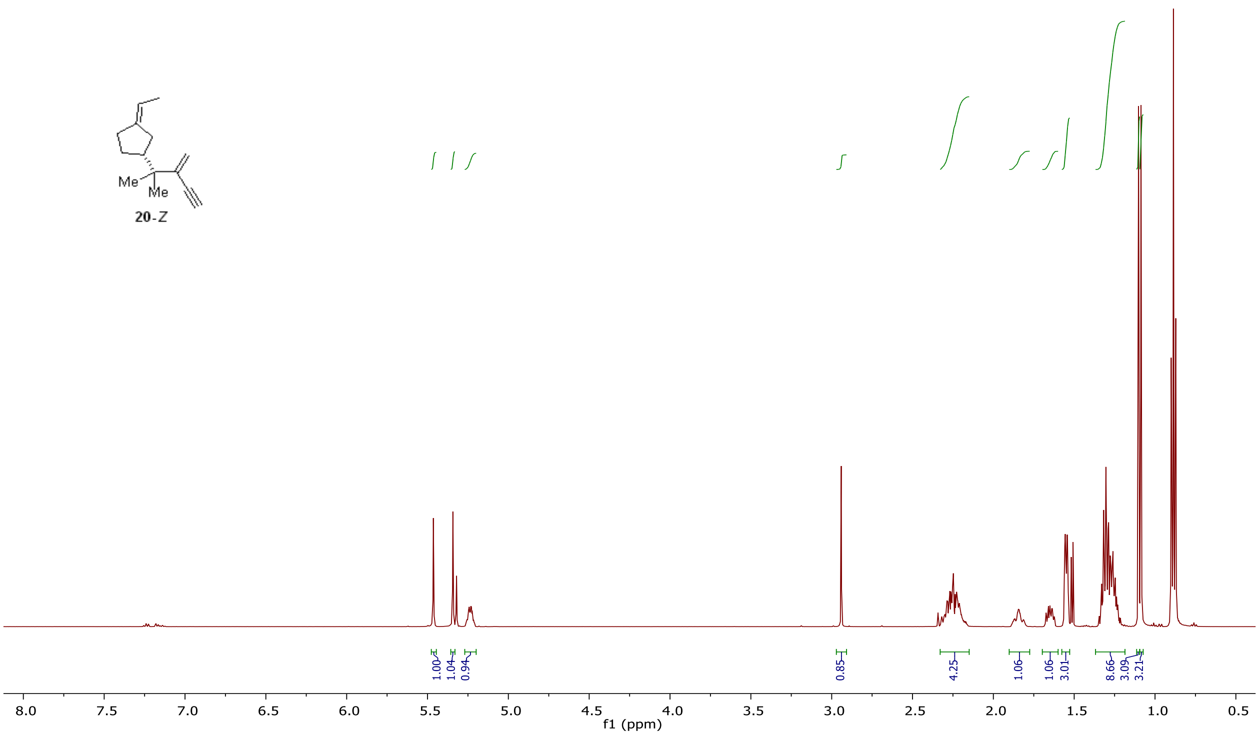

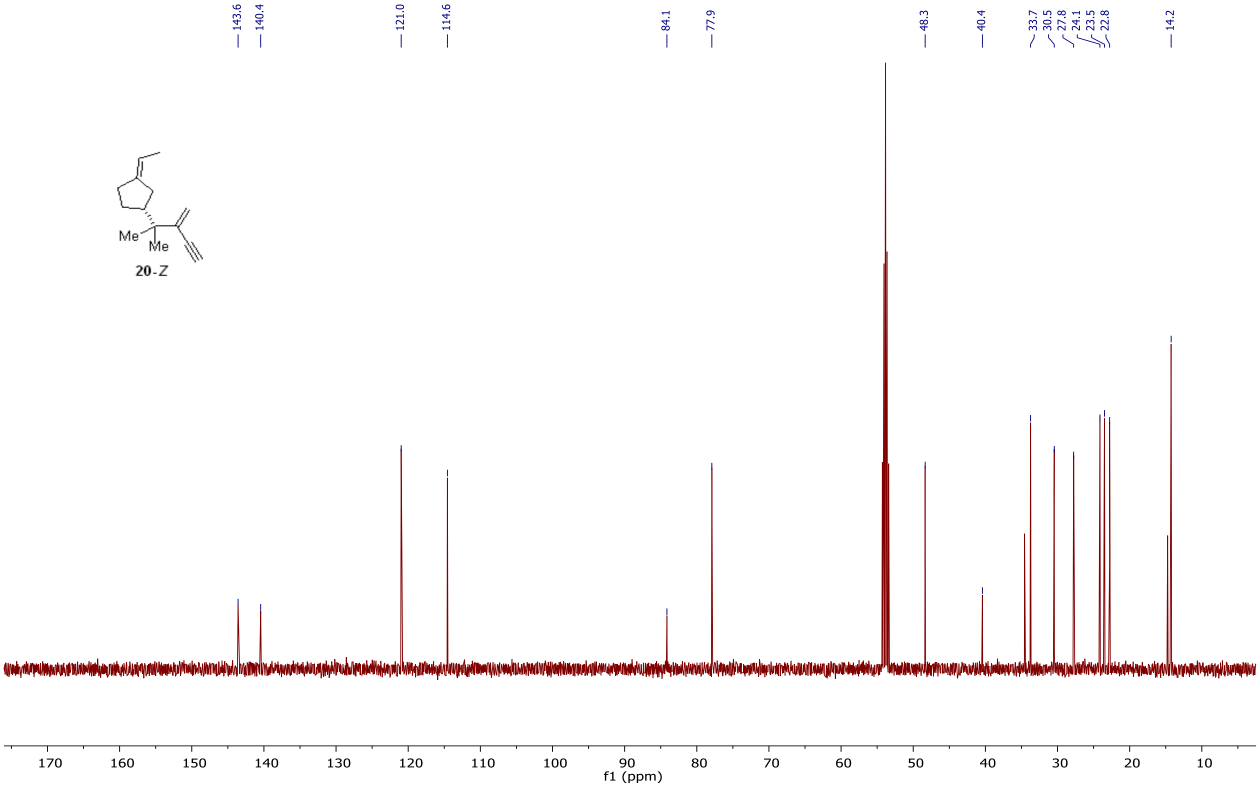

^{1}H NMR (500 MHz, $CDCl_3$) and ^{13}C NMR (125 MHz, CDC3) of 27

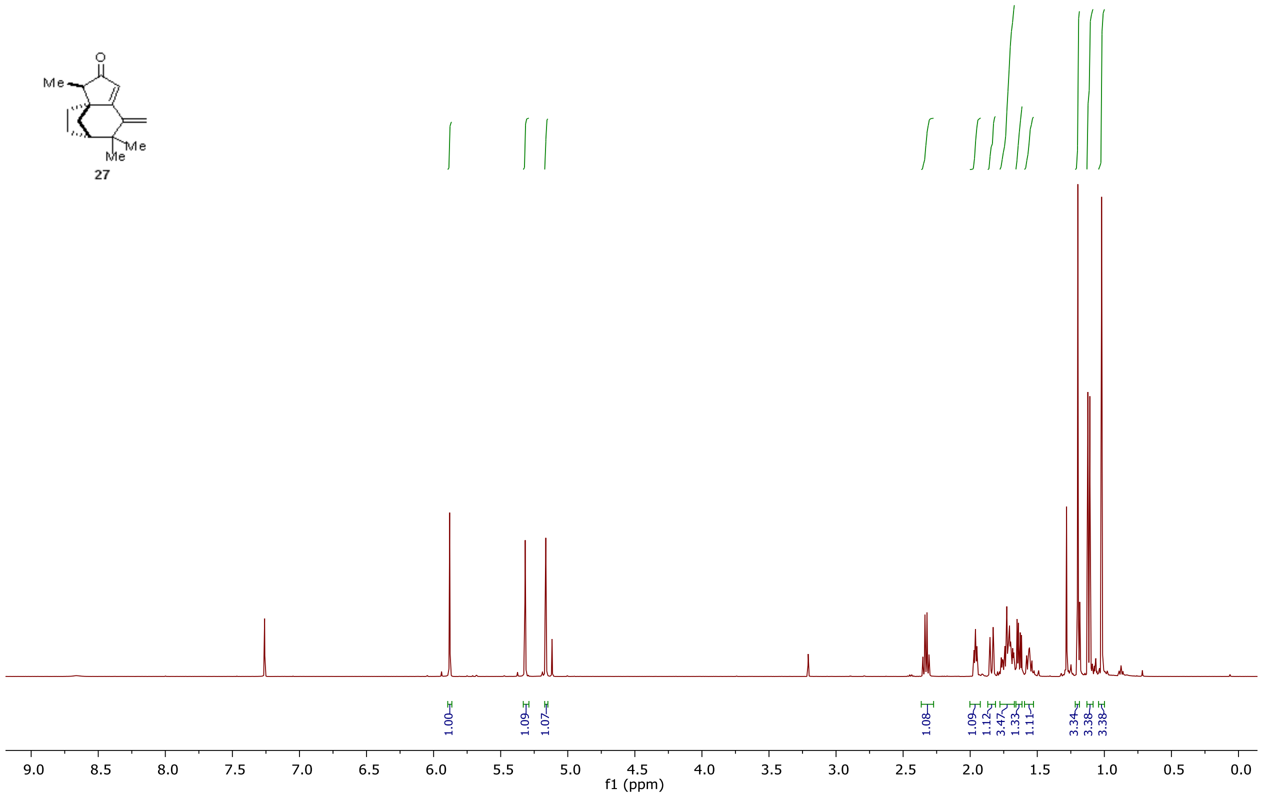

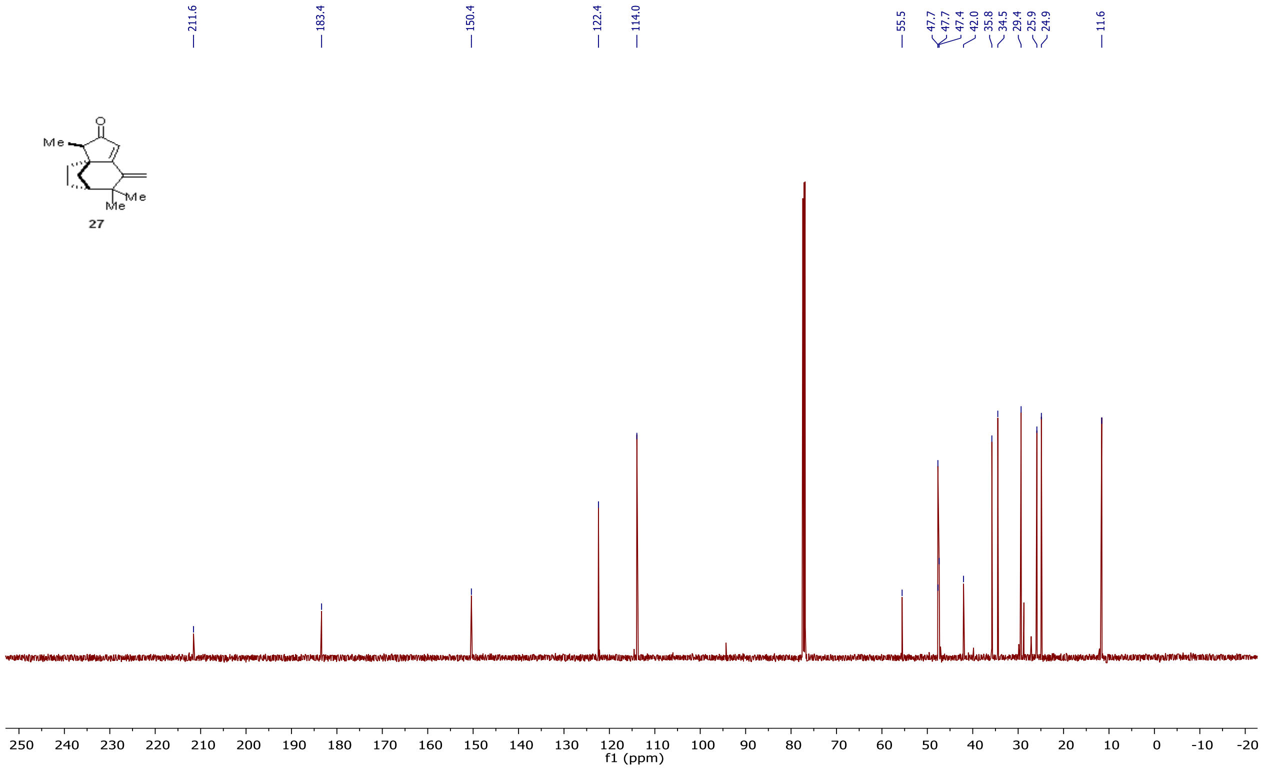

The overview of 2D NMR of 27

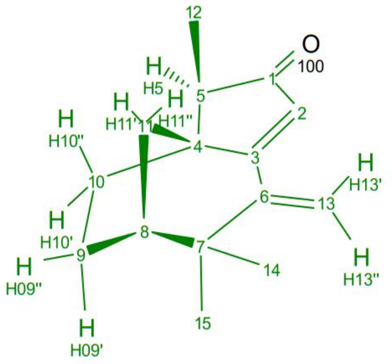

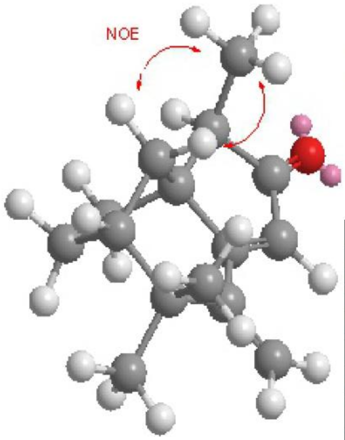

^{1}H NMR (600 MHz, Chloroform-*d*) δ 5.88 (s, 1H, 2), 5.32 (t, J = 0.9 Hz, 1H, H13'), 5.16 (d, J = 0.9 Hz, 1H, H13''), 2.33 (q, J = 7.6 Hz, 1H, H5), 1.96 (dd, J = 6.4, 4.8 Hz, 1H, 8), 1.84 (dt, J = 11.7, 2.1 Hz, 1H, H11''), 1.72 (m, 3H, H09', H09'', H10''), 1.63 (dd, J = 11.7, 4.9 Hz, 1H, H11'), 1.56 (m, 1H, H10'), 1.20 (s, 3H, 15), 1.12 (d, J = 7.6 Hz, 3H, 12), 1.02 (s, 3H, 14).

^{13}C NMR (151 MHz, Chloroform-*d*) δ 211.42, 183.23, 150.23, 122.25, 113.80, 55.38, 47.51, 47.22, 41.86, 35.63, 34.32, 29.18, 25.71, 24.72, 11.43.

Atom	δ (ppm)	J	COSY	HSQC	HMBC	NOESY
H5 H	2.33	7.60(12)	12	5	1, 4, 10, 12	12
H09' H	1.72		8	9		15
H09'' H	1.72		8	9		
H10'' H	1.72					H10'
H10' H	1.56					H10''
H11' H	1.64	4.90(8), -11.70(H11'')	H11'', 8	11	3, 4, 5, 8, 10	H11'', 8, 12
H11'' H	1.83	-11.70(H11')	H11', 8	11	9, 10	H11', 12, 14
H13' H	5.32	1.00(8), 1.00(H13'')	H13'', 8	13	3, 6, 7, 14	H13'', 2
H13'' H	5.16	1.00(H13')	H13'	13	3, 6, 7, 14	H13', 15
1 C	211.42				H5, 2, 12	
2 C	122.25			2		
H	5.88			2	1, 3, 4, 5, 6	H13'
3 C	183.23				H11', H13', H13'', 2	
4 C	55.38				H5, H11', 2, 8, 12	
5 C	47.22			H5	H11', 2, 12	
6 C	150.23				H13', H13'', 2, 8, 14, 15	
7 C	41.86				H13', H13'', 8, 14, 15	
8 C	47.47			8	H11', 14, 15	
H	1.96	1.00(H13'), 4.90(H11')	H09', H09'', H11', H11'', H13'	8	4, 6, 7, 10, 14, 15	H11', 14, 15
9 C	24.72			H09', H09''	H11''	
10 C	34.32				H5, H11', H11'', 8	
11 C	35.63			H11', H11''		
12 C	11.43			12	H5	
H3	1.12	7.60(H5)	H5	12	1, 4, 5	H5, H11', H11''
13 C	113.8			H13', H13''		
14 C	29.18			14	H13', H13'', 8, 15	
H3	1.02			14	6, 7, 8, 15	H11'', 8
15 C	25.71			15	8, 14	
H3	1.2			15	6, 7, 8, 14	H09', H13'', 8

P-ID: CF00xxx
Measured on: 31/10/2019
CHIFFRE: OUJ-OB-280-01
ELNA#: 3327
Client: Jie Ouyang
Group: List
Spectroscopist: Tobegen
Analysed on: 31/10/2019
Analysed by: Fares
Amount: 8 mg
Solvent: CDCl3
Reference: solvent
Temperature: 298 K
Spectrometer: AV600a
Probe: Z44896_0147 (CP TCI 600S3 H-C/N-D-05 Z)
Experiments: 1H-zg30, 13C-zgdc30, [13C, 1H]-hsqcedetgpsisp2.2, [1H, 1H]-cosygpmfphpp, [13C, 1H]-hmbcetgpl3nd, [1H, 1H]-noesygpph

HSQC of 27

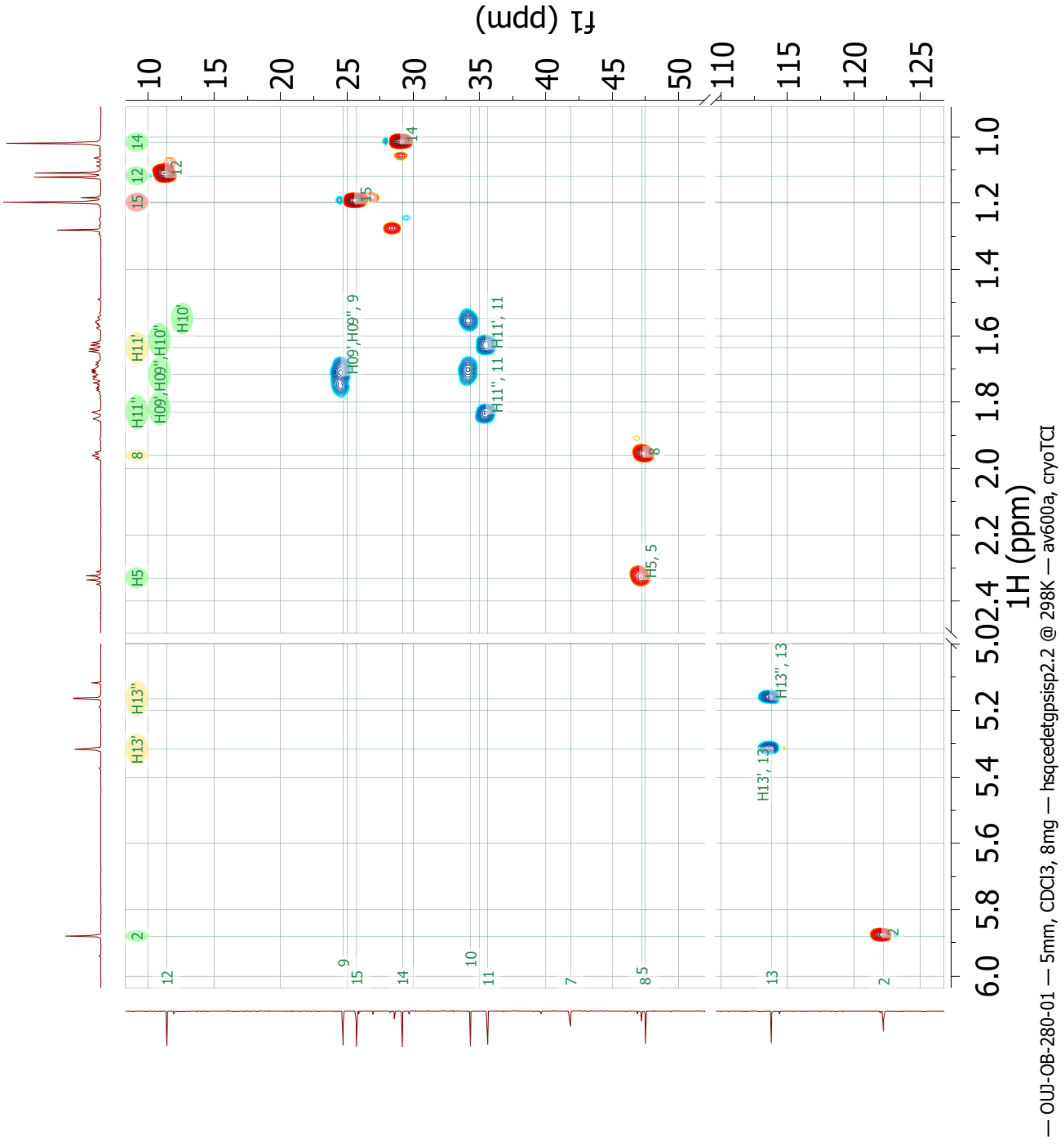
f1 (ppm)
1H (ppm)
— OUJ-OB-280-01 — 5mm, CDCl3, 8mg — hsqcedetgpsisp2.2 @ 298K — av600a, cryoTCI

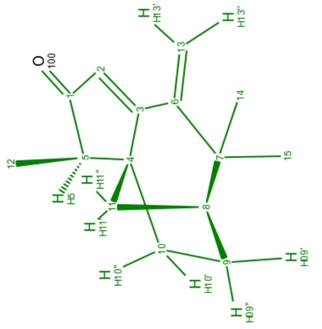

^{1}H-^{1}H COSY of 27

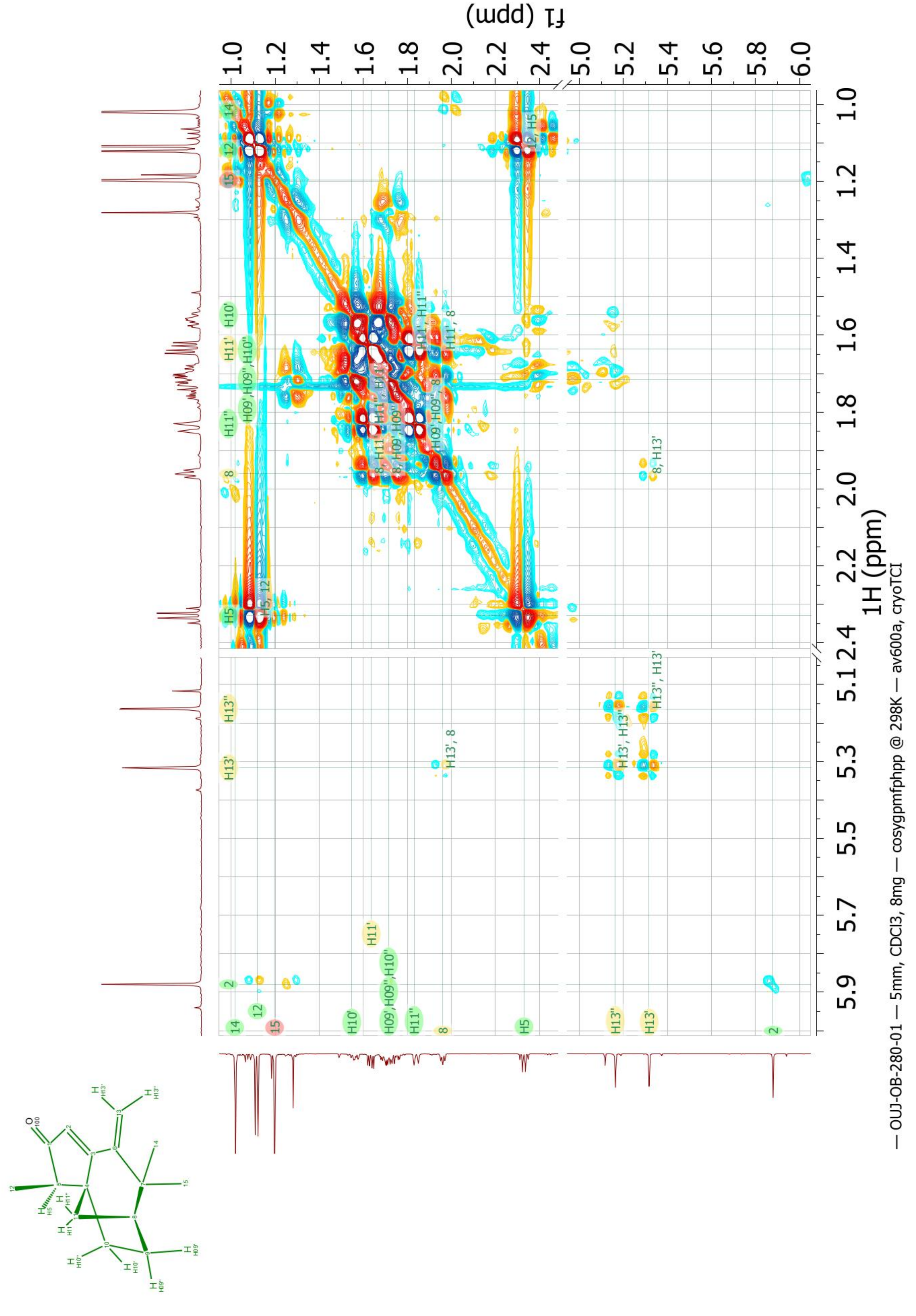

HMBC of 27

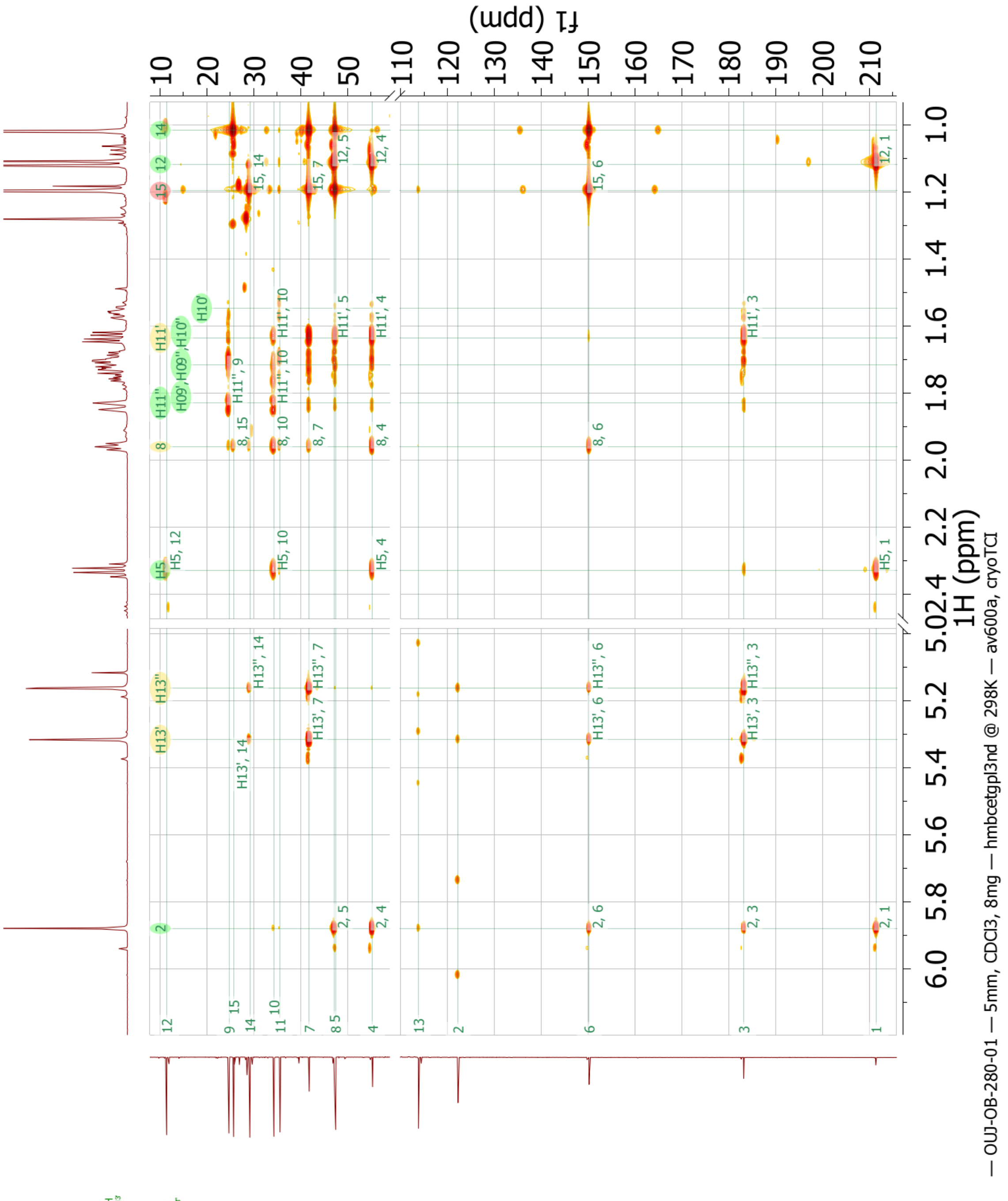
f1 (ppm)
1H (ppm)
— OUJ-OB-280-01 — 5mm, CDCl3, 8mg — hmbcetgpl3nd @ 298K — av600a, cryoTCI

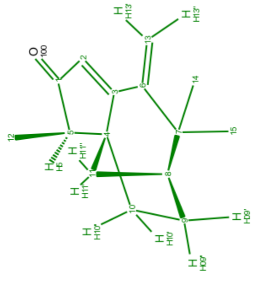

^{1}H-^{1}H NOESY of 27

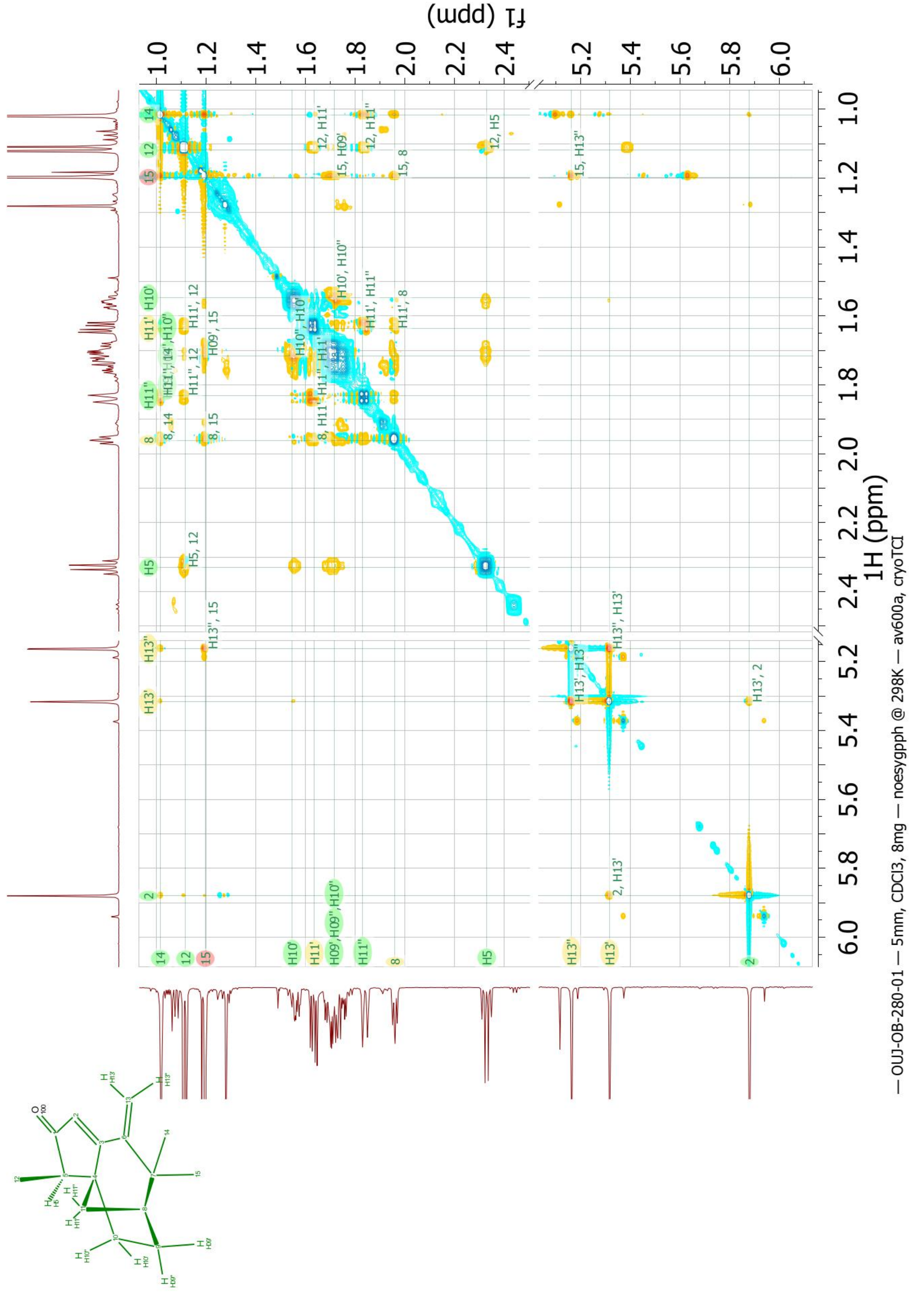

^{1}H NMR (500 MHz, $CDCl_3$) and ^{13}C NMR (125 MHz, CDC3) of 21

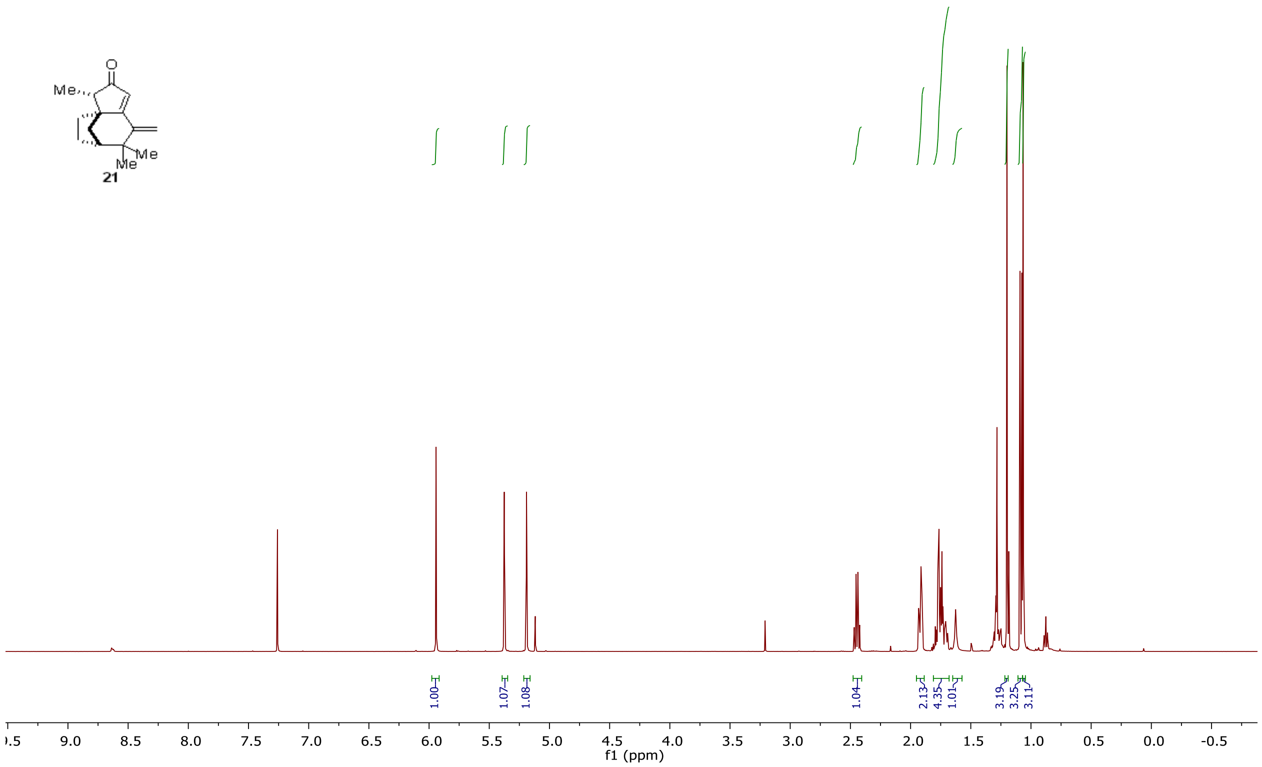

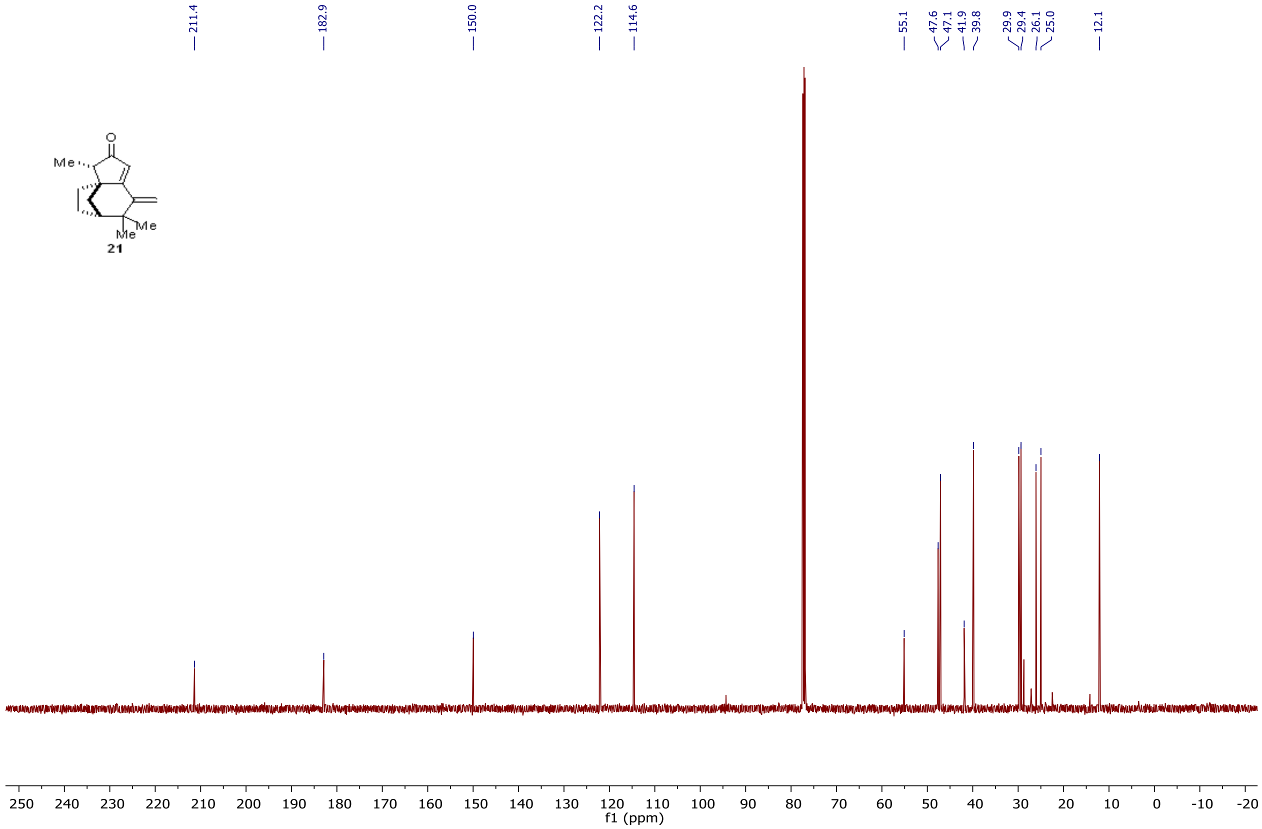

The 2D NMR overview of 21

^{1}H NMR (600 MHz, Chloroform-*d*) δ 5.94 (s, 1H, 2), 5.38 (s, 1H, H13'), 5.19 (d, *J* = 1.0 Hz, 1H, H13''), 2.45 (q, *J* = 7.6 Hz, 1H, H5), 1.92 (m, 2H, H11'', 8), 1.75 (m, 4H, H09', H09'', H11'), 1.29 (m, 1H), 1.20 (s, 3H, 15), 1.09 (d, *J* = 7.6 Hz, 3H, 12), 1.07 (s, 3H, 14).

^{13}C NMR (151 MHz, Chloroform-*d*) δ 211.21 , 1, 182.74 , 3, 149.80 , 6, 122.03 , 2, 114.40 , 13, 54.93 , 4, 47.45 , 5, 46.93 , 8, 41.72 , 7, 39.66 , 11, 29.70 , 10, 29.20 , 14, 25.90 , 15, 24.81 , 9, 11.93 , 12.

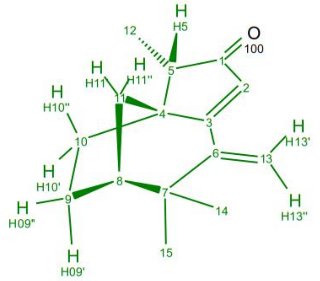

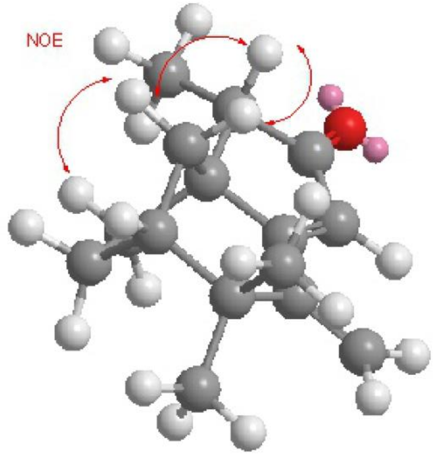

Atom	δ (ppm)	J	COSY	HSQC	HMBC	NOESY
H5 H	2.45	7.60(12)	12	5	1, 3, 4, 11, 12	H11', H11'', 12
H09' H	1.75			9		
H09'' H	1.75			9		
H10'' H	1.76		H10'	10		12
H10' H	1.27		H10''	10		
H11' H	1.75		H11''	11		H5
H11'' H	1.92		H11'	11		H5, 14
H13' H	5.38	1.00(H13'')	H13'', 8	13	2, 3, 6, 7	H13'', 2
H13'' H	5.19	1.00(H13')	H13'	13	2, 3, 6, 7	H13', 15
1 C	211.21				H5, 2, 12	
2 C	122.03			2	H13', H13''	
H	5.94			2	1, 3, 4, 5, 6	H13'
3 C	182.74				H5, H13', H13'', 2	
4 C	54.93				H5, 2, 12	
5 C	47.45			H5	2, 12	
6 C	149.8				H13', H13'', 2, 14, 15	
7 C	41.72				H13', H13'', 14, 15	
8 C	46.93			8	14, 15	
H	1.92		H13'	8		14, 15
9 C	24.81			H09', H09''		
10 C	29.7			H10'', H10'		
11 C	39.66			H11', H11''	H5	
12 C	11.93			12	H5	
H3	1.09	7.60(H5)	H5	12	1, 4, 5	H5, H10''
13 C	114.4			H13', H13''		
14 C	29.2			14	15	
H3	1.07			14	6, 7, 8, 15	H11'', 8
15 C	25.9			15	14	
H3	1.2			15	6, 7, 8, 14	H13'', 8
100 O						

P-ID: CF00xxx
Measured on: 02/11/2019
CHIFFRE: OUJ-OB-280-02
ELNA#: 3328
Client: Jie Ouyang
Group: List
Spectroscopist: Tobegen
Analysed on: 02/11/2019
Analysed by: Fares
Amount: 9 mg
Solvent: CDCl3
Reference: solvent
Temperature: 298 K
Spectrometer: AV600a
Probe: Z44896_0147 (CP TCI 600S3 H-C/N-D-05 Z)
Experiments: 1H-zg30, 13C-zgdc30, [13C, 1H]-hsqcedetgpsisp2.2, [1H, 1H]-cosygpmfphpp, [13C, 1H]-hmbcetgpl3nd, [1H, 1H]-noesygpph

HSQC of 21

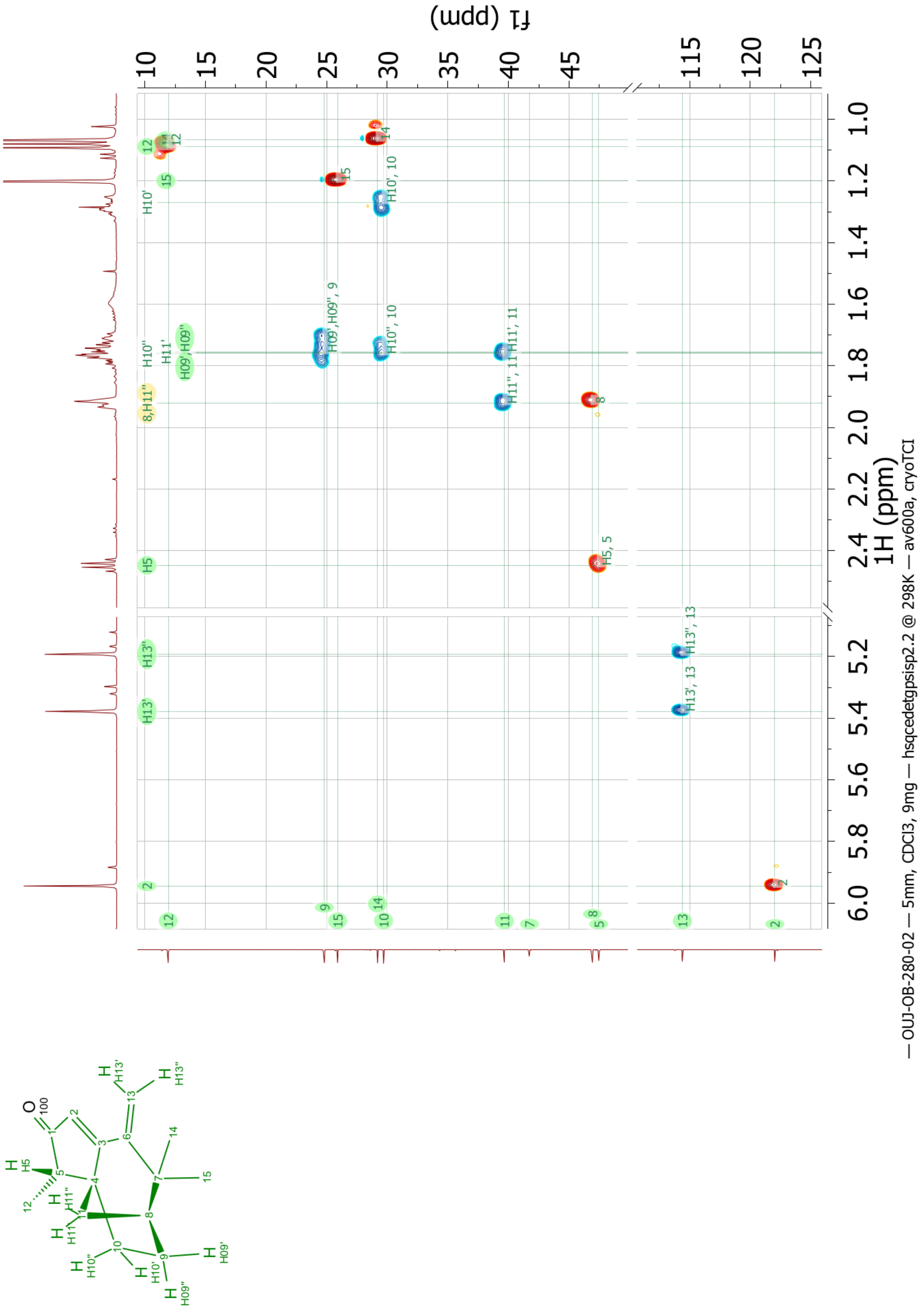
f1 (ppm)
1H (ppm)
— OUJ-OB-280-02 — 5mm, CDCl3, 9mg — hsqcedetgpsisp2.2 @ 298K — av600a, cryoTCI

^{1}H-^{1}H COSY of 21

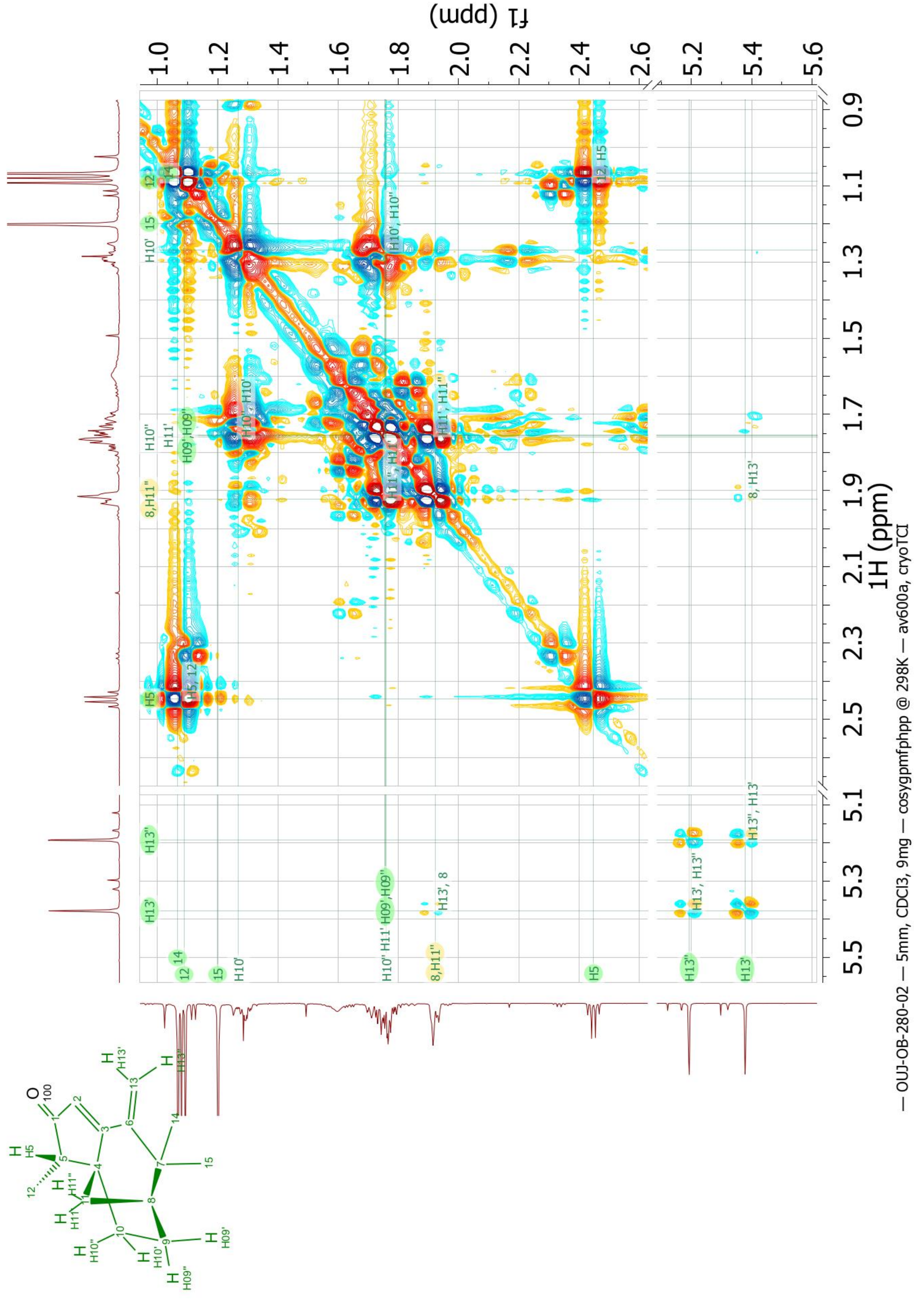

HMBC of 21

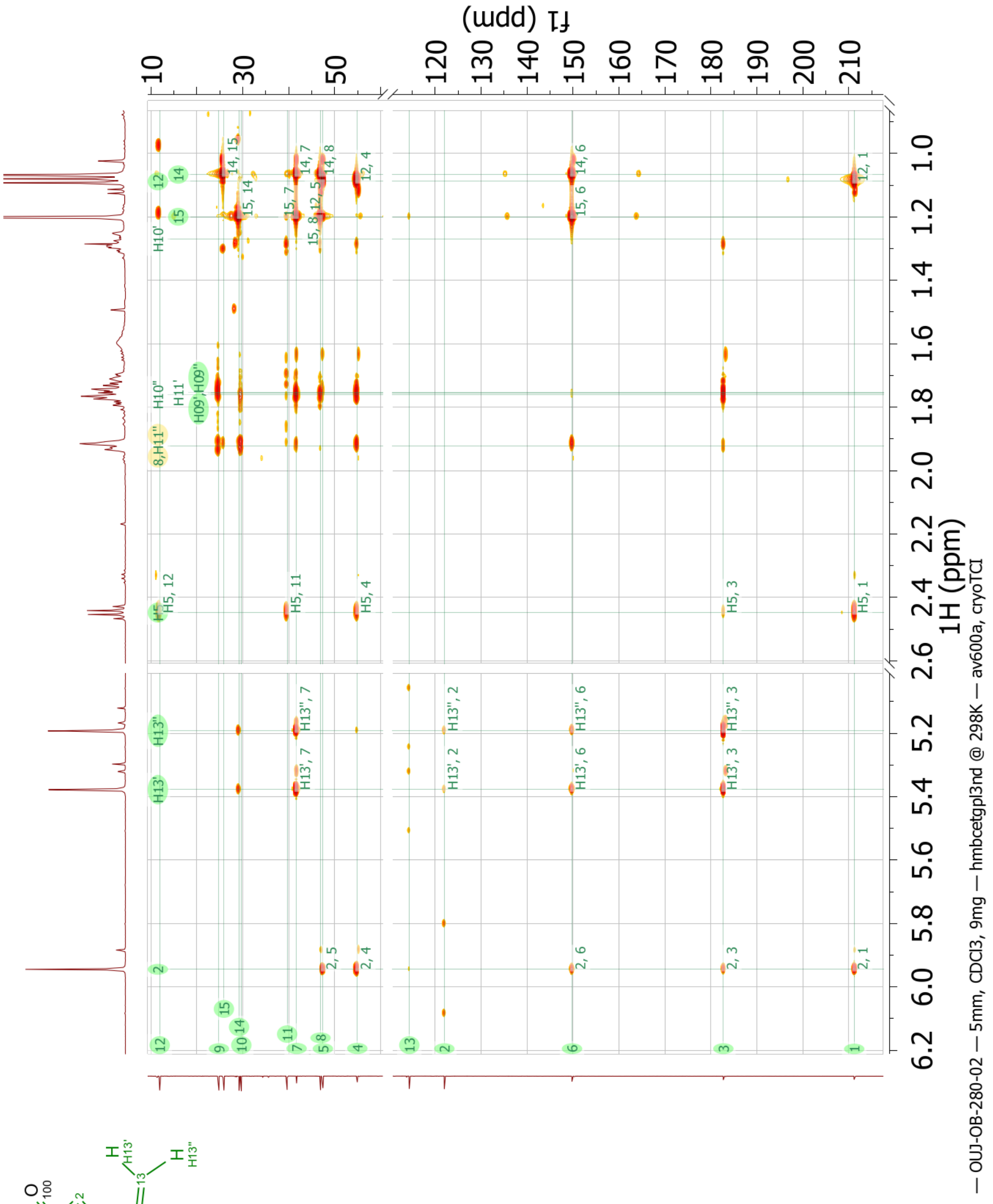
f1 (ppm)
1H (ppm)
— OUJ-OB-280-02 — 5mm, CDCl3, 9mg — hmbcetgpl3nd @ 298K — av600a, cryoTCI

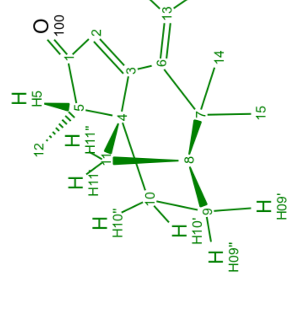

^{1}H-^{1}H NOESY of 21

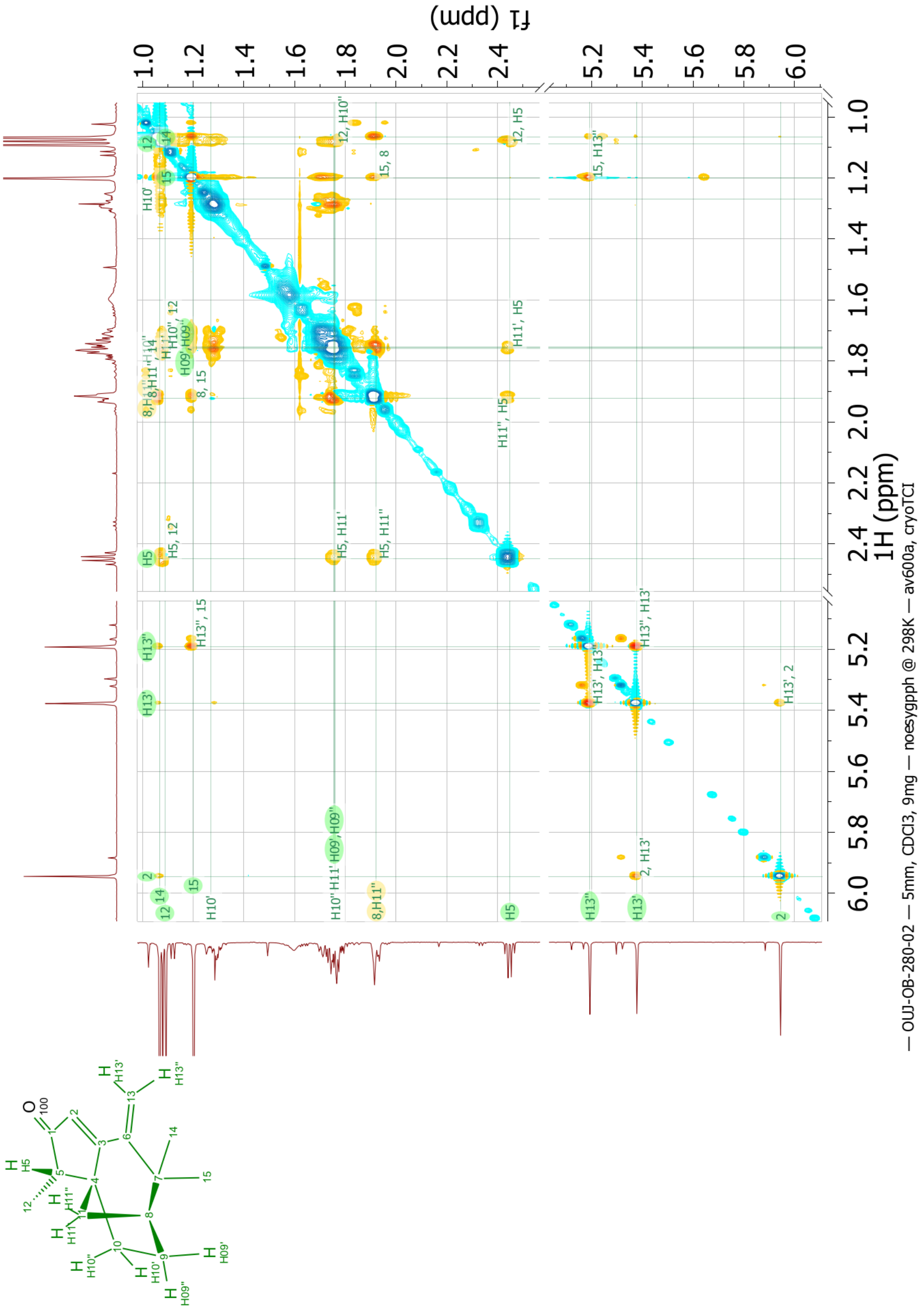

^{1}H NMR (500 MHz, $CDCl_3$) and ^{13}C NMR (125 MHz, $CDCl_3$) of 22

22

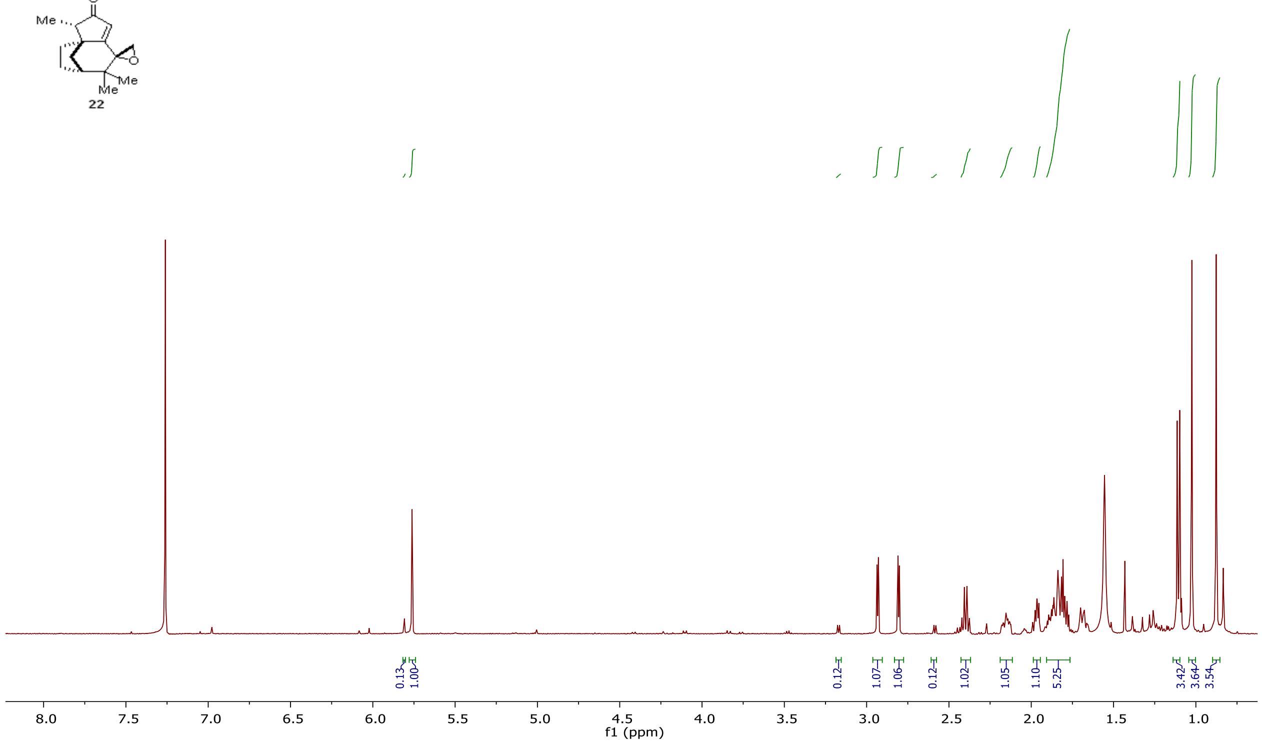

22

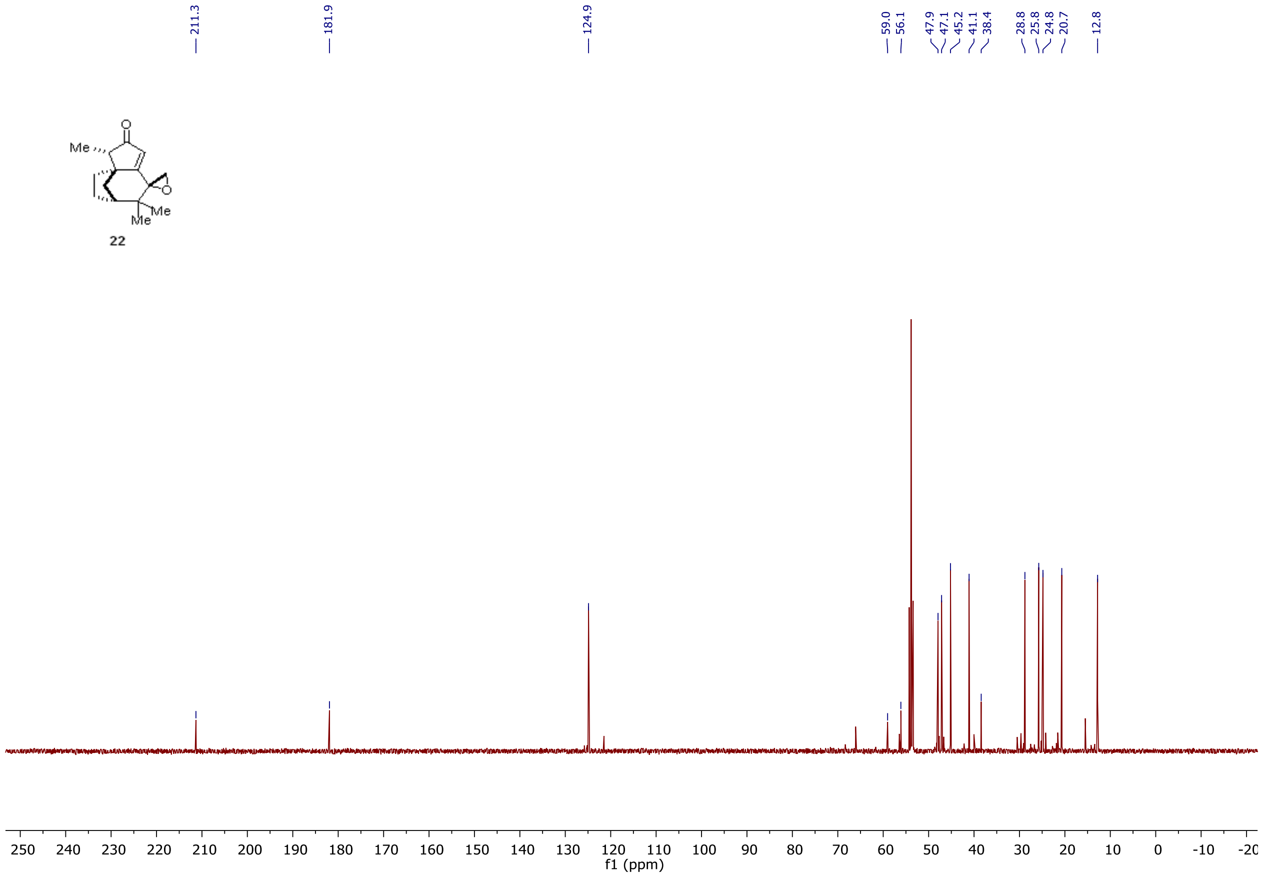

^{1}H NMR (500 MHz, $CDCl_3$) and ^{13}C NMR (125 MHz, $CDCl_3$) of 23

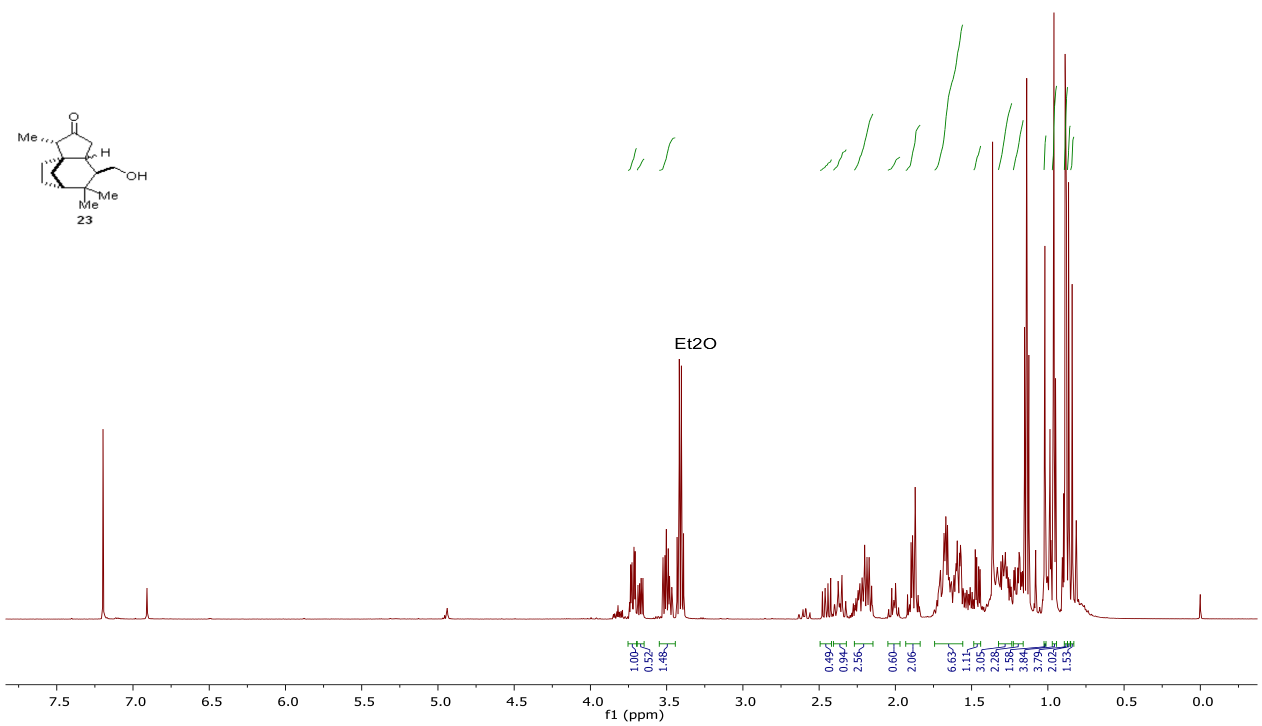

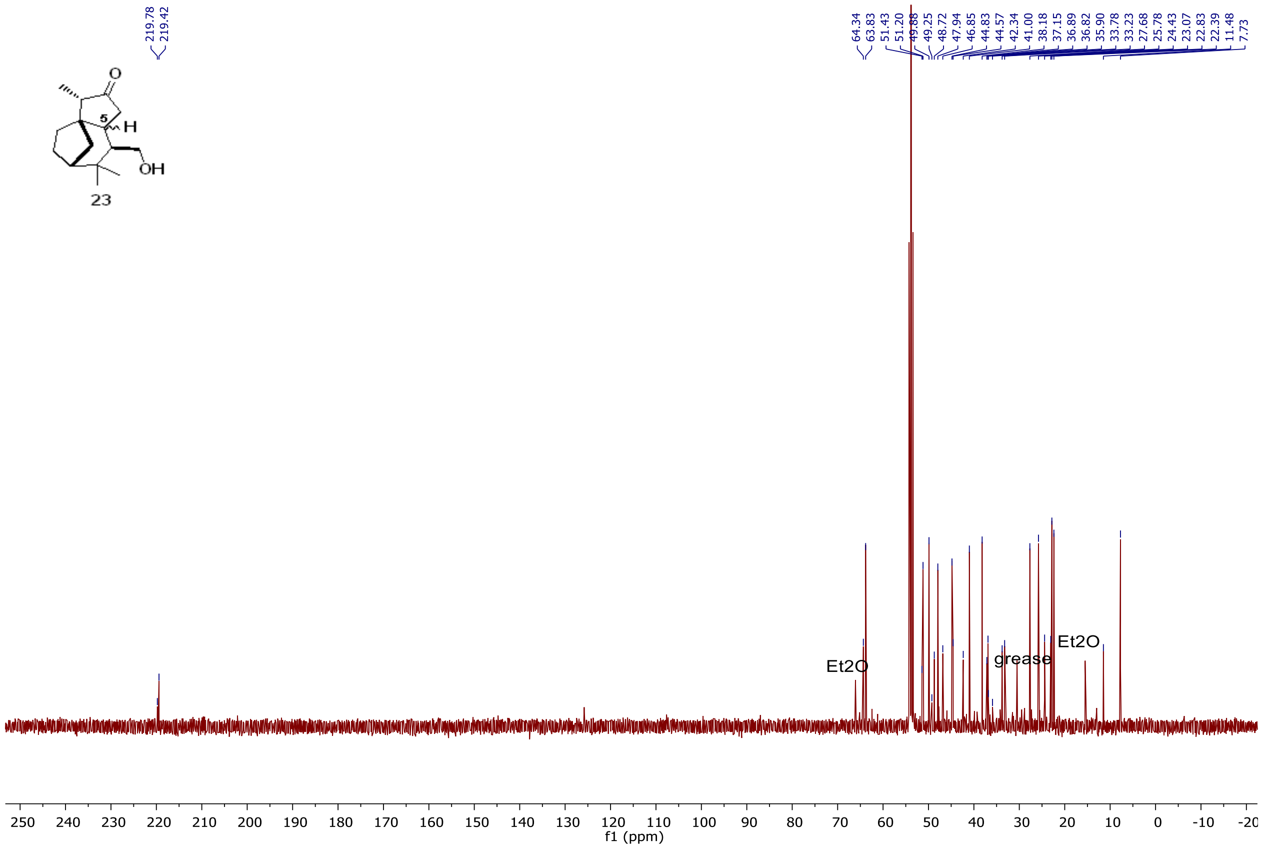

^{1}H NMR (500 MHz, CDCl$_3$) and ^{13}C NMR (125 MHz, CDCl$_3$) of 10

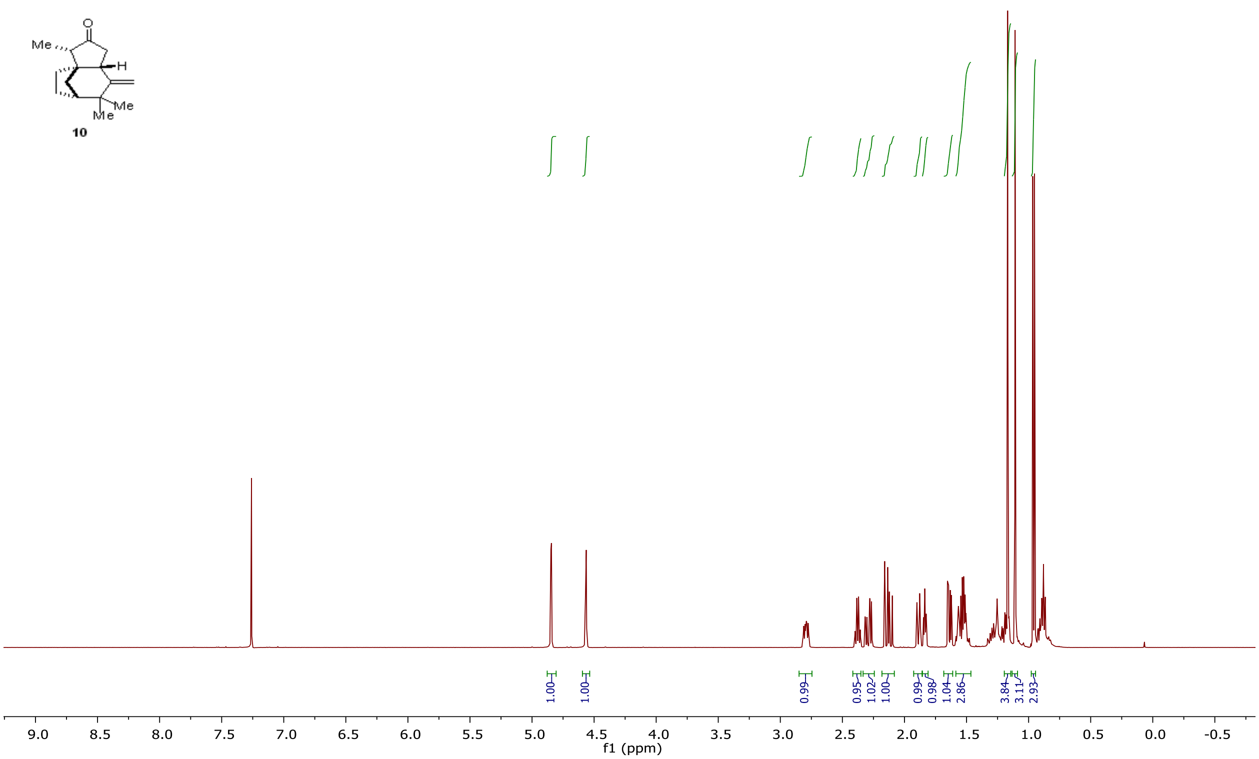

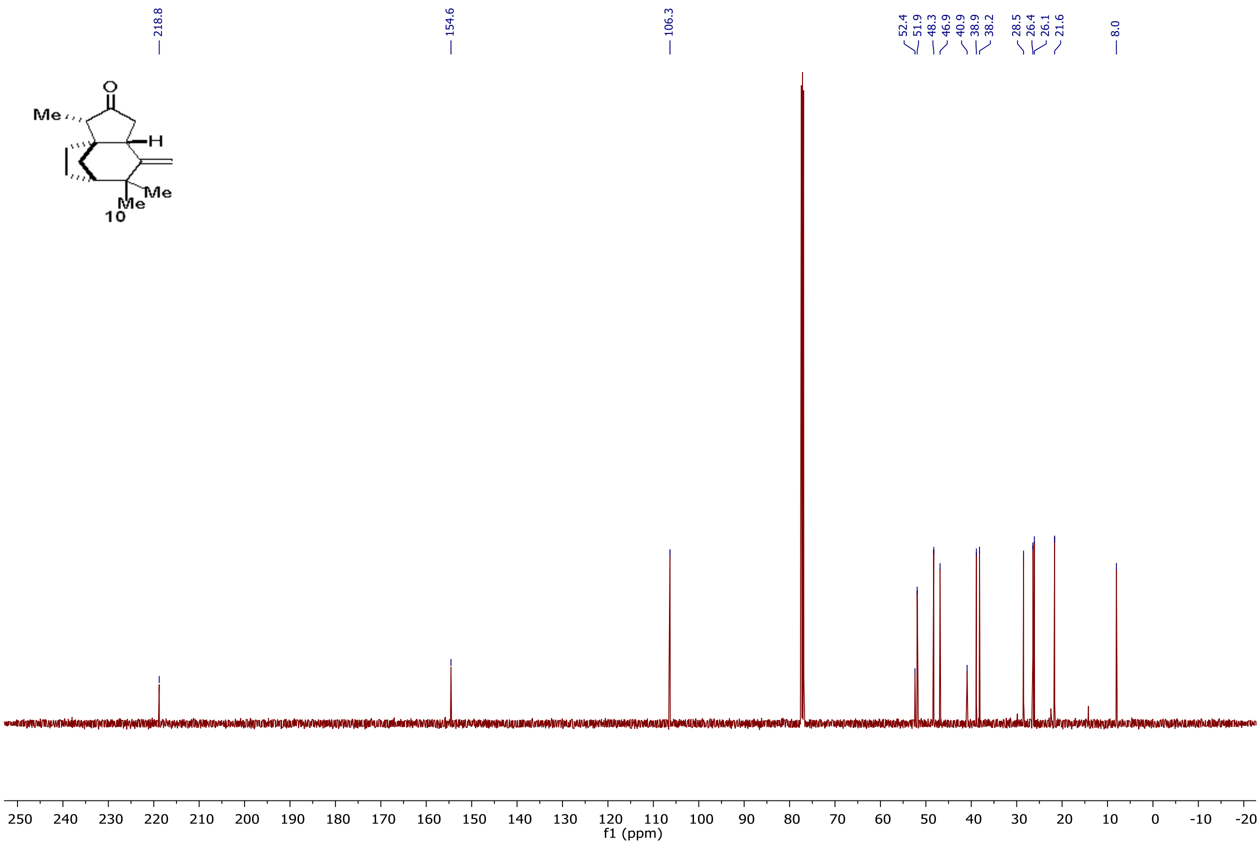

The 2D NMR overview analysis of 10

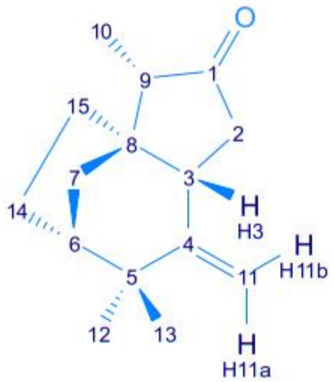

OUJ-OB-319

AV500as; 298 K; 5 mg; $CDCl_3$

equivalent to OUJ-OB-163 (CD_2Cl_2)

Atom	δ	J	COSY	HSQC	HMQC	NOESY
C1	218.67				2a, 2b, 9, 10	
C2	38.02			2a, 2b	7b	
H2a	2.291	d 18.5(2b), d 7.4(3), d 1.3(9)	H3, 2b, 9	2	1, 3, 8	H11b
H2b	2.129	d 18.5(2a), d 12.1(3)	H3, 2a	2	1, 3, 4	H11b, 15b
C3	46.69			H3	H11a, H11b, 2a, 2b, 7a, 7b, 15a, 15b	
H3	2.794	d 12.1(2b), d 7.4(2a), q 2.0(?)	H11a, H11b, 2a, 2b, 15a	3	4, 15	7a, 9, 13
C4	154.40				H3, 2b, 6, 12, 13	
C5	40.74				H11a, H11b, 7a, 7b, 12, 13, 14a	
C6	48.09			6	7b, 12, 13	
H6	1.837	d 5.8(14a), d 4.7(7b)	7b, 14b	6	4, 8, 15	12, 13
C7	38.70			7a, 7b	9, 15b	
H7a	1.890	d 11.4(7b), t 2.1(14b, 15b)	7b, 14b, 15b	7	3, 5, 14, 15	H3, 9, 13
H7b	1.638	d 11.4(7a), d 4.7(6)	6, 7a	7	2, 3, 5, 6, 8, 15	9, 10
C8	52.23				2a, 6, 7b, 9, 10	
C9	51.74			9	10	
H9	2.378	q 7.0(10), d 1.3(2a)	2a, 10	9	1, 7, 8, 10, 15	H3, 7a, 7b
C10	7.88			10	9	
H10	0.960	d 7.0(9)	9	10	1, 8, 9	7b, 15a
C11	106.18			H11a, H11b		
H11a	4.846	d 2.2(?), d 0.9(?)	H3, H11b	11	3, 5	H11b, 12
H11b	4.566	d 2.2(?)	H3, H11a	11	3, 5	H11a, 2a, 2b
C12	26.27			12	13	
H12	1.109	s	13	12	4, 5, 6, 13	H11a, 6, 14b
C13	28.31			13	12	
H13	1.169	s	12	13	4, 5, 6, 12	H3, 6, 7a
C14	25.94			14a, 14b	7a	
H14a	1.547	m	15a, 15b	14	5, 15	
H14b	1.505	m	6, 7a, 15a, 15b	14		12
C15	21.48			15a, 15b	H3, 6, 7a, 7b, 9, 14a	
H15a	1.186	m	H3, 14a, 14b, 15b	15	3	10
H15b	0.896	m	7a, 14a, 14b, 15a	15	3, 7	2b

HSQC of 10

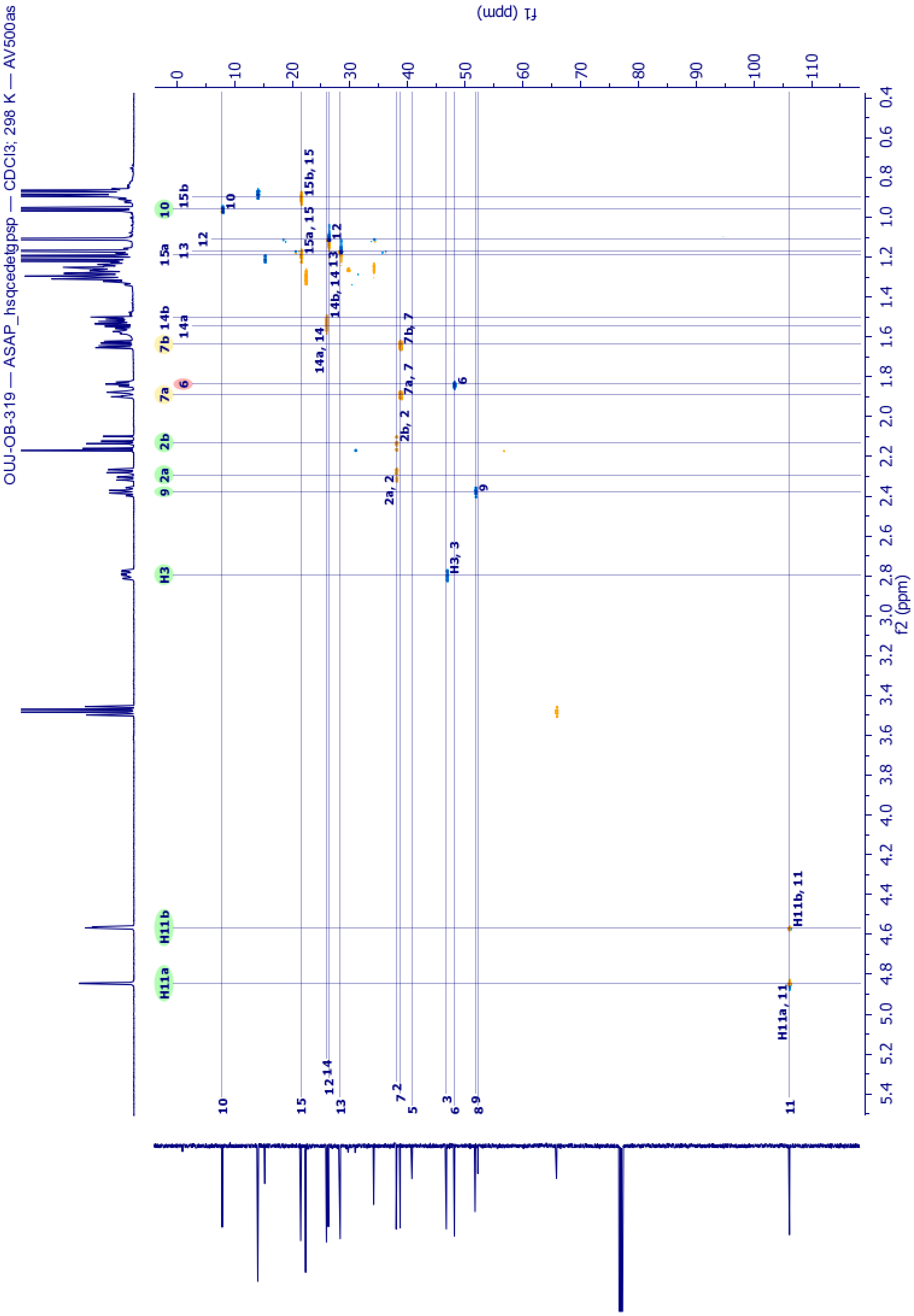

OUJ-OB-319 — ASAP_hsqcedetgpsp — CDCl3; 298 K — AV500as
f1 (ppm)
f2 (ppm)

HMQC of 10

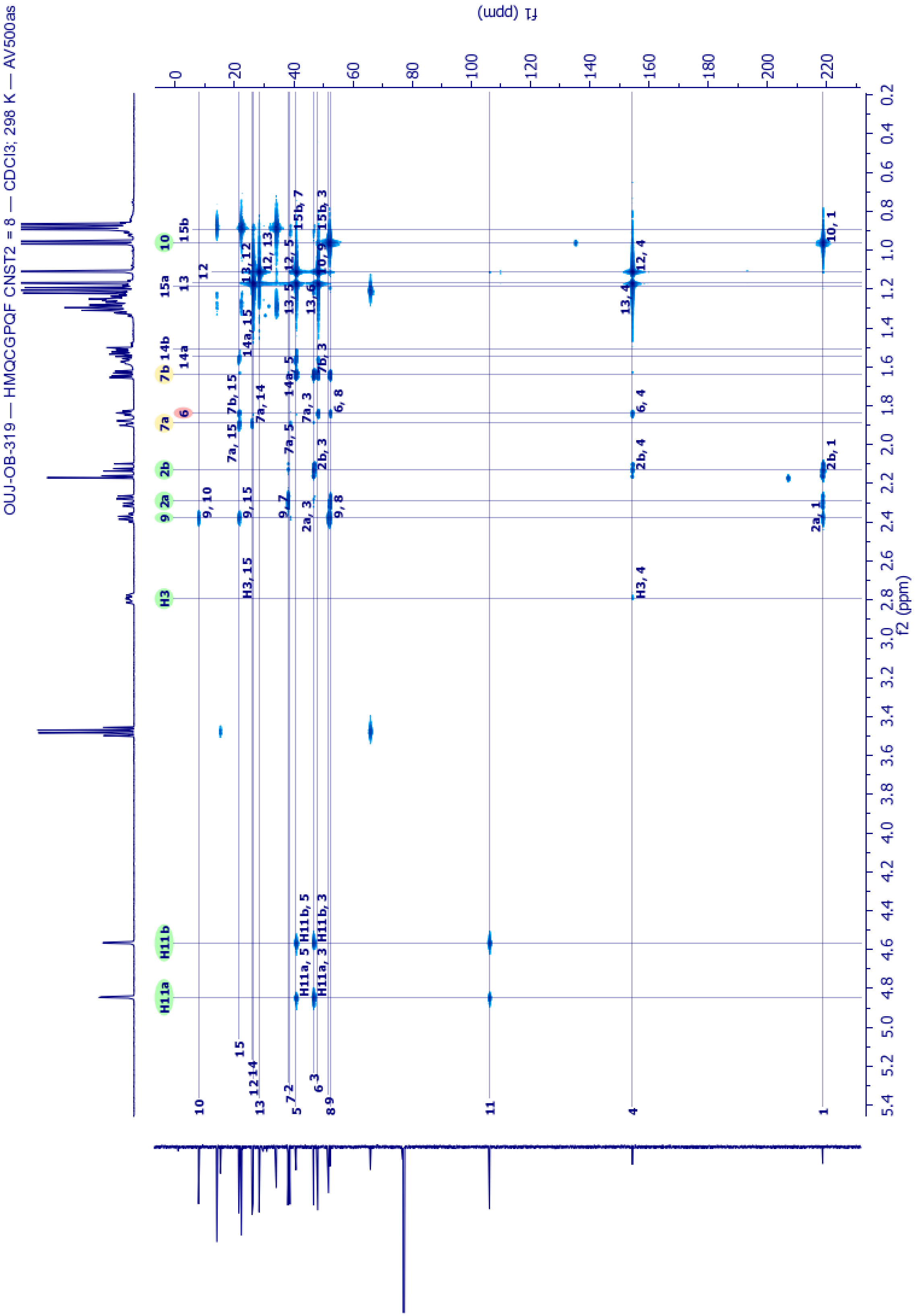

^{1}H-^{1}H COSY of 10

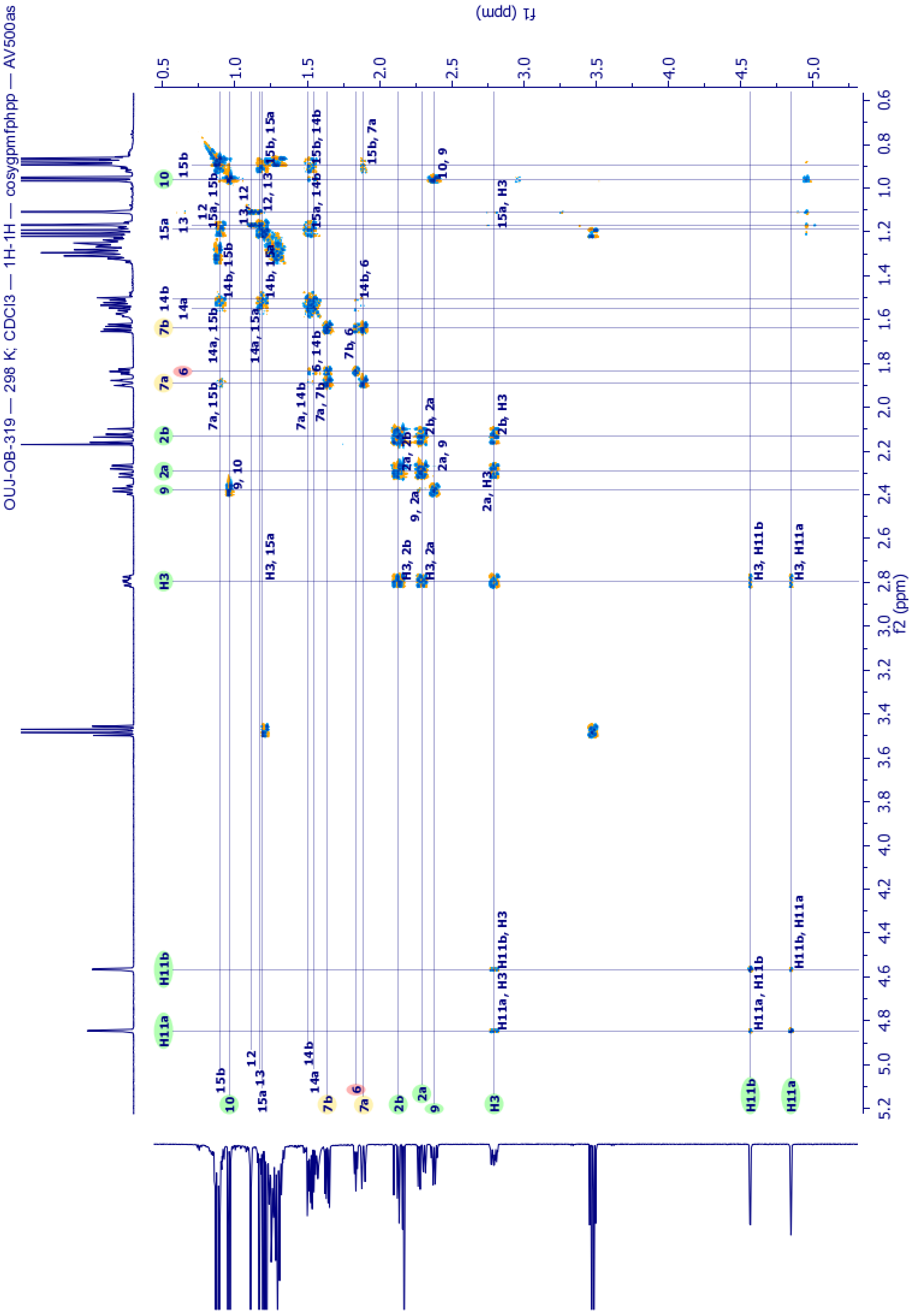

[1]H-^{1}H NOESY of 10

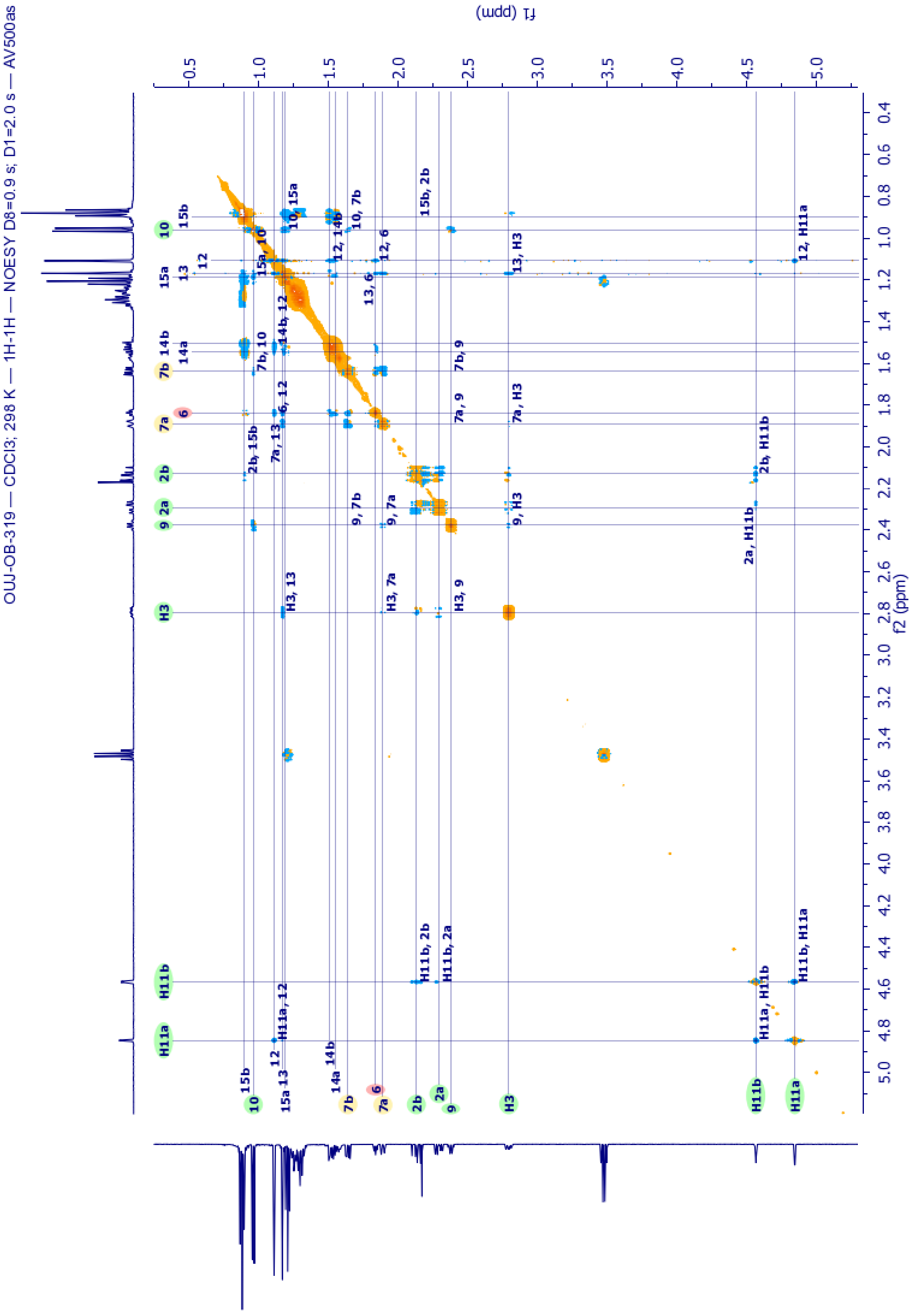

^{1}H NMR (500 MHz, $CDCl_3$) and ^{13}C NMR (125 MHz, $CDCl_3$) of 24

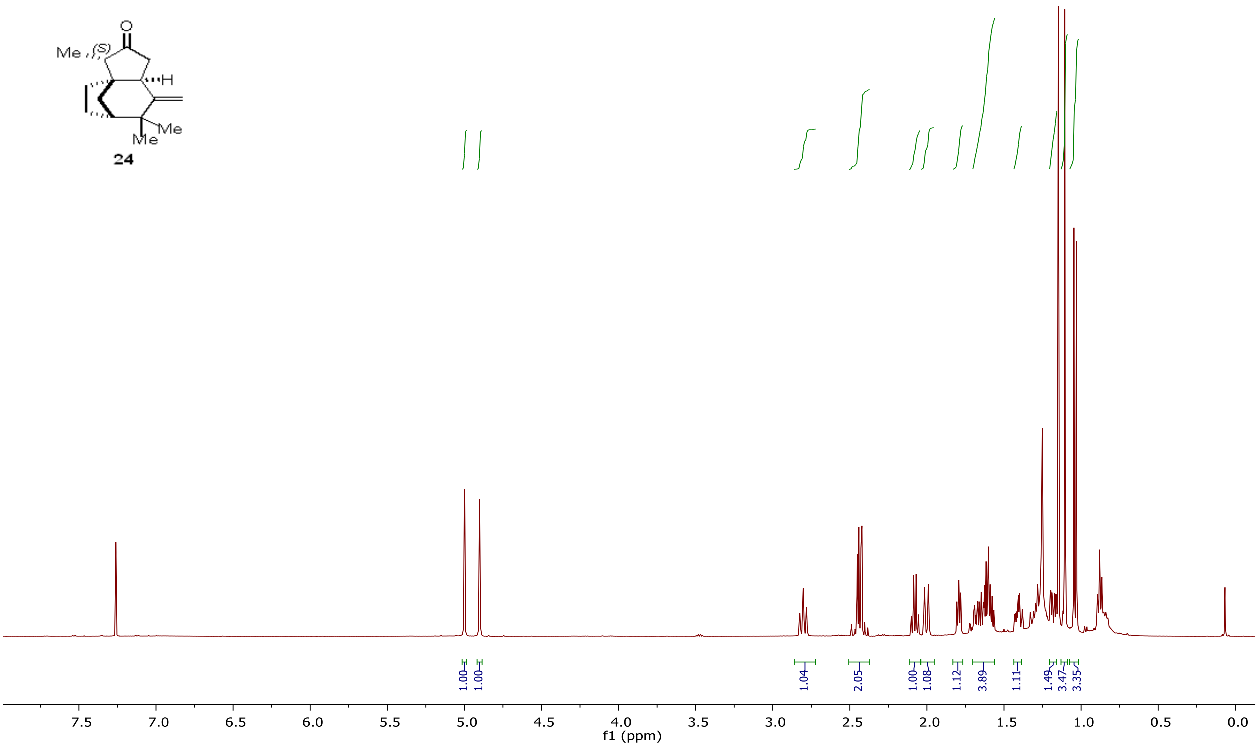

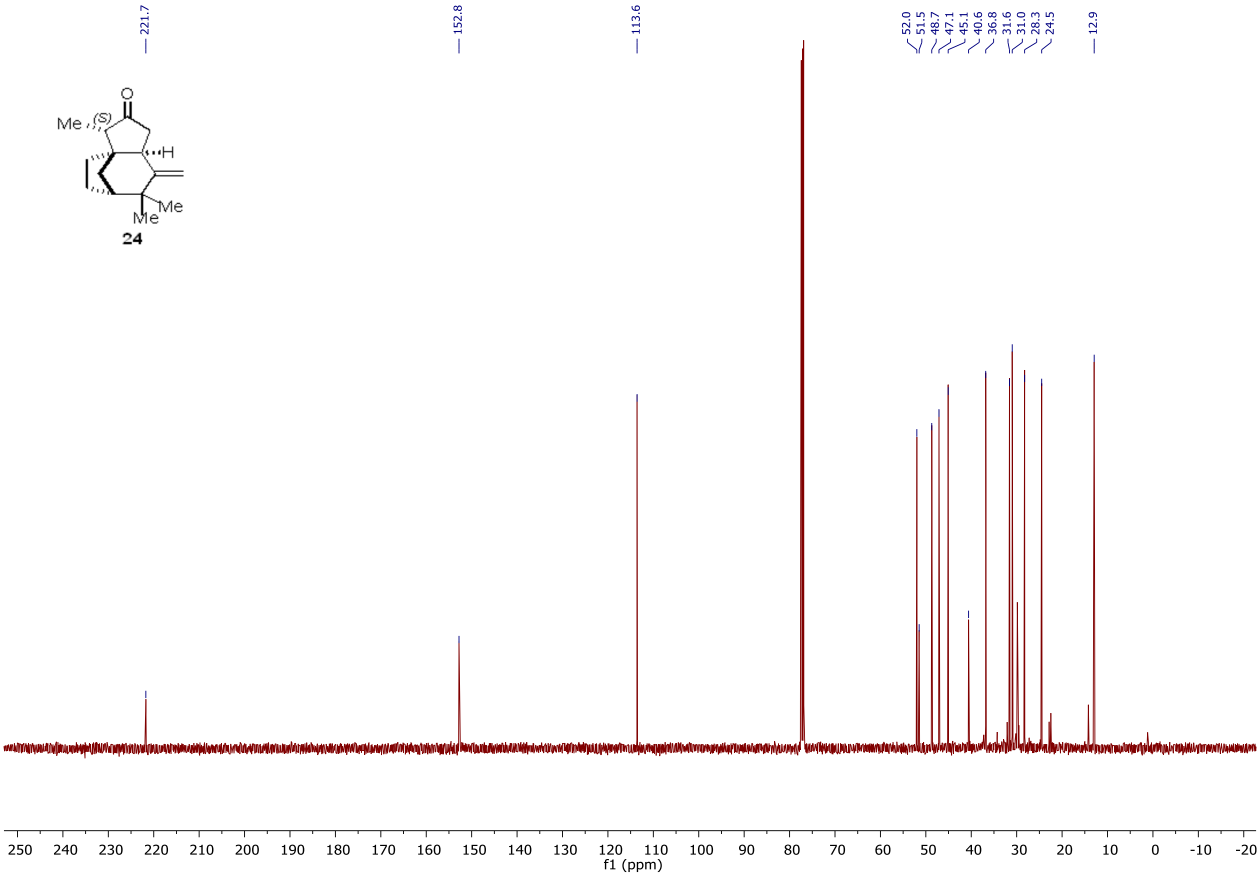

The 2D NMR overview analysis of 24

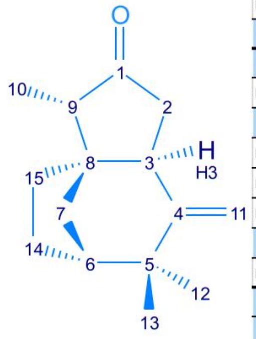

OUJ-OB-330

$CDCl_3$; 298 K; AV500as; 5 mg

Atom	δ	J	COSY	HSQC	HMQC	NOESY
C1	221.62				2a, 2b, 9, 10	
C2	44.91			2a, 2b	H3	
H2a	2.459	d 19.0(2b), d 0.8(9), d 11.7(H3)	H3, 2b, 9	2	1, 3, 4	7a, 11b, 13
H2b	2.412	d 19.0(2a), d 9.8(H3)	H3, 2a	2	1, 3, 8	7a, 11b, 13
C3	48.53			H3	2a, 2b, 9, 11a, 11b	
H3	2.803	d 2.0(7b), d 11.7(2a), d 9.8(2b), d 0.8(11b)	2a, 2b, 7b, 11b	3	2, 4, 5, 7, 11, 15	10, 11b, 15b
C4	152.63				H3, 2a, 6, 11a, 11b, 12, 13	
C5	40.42				H3, 6, 7a, 11a, 11b, 12, 13, 14a, 14b	
C6	46.94			6	12, 13	
H6	1.793	d 5.3(7b), d 0.8(11b), d 7.0(14a)	7b, 11b, 14a	6	4, 5, 7, 8, 12, 14, 15	7a, 7b, 12, 13
C7	36.64			7a, 7b	H3, 6, 9, 14b, 15b	
H7a	2.003	d 12.3(7b), d 2.1(14b), d 2.1(15b)	7b, 14b, 15b	7	5, 14, 15	2a, 2b, 6
H7b	1.180	d 2.0(H3), d 12.3(7a), d 5.3(6)	H3, 6, 7a	7	15	6, 9, 14a, 15a
C8	51.30				2b, 6	
C9	51.82			9	10	
H9	2.078	q 7.8(10), d 0.8(2a)	2a, 10	9	1, 3, 7, 10	7b, 15a
C10	12.77			10	9	
H10	1.039	d 7.8(9)	9	10	1, 9	H3, 15a, 15b
C11	113.42			11a, 11b	H3	
H11a	4.999	d 1.5(11b)	11b, 12	11	3, 4, 5, 13	12, 13
H11b	4.901	d 1.5(11a), d 0.8(H3), d 0.8(6)	H3, 6, 11a	11	3, 4, 5, 13	H3, 2a, 2b
C12	28.08			12	6, 13	
H12	1.105	s	11a, 13	12	4, 5, 6, 13	6, 11a, 14a, 14b
C13	31.42			13	11a, 11b, 12	
H13	1.147	s	12	13	4, 5, 6, 12	2a, 2b, 6, 11a
C14	24.32			14a, 14b	6, 7a, 15a	
H14a	1.690	m	6, 14b, 15a, 15b	14	5, 15	7b, 12
H14b	1.617	m	7a, 14a, 15a, 15b	14	5, 7, 15	12
C15	30.81			15a, 15b	H3, 6, 7a, 7b, 14a, 14b	
H15a	1.595	m	14a, 14b, 15b	15	14	7b, 9, 10
H15b	1.404	m	7a, 14a, 14b, 15a	15	7	H3, 10

Due to the strong coupling and overlap of H2a with H2b, it was not possible to distinguish their NOESY correlations and unambiguously determine their respective orientation.

HSQC of 24

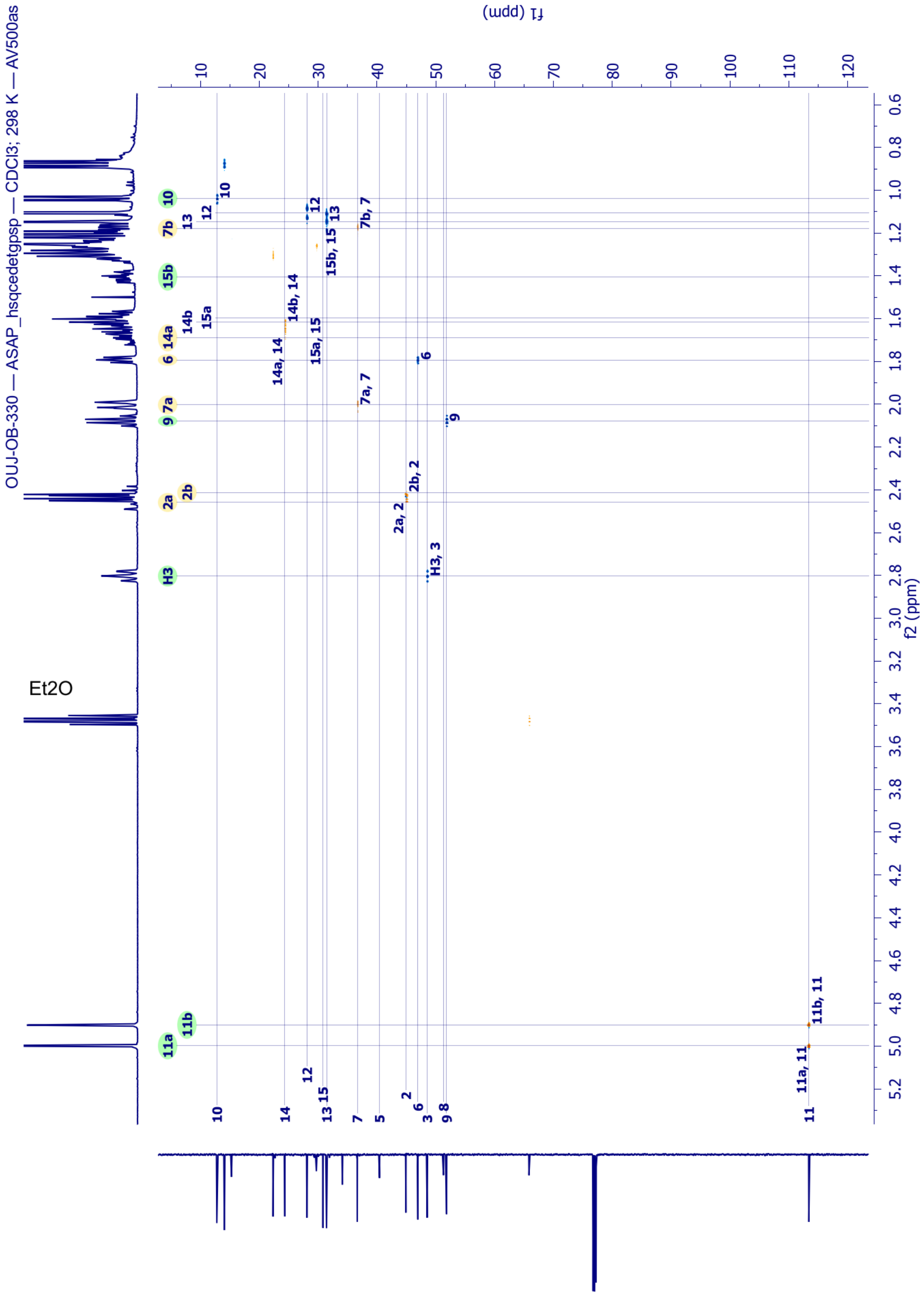

OUJ-OB-330 — ASAP_hsqcedetgpsp — CDCl3; 298 K — AV500as
Et2O
f1 (ppm)
f2 (ppm)

HMBC of 24

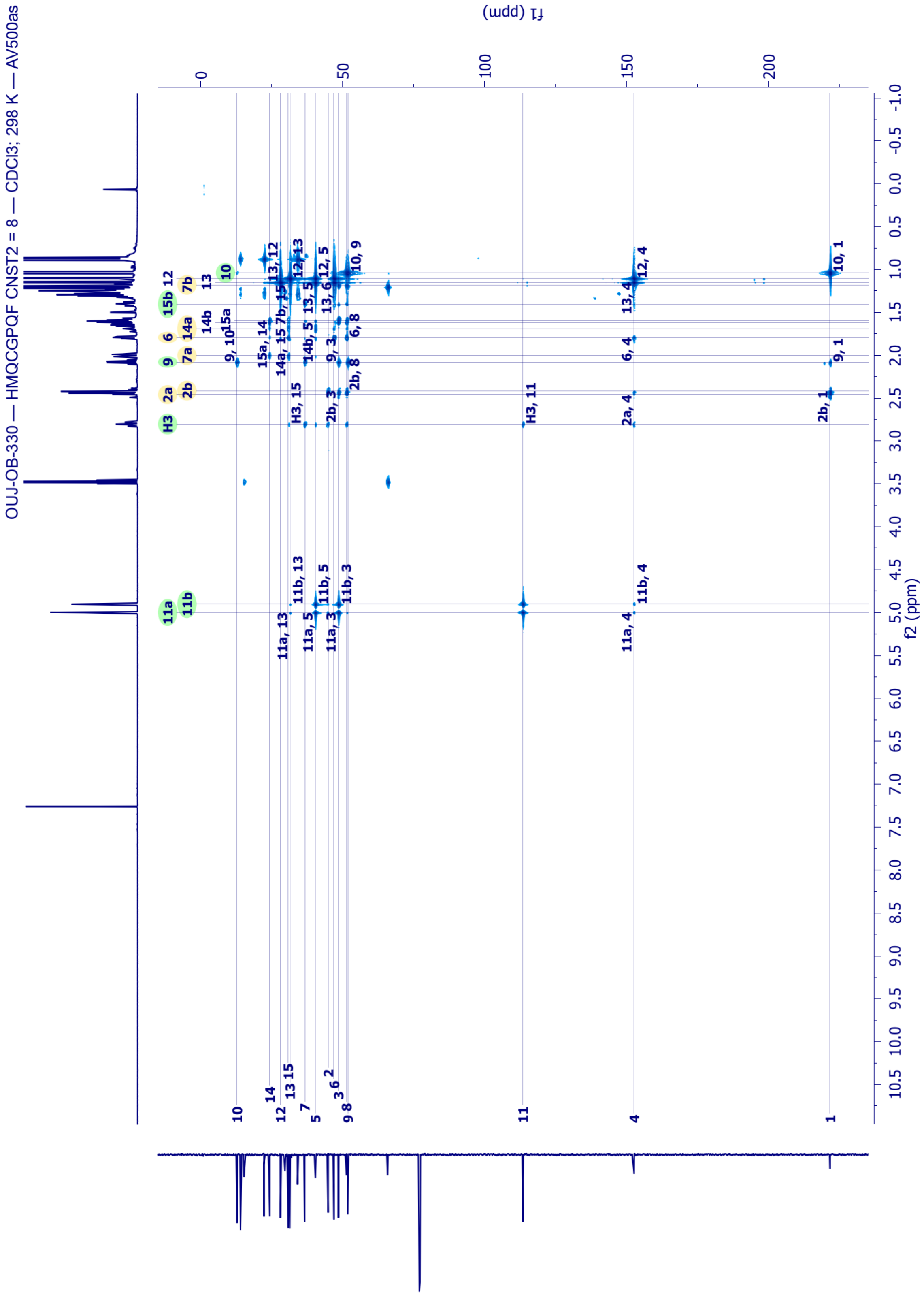

^1H-^1H COSY of 24

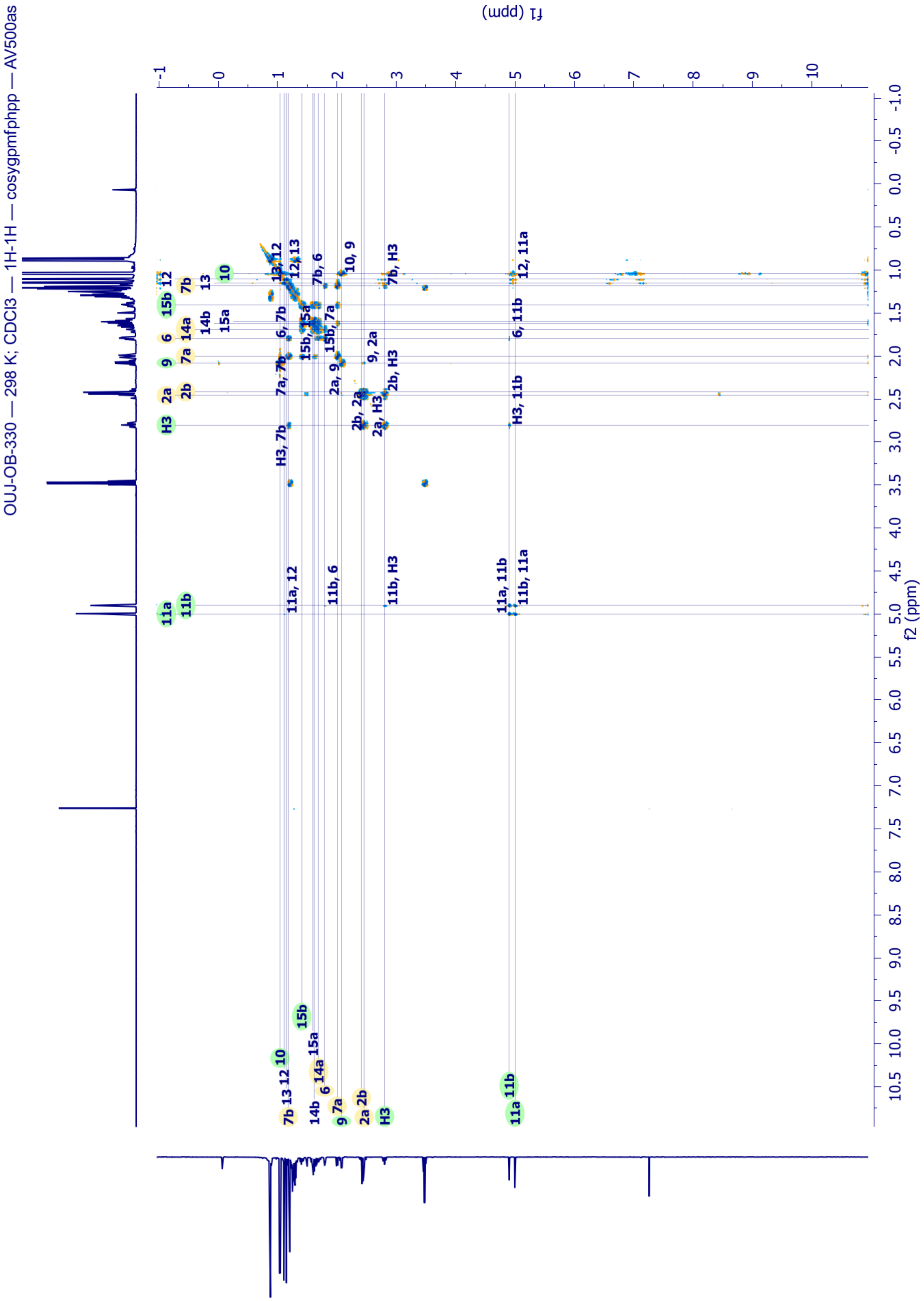

^{1}H-^{1}H NOESY of 24

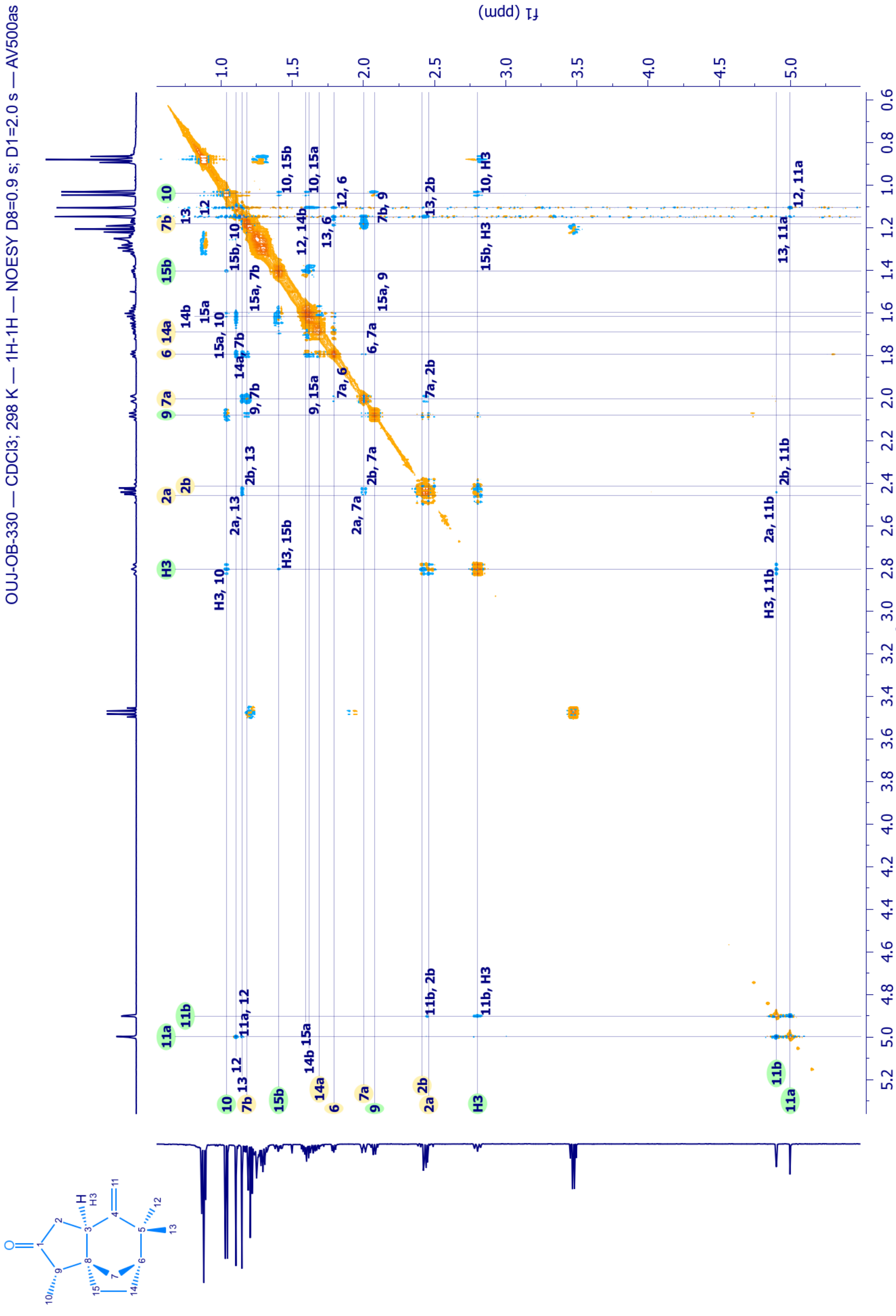

^{1}H NMR (500 MHz, $CDCl_3$) and ^{13}C NMR (125 MHz, $CDCl_3$) of 25

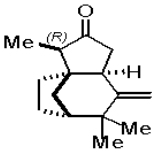

25

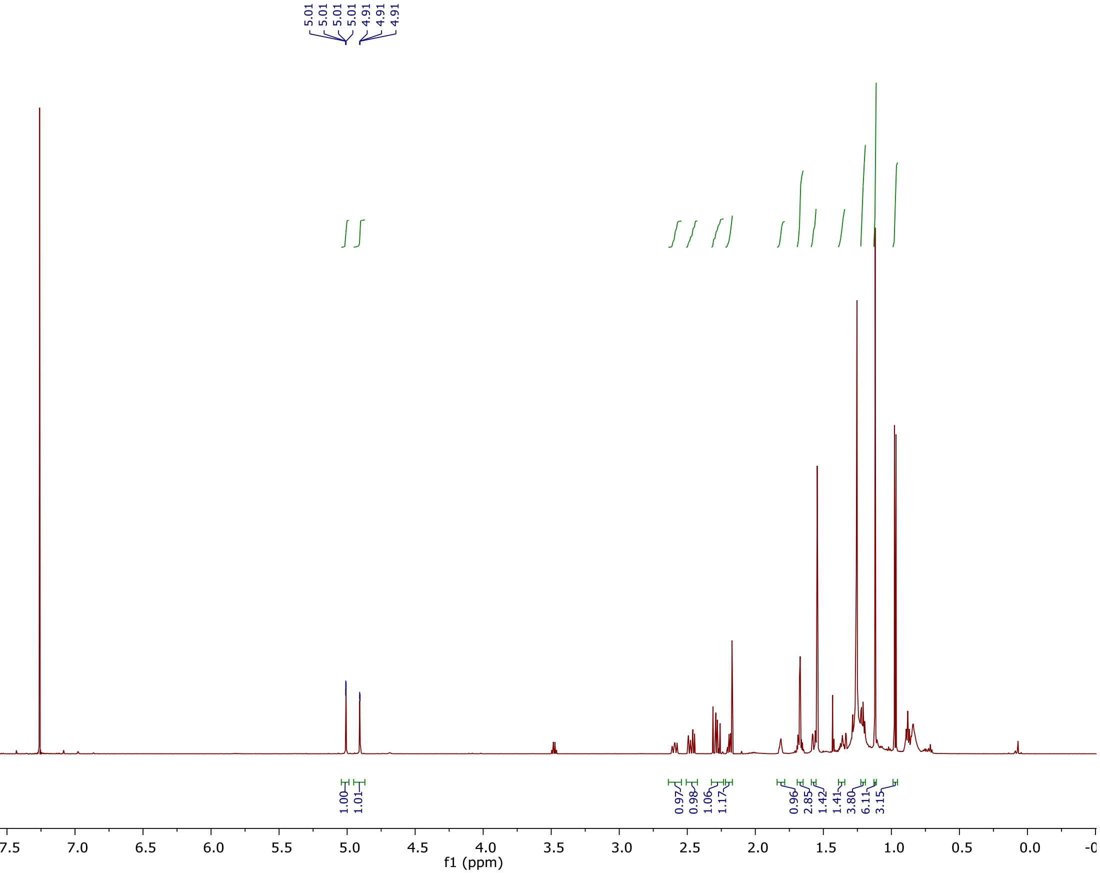

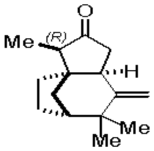

25

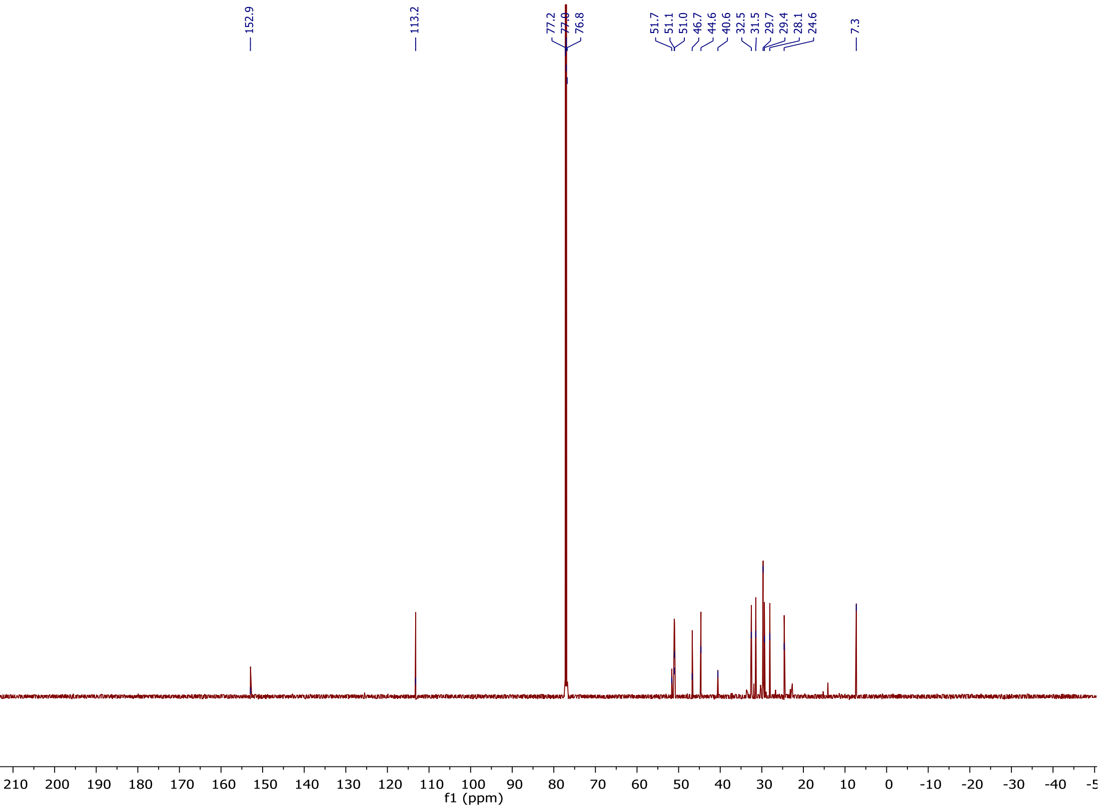

The 2D NMR overview analysis of 25

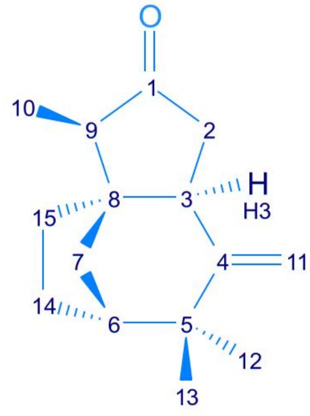

OUJ-OB-320

$CDCl_3$; 298 K; AV600a; 3 mg

Atom	δ	J	COSY	HSQC	HMQC	NOESY
C1	219.28				2a, 2b, 9, 10	
C2	44.63			2a, 2b	3, 11a, 11b	
H2a	2.469	d 19.0(2b), d 8.9(3), d 1.2(9)	3, 2b, 9	2	1, 3, 8, 9	11b
H2b	2.285	d 19.0(2a), d 12.6(3)	3, 2a	2	1, 3, 4, 9	7a, 13
C3	51.05			3	2a, 2b, 7b, 11a, 11b, 15a, 15b	
H3	2.592	d 12.6(2b), d 8.9(2a), d 2.0(7b), d 0.8(11b)	2a, 2b, 7b, 11a, 11b	3	2, 4, 5, 7, 8, 11, 15	9, 11b, 15b
C4	152.94				3, 2b, 6, 11a, 11b, 12, 13	
C5	40.56				3, 7a, 7b, 11a, 11b, 12, 13, 14	
C6	46.74			6	12, 13	
H6	1.814	m	7a, 7b, 11b, 14, 15a, 15b	6	4, 8, 15	7a, 7b, 12, 13
C7	29.39			7a, 7b	3, 9, 14, 15a, 15b	
H7a	1.568	d 12.2(7b), d 2.3(15b)	6, 7b, 15b	7	5, 14, 15	2b, 6, 7b, 10, 13
H7b	1.209	d 12.2(7a), d 5.4(6), d 2.0(3)	3, 6, 7a	7	3, 5, 8, 15	6, 7a, 10, 14, 15a
C8	51.66				3, 2a, 6, 7b, 9, 10, 14, 15a, 15b	
C9	50.98			9	2a, 2b, 10, 15a	
H9	2.187	q 7.0(10), d 1.2(2a)	2a, 10	9	1, 7, 8, 10, 15	3, 10, 15a, 15b
C10	7.29			10	9	
H10	0.972	d 7.0(9)	9	10	1, 8, 9	7a, 7b, 9, 15a
C11	113.21			11a, 11b	3	
H11a	5.009	d 1.5(11b)	3, 11b	11	2, 3, 4, 5, 13	11b, 12, 13
H11b	4.907	d 1.5(11a), t 0.8(3, 6)	3, 6, 11a	11	2, 3, 4, 5, 13	3, 2a, 11a
C12	28.08			12	13	
H12	1.119	s		12	4, 5, 6, 13	6, 11a, 14
C13	31.45			13	11a, 11b, 12	
H13	1.119	s		13	4, 5, 6, 12	2b, 6, 7a, 11a
C14	24.63			14	7a, 15a, 15b	
H14	1.70-1.64	m (overlapped)	6, 15b	14	5, 7, 8, 15	7b, 12
C15	32.52			15a, 15b	3, 6, 7a, 7b, 9, 14	
H15a	1.70-1.64	m (overlapped)	6, 15b	15	3, 7, 8, 9, 14	7b, 9, 10
H15b	1.362	m	6, 7a, 14, 15a	15	3, 7, 8, 14	3, 9

HSQC of 25

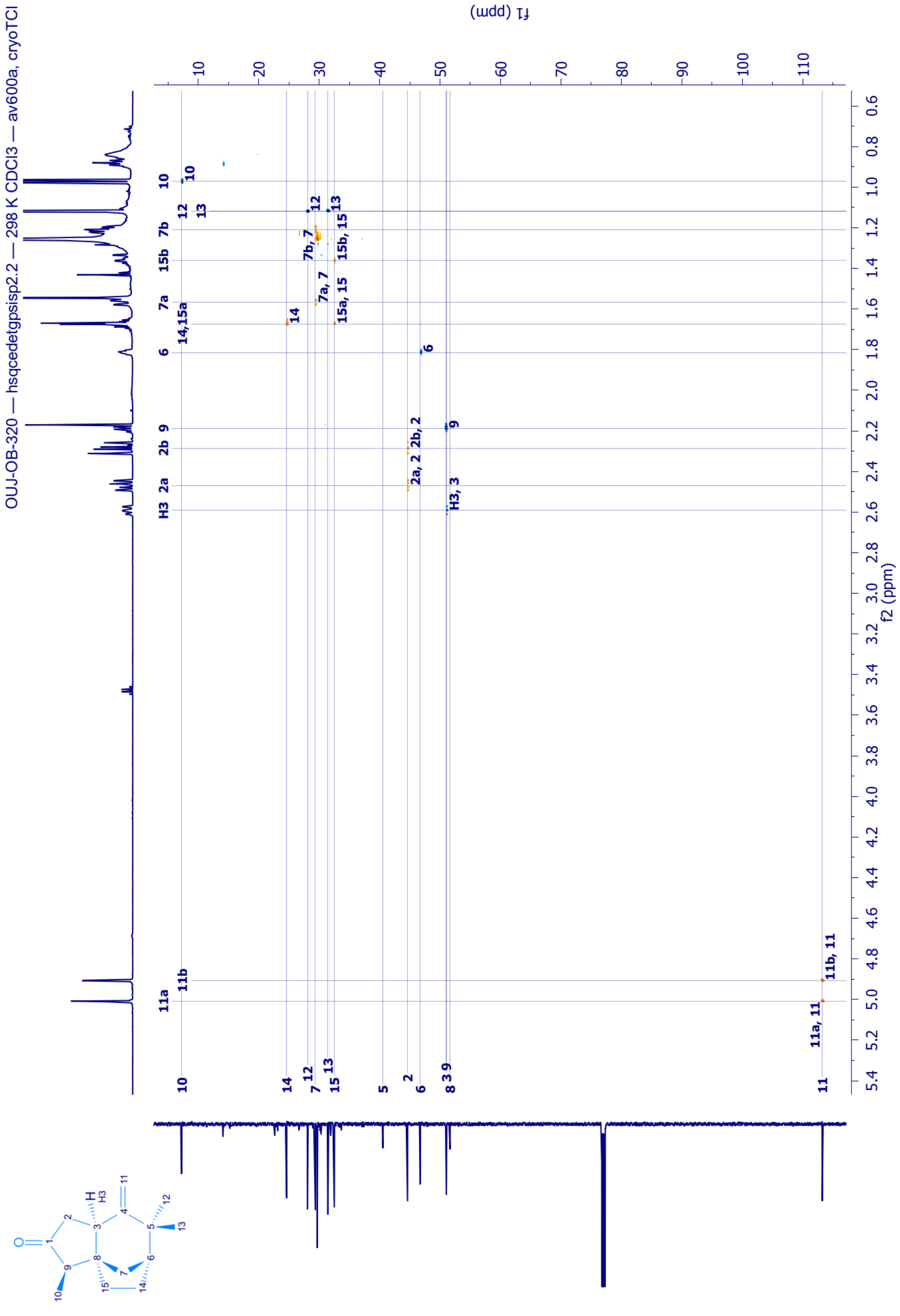
OUJ-OB-320 — hsqcedetgpsisp2.2 — 298 K CDCl3 — av600a, cryoTCI
f1 (ppm)
f2 (ppm)

HMQC of 25

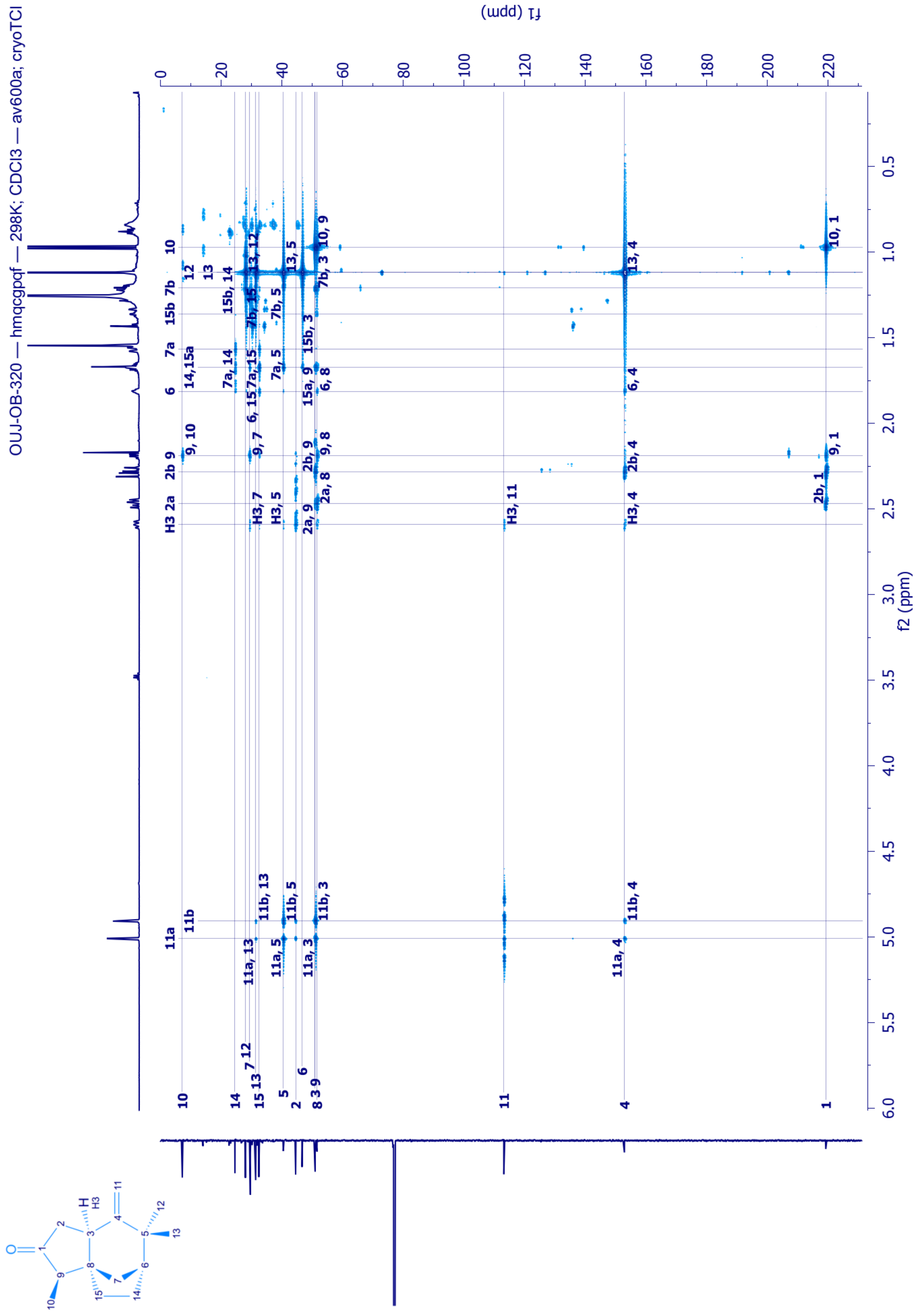

^{1}H-^{1}H COSY of 25

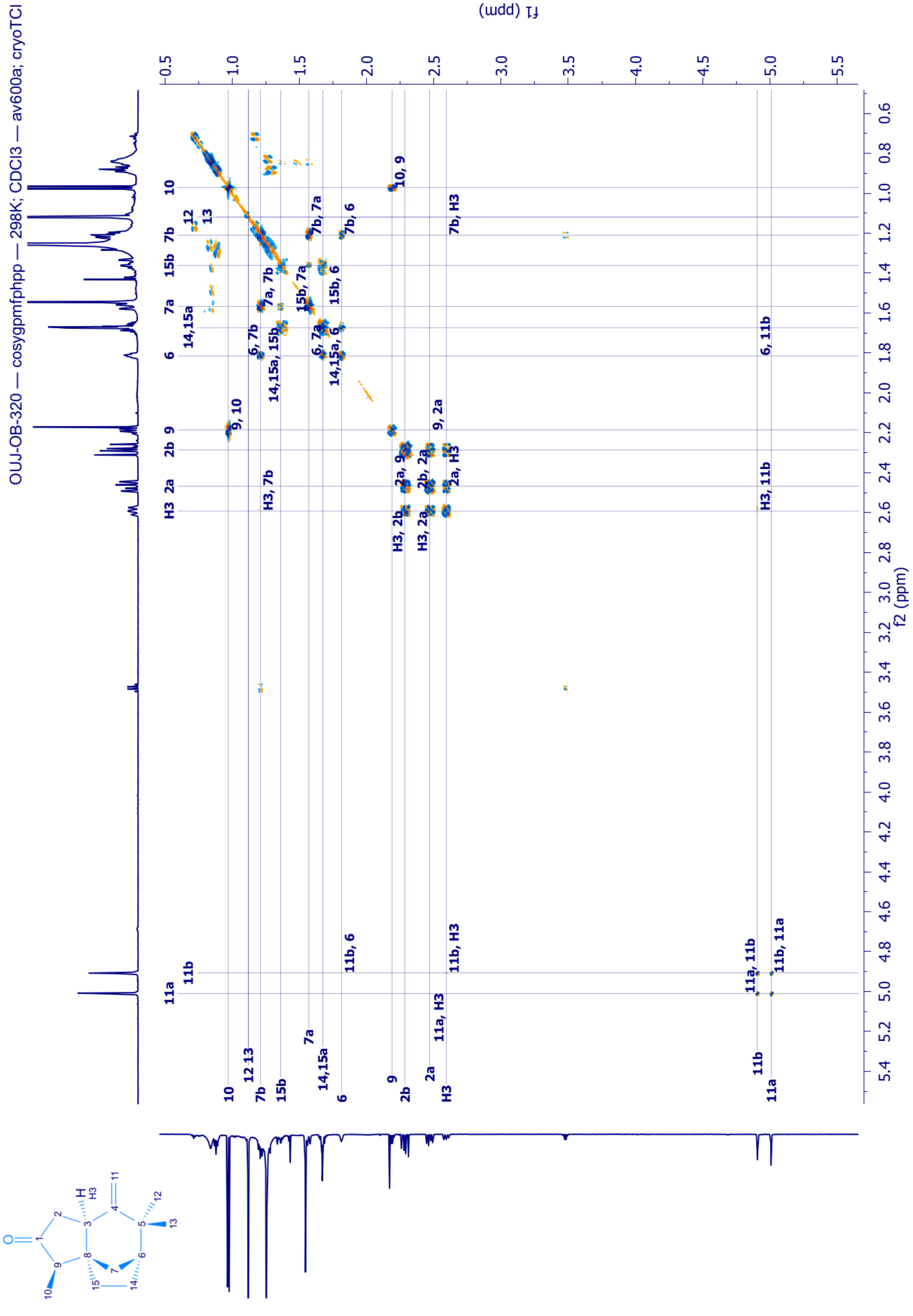

^{1}H-^{1}H COSY of 25

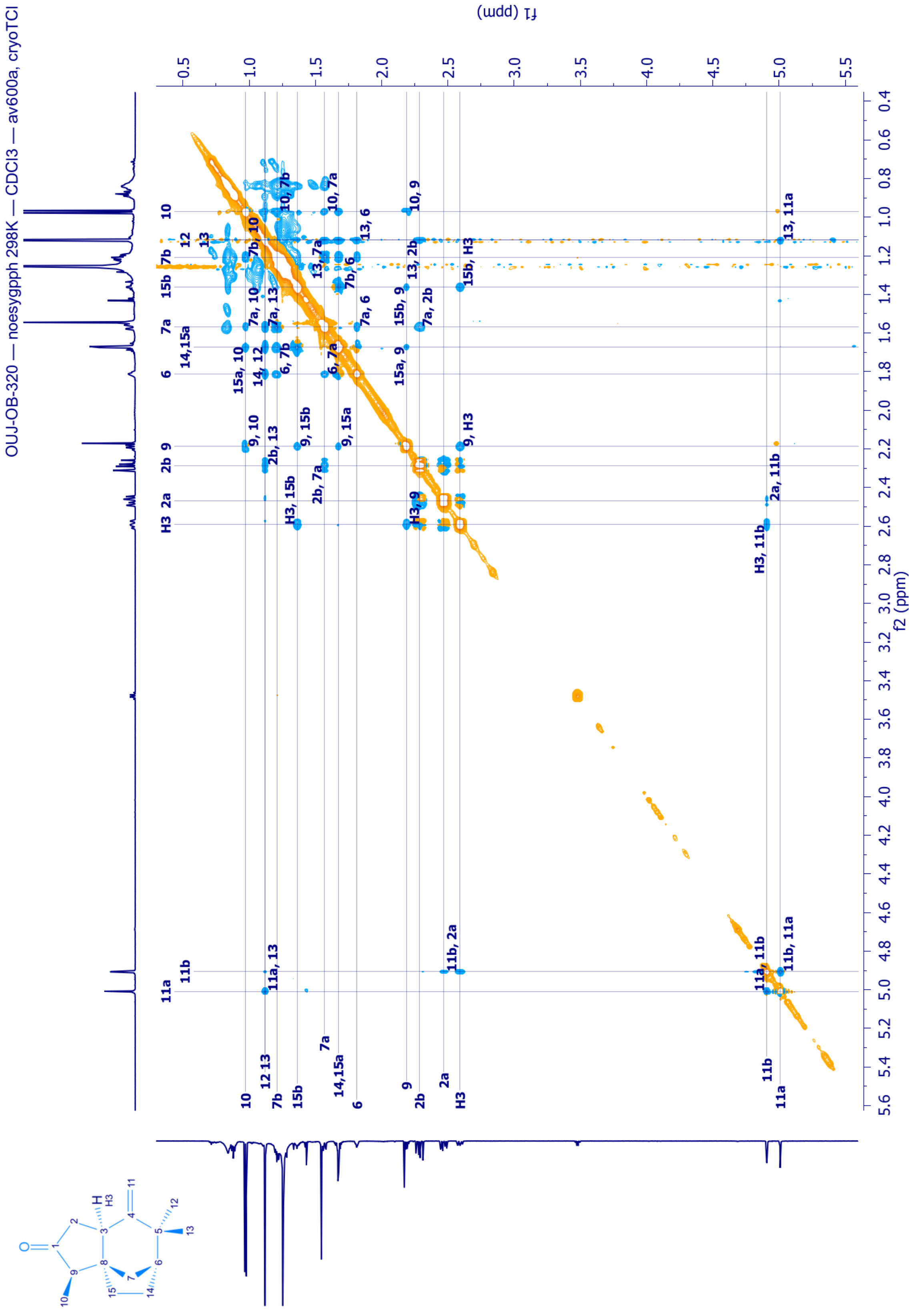

^{1}H NMR (500 MHz, CDCl$_3$) and ^{13}C NMR (125 MHz, CDCl$_3$) of 28

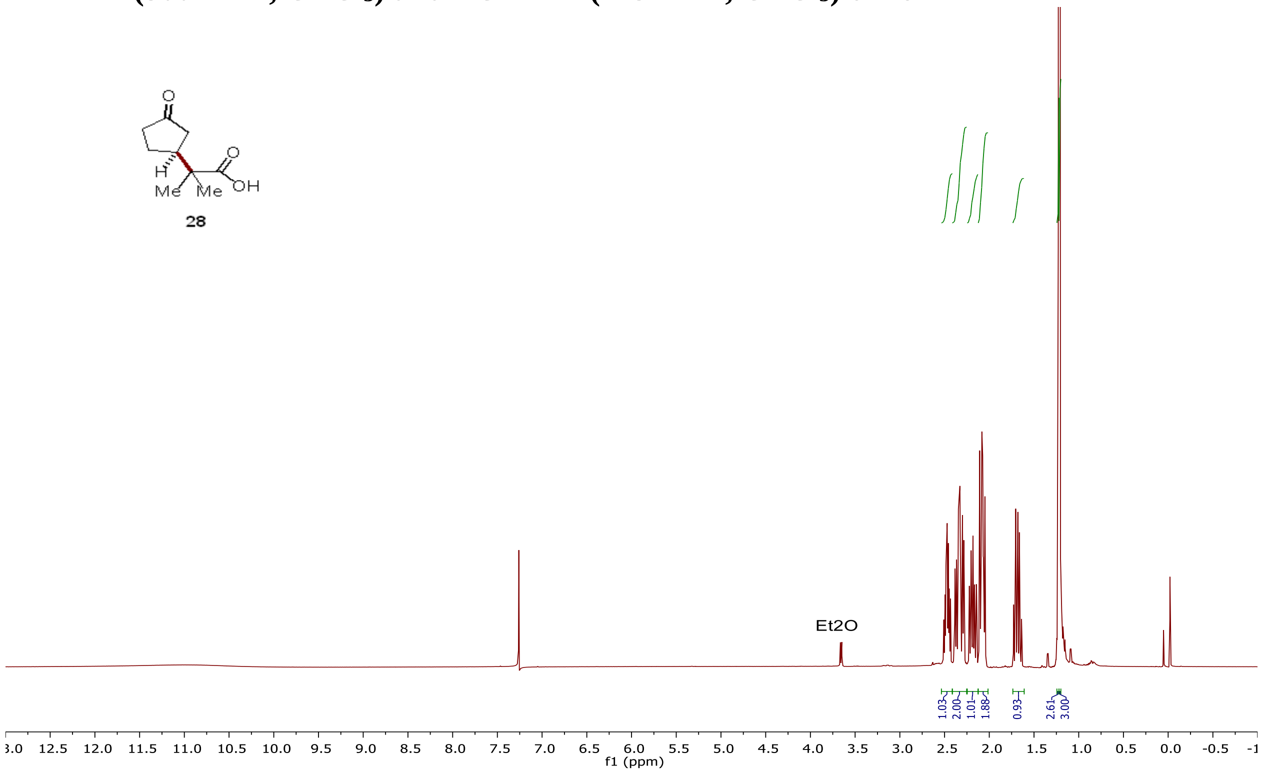

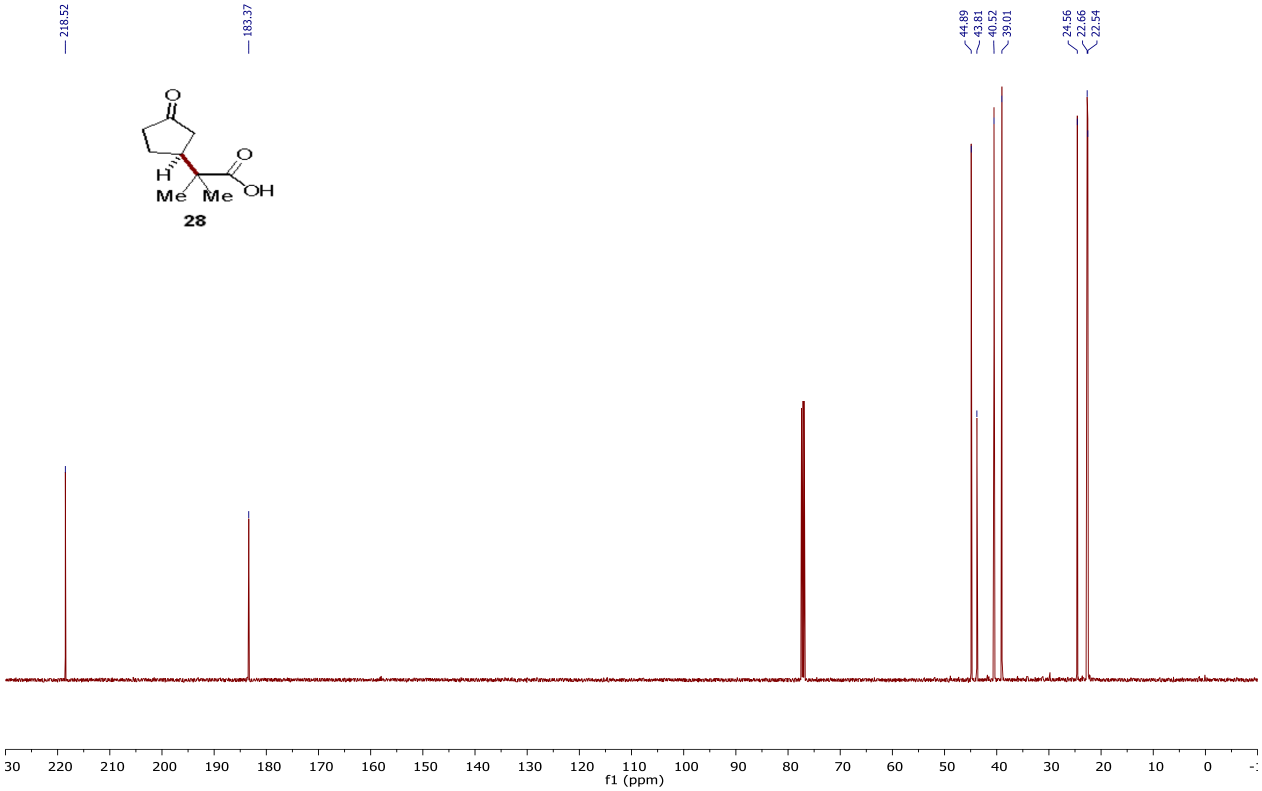

HPLC and GC trace

HPLC of 15

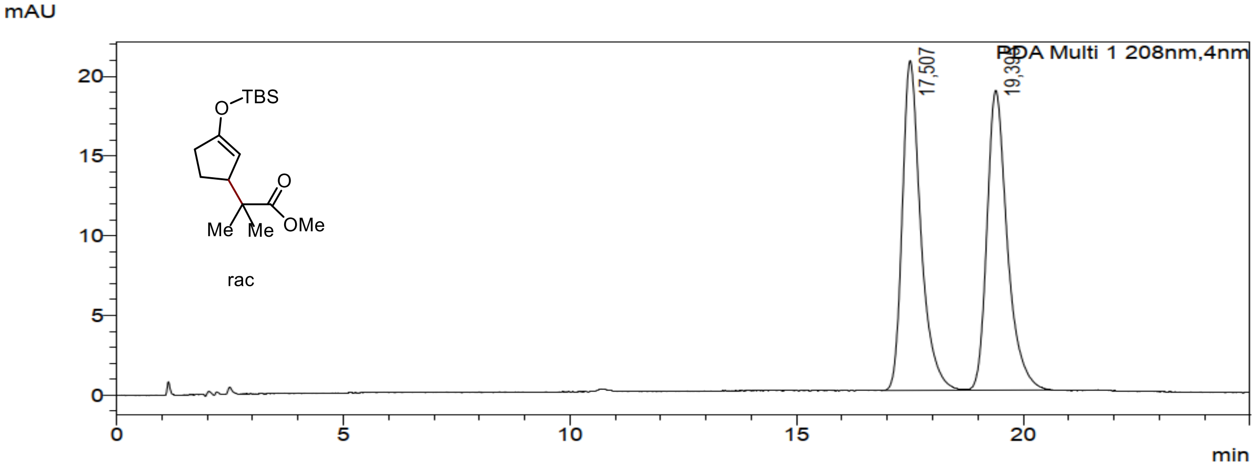

<Peak Table>

PDA Ch1 208nm

Peak#	Ret. Time	Area%	Area	Height
1	17,507	49,934	584702	20674
2	19,395	50,066	586246	18793
Total		100,000	1170949	39467

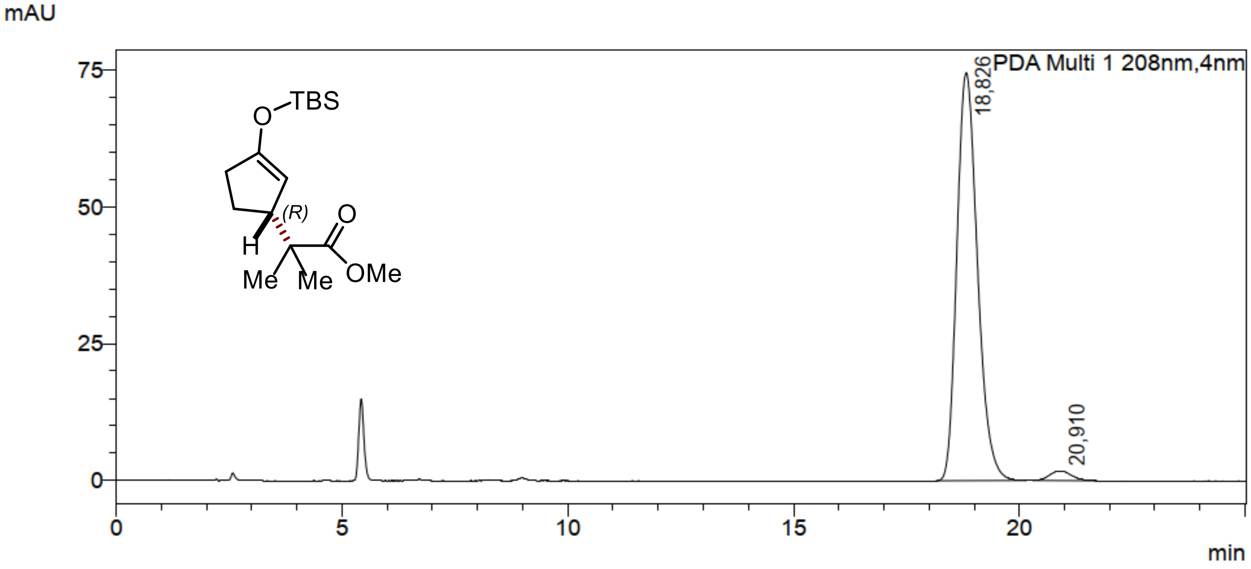

<Peak Table>

PDA Ch1 208nm

Peak#	Ret. Time	Area	Area%
1	18,826	2363050	97,562
2	20,910	59053	2,438
Total		2422103	100,000

GC trace of 21 and 27

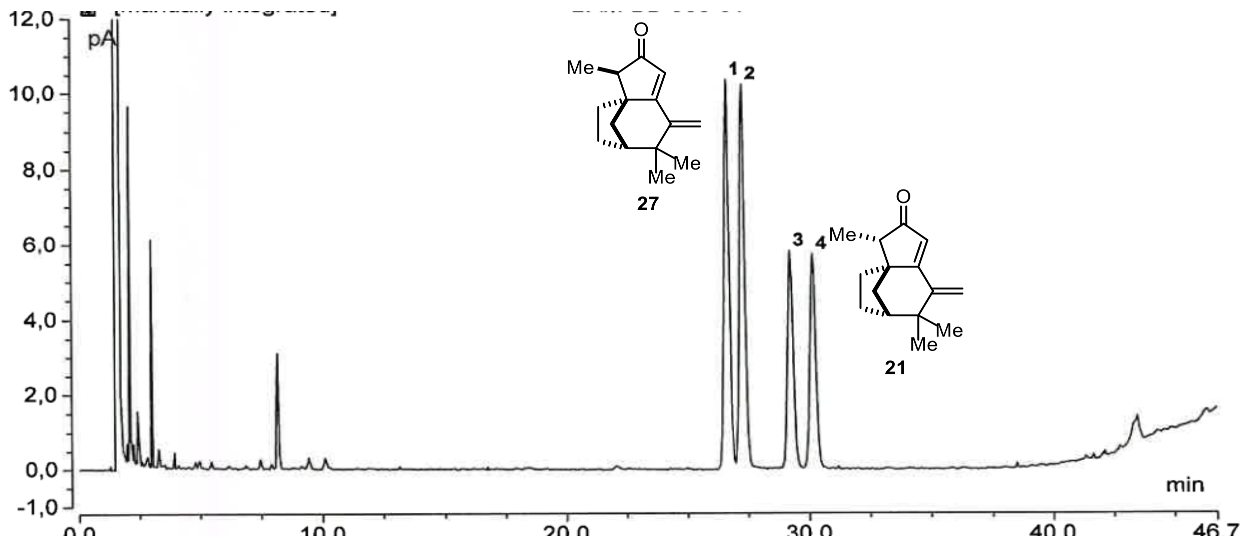

No.	Ret.Time	area-%	Peak Name
	min	%	
1	26,65	30,85	
2	27,30	30,84	
3	29,24	19,10	
4	30,17	19,21	

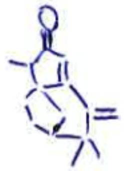

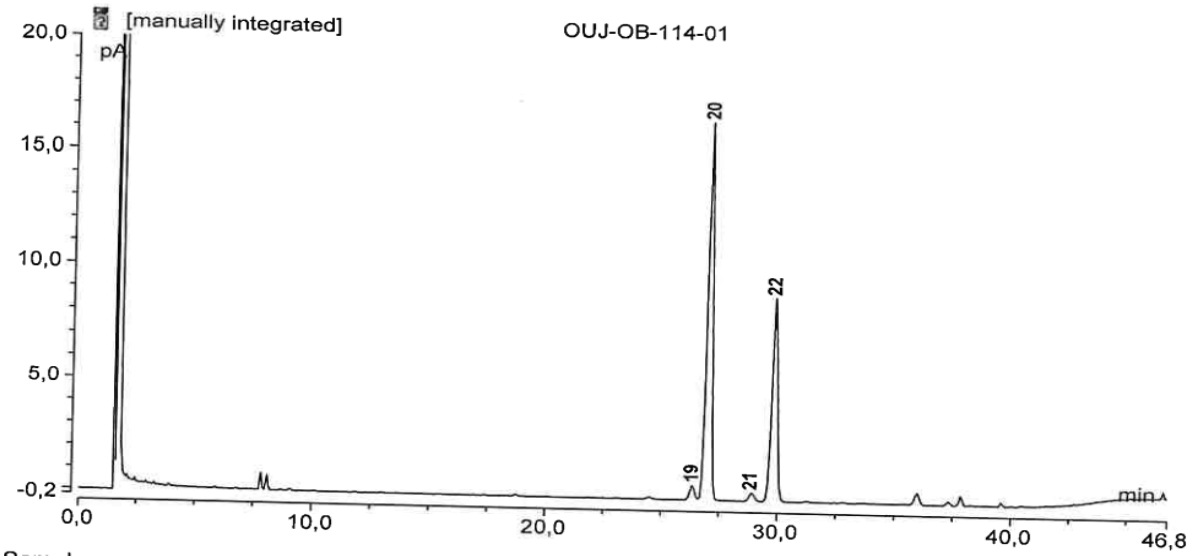

No.	Ret.Time	Rel.Area	Peak Name
	min	%	
19	26,35	2,27 .	
20	27,01	60,82 .	
21	28,91	1,33 .	
22	29,83	35,59 .	

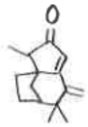

GC trace of 21 after the HPLC separation

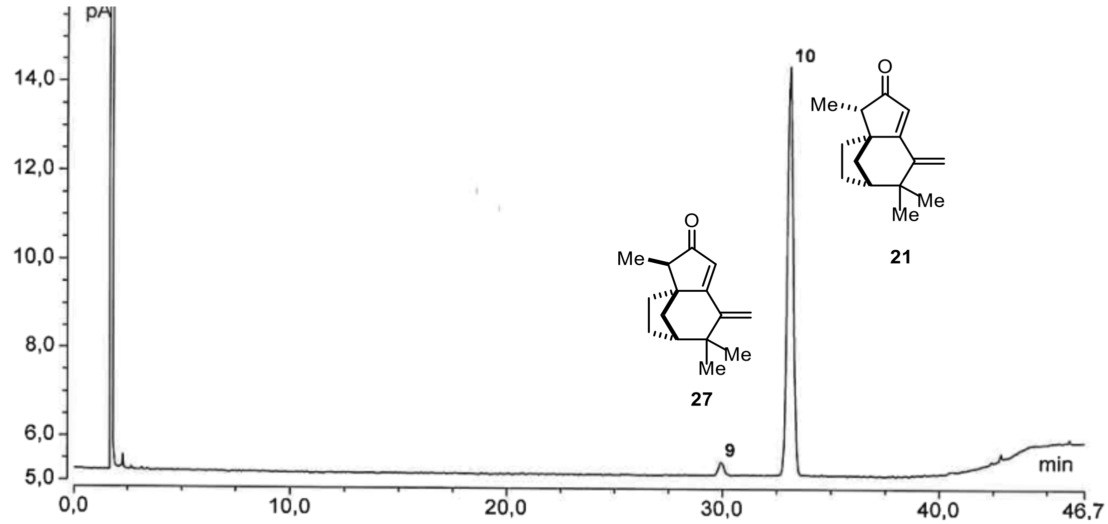

No.	Ret.Time min	Rel.Area %	Peak Name
9	29,96	2,78	.
10	33,14	97,22	.

GC trace of 27 after the HPLC separation

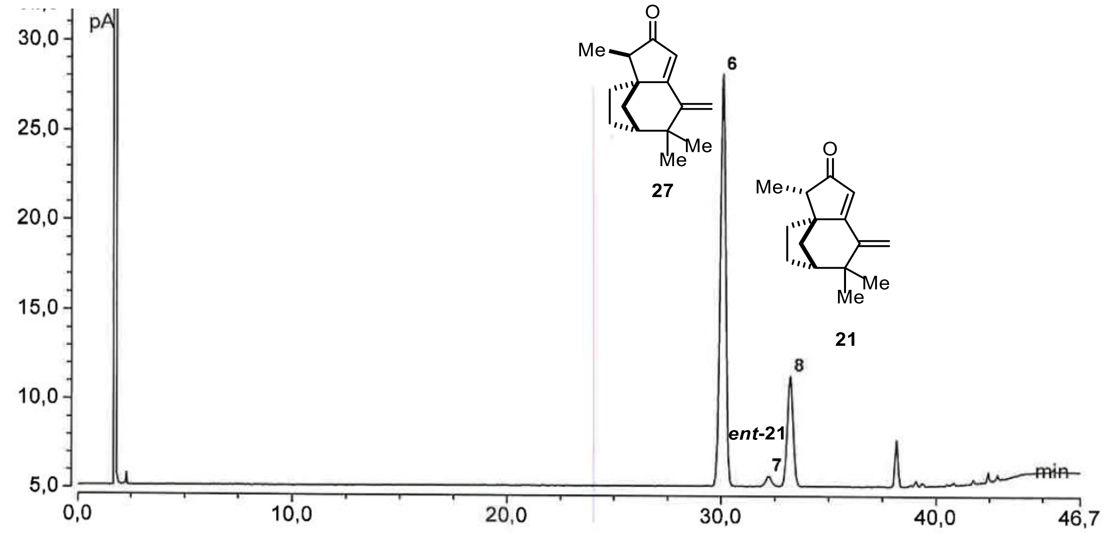

No.	Ret.Time min	Rel.Area %	Peak Name
6	30,10	75,87	.
7	32,21	1,98	.
8	33,23	22,15	.

GC of 10

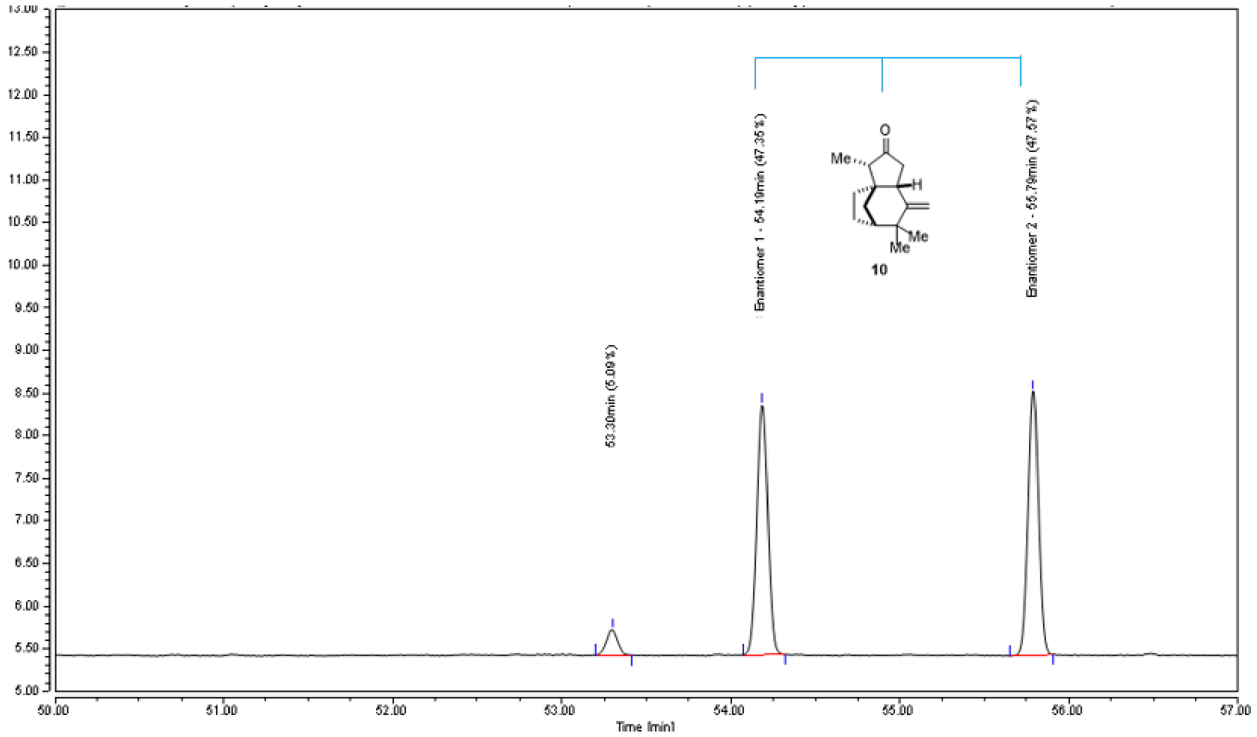

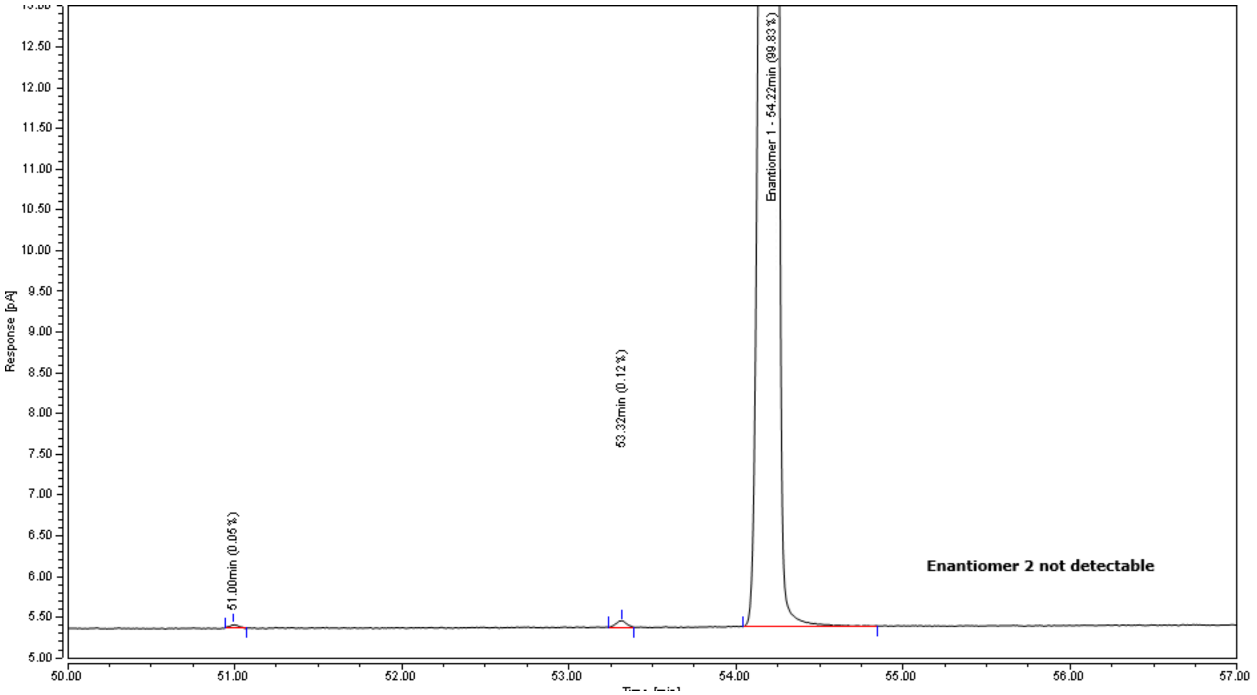

Chiral GC method (Trace 1310 GC with autosampler TriPlus 100LS): split ratio 1:1 between FID & sniffing port, 1µl injection volume (1000ng/µl), split 20:1, hot needle injection technique, injector 220°C, carrier gas H_2, constant flow 1.5ml/min, column 30 m x 0.25 mm x 0.12 µm Astec Chiraldex G-DP (Sigma-Aldrich, P/N 78033AST, S/N 60453-02B), temperature program

2min@50°C-2°C/min-2min@200°C, FID base 250°C, air 350ml/min, H_2 35ml/min, N_2 make-up 40ml/min, Chromeleon 7.2.

GC odour threshold determination – Chromatography & Olfactometry

Structure:

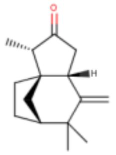

Operator: Sandro Dossenbach
Test Date: 07.01.2020
GC Threshold Comment: N/A

	Peak A<br>*Major odor impact*	Peak B<br>*Minor odor impact*
**tR [min]**	1.04	
**Relative Area %**	100	
**Panelists**	**Lowest amount [ng] detected**	**Lowest amount [ng] detected**
S.D.	0.05	
S.T.	0.0125	
P.K.	0.025	
S.J.	0.05	
N.J.	0.025	
**Geometric Mean in Sample**	**0.029**	
**Standard Deviation Sample**	0.017	
**Geometric Mean Pure Peak**	**0.029**	

GC threshold value of compound **10** is 0.029 ng.

Structure:

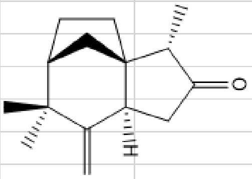

Panelists	**main peak**	sec. peak
	**value [ng]**	value [ng]
	**100 w %**	**w %**
S.D.	1.3	
P.K.	1.3	
S.J.	1.3	
D.L.	1.3	
A.G.	1.3	
G.B.	1.3	
D.B.	1.3	
**GEOMEAN to 100%**	**1.3**	**#NUM!**
STANDEV	0.0	#DIV/0!
**GCTH of main peak**	**1.3**	**#NUM!**

GC threshold value of compound **24** is 1.3 ng.

Structure:

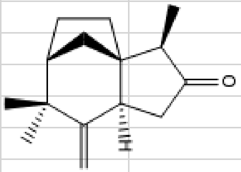

Panelists	main peak value [ng] 100 w %	sec. peak value [ng] w %
S.D.	0.163	
P.K.	0.163	
S.J.	0.163	
D.L.	1.625	
A.G.	0.163	
G.B.	0.163	
D.B.	0.163	
**GEOMEAN to 100%**	**0.226**	**#NUM!**
STANDEV	0.553	#DIV/0!
**GCTH of main peak**	**0.226**	**#NUM!**

GC threshold value of compound **25** is 0.23 ng.
